# Multispectral live-cell imaging with uncompromised spatiotemporal resolution

**DOI:** 10.1038/s41566-025-01745-7

**Published:** 2025-09-08

**Authors:** Akaash Kumar, Kerrie E. McNally, Yuexuan Zhang, Alex Haslett-Saunders, Xinru Wang, Jordi Guillem-Marti, David Lee, Buwei Huang, Sjoerd Stallinga, Robert R. Kay, David Baker, Emmanuel Derivery, James D. Manton

**Affiliations:** 1 MRC Laboratory of Molecular Biology, Cambridge, UK; 2 Institute for Protein Design, University of Washington, Seattle, WA USA; 3 Department of Imaging Physics, Delft University of Technology, Delft, Netherlands; 4 Present Address: Department of Materials Science and Engineering, Universitat Politècnica de Catalunya — BarcelonaTech (UPC), Barcelona, Spain

**Keywords:** Biological fluorescence, Microscopy, Imaging and sensing

## Abstract

Multispectral imaging is an established method to extend the number of colours usable in fluorescence imaging beyond the typical limit of three or four. However, standard approaches are poorly suited to live-cell imaging owing to the need to separate light into many spectral channels, and unmixing algorithms struggle with low signal-to-noise ratio data. Here we introduce an approach for multispectral imaging in live cells that comprises an iterative spectral unmixing algorithm and eight-channel camera-based image-acquisition hardware. This enables the accurate unmixing of low signal-to-noise ratio datasets captured at video rates, while maintaining diffraction-limited spatial resolution. We use this approach on a commercial spinning-disk confocal microscope and a home-built oblique-plane light-sheet microscope to image one to seven spectrally distinct fluorophore species simultaneously, using both fluorescent protein fusions and small-molecule dyes. We further develop protein-binding proteins (minibinders), labelled with organic fluorophores, and use these in combination with our multispectral imaging approach to study the endosomal trafficking of cell-surface receptors at endogenous levels.

## Abstract (web summary)

A tree-like arrangement of dichroic mirrors and multiple cameras coupled with an iterative spectral unmixing algorithm enables multispectral imaging of live cells in up to eight spectral channels with diffraction-limited spatial resolution and temporal resolution of 0.3 s for imaging a full cell volume.

## Main

Living cells contain thousands of different components, each with their own specific interaction partners and complex dynamics. Fluorescence microscopy has proven to be a powerful tool for studying the dynamics of life, as it provides background-free images of cells and tissues with molecular specificity. However, the broad spectra of typical fluorophores compatible with biological imaging limit the number of colours that can be employed without spectral overlap and crosstalk between colour channels. Accordingly, only two or three cellular components can typically be studied in the same sample.

Multispectral imaging with spectral unmixing provides a way around this limitation by computationally assigning light captured in discrete wavebands to different fluorophores^1^. However, conventional implementations of spectral unmixing^2–9^ suffer from three key problems that have limited their use, particularly in live-cell imaging: (1) light is chromatically separated into a large number of wavebands (for example, 32), leading to low signal levels in any one band; (2) illumination is provided as a spot or a line, which must be scanned across the field of view, leading to slow acquisition rates and high irradiances; (3) linear spectral unmixing algorithms deal poorly with noise, leading to inaccurately reconstructed data.

Recently, Valm and colleagues introduced a light-sheet-based multispectral imaging system, which used sequential excitations of differing wavelengths in combination with camera-based readout^10^. This system obviates the first two problems as all the emission light is collected on the same detector and an entire plane is illuminated at once. However, the need to sequentially excite the sample with different wavelengths reduces imaging speed by a factor at least as large as the number of channels. In addition, the spectral unmixing was carried out using the standard linear matrix inversion approach (LU)^11^, requiring that high signal-to-noise ratio (SNR) data must be acquired for accurate results and hence further increasing the illumination dose. Overall, the system was capable of imaging a full cell in six channels every 9.2 s for 100 timepoints.

In this Article, we present an optimized approach for simultaneous multispectral acquisition and subsequent offline unmixing of fluorescence microscopy data that is ideally suited for imaging live samples with minimal phototoxicity. First, we establish an iterative spectral unmixing algorithm and show how its improved performance over the state of the art allows the successful unmixing of low-SNR live-cell data. We then describe a cost-effective hardware module that provides any camera-based fluorescence microscope with eight-channel multispectral imaging capabilities with no penalty in spatiotemporal resolution and with 100% photon efficiency. We then show how these two developments can be combined to achieve multispectral imaging of live cells in 2D+*t* and 3D+*t* on both spinning-disk and light-sheet microscopes, providing full cell volumes in as little as 0.3 s or full cell projections in as little as 0.1 s. Finally, we capitalize on the combination of our imaging technology with de novo-designed protein binders to directly observe endosomal sorting of endogenous transmembrane receptors in living cells.

## Results

### Spectral unmixing of low-SNR data

**Fig. 1:**
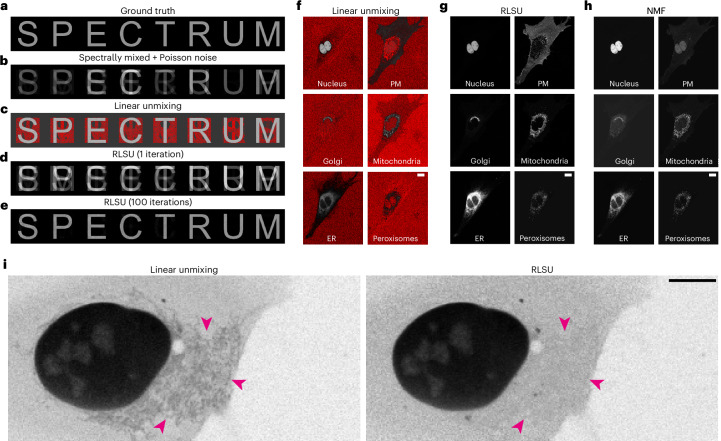
RLSU outperforms linear unmixing and non-negative matrix factorization. **a**, Simulated ground-truth data of eight objects (letters of the word SPECTRUM) labelled with different fluorophores. **b**, Simulated acquired data, spectrally mixed and including Poisson (shot) noise. **c**, Reconstructed objects using data from **b** and linear unmixing. Negative values are shown in red. **d**, Reconstructed objects using data from **b** and one iteration of RLSU. At this stage, the algorithm has not converged on a solution. **e**, Reconstructed objects after convergence using data from **b** and 100 iterations of RLSU. **f**, Linearly unmixed objects from an experimentally acquired dataset of a U2OS cell co-expressing six different fluorescent protein species. Negative values are shown in red. **g**, RLSU unmixed objects from the dataset in **f**. **h**, Non-negative matrix factorization (NMF)-unmixed objects from the dataset in **f**. Note that signals have not been correctly assigned to objects (for example, the nuclear signal in the plasma membrane channel). **i**, Comparison of linear unmixing and RLSU for another real dataset in which mitochondrial signal (magenta arrowheads) bleeds through to the nuclear channel after linear unmixing, but is correctly absent after RLSU. Scale bars, 10 μm.

We first investigated whether traditional spectral unmixing approaches were suitable for application to live-cell imaging datasets. We simulated multispectral datasets by computationally mixing fluorescence from eight fluorophores into eight spectral channels (Fig. 1a) and adding Poisson (that is, shot) noise (Fig. 1b). Unmixing these data using conventional linear unmixing (that is, applying the inverse of the mixing matrix to the mixed signals) produced poor results (Fig. 1c), with more errors associated with unmixing lower-SNR data (Supplementary Fig. 1a–d). In particular, linear unmixing predicted negative quantities of targets in many pixels (shown in red), which is physically impossible. This occurs because linear unmixing is incapable of dealing with Poisson (shot) noise, the predominant form of noise present in low-SNR datasets, such as those generated by live-cell imaging.

We reasoned that using an iterative approach, tailored to incorporate the effects of Poisson (shot) noise, would provide superior results. In particular, we elected to repurpose the Richardson–Lucy algorithm^12,13^ as this is fast, straightforward to use, and has been well-studied over the five decades since its development. Although the Richardson–Lucy algorithm has previously been used for deconvolving images using a known point spread function (PSF), the algorithm is, in principle, applicable to any system where the measurement process can be described by a linear operator and the noise is described by Poisson statistics. Various researchers have used Richardson–Lucy deconvolution to enhance the linewidths of spectral profiles, but these algorithms do not separate the different components as required for spectral unmixing^14–16^. Instead, here we replace the action of convolving with the PSF with the action of mixing with the mixing matrix, which describes the contribution of each underlying object to each channel (Supplementary Note 1 presents more details). Hence, this new spectral unmixing algorithm does not deconvolve the data and so does not suffer from the high-spatial-frequency artefacts often seen after deconvolution. However, our approach can be extended to both unmix and deconvolve the data simultaneously (Supplementary Note 2 and Supplementary Fig. 2) though, for simplicity, we do not use this in this Article.

Applying this Richardson–Lucy spectral unmixing algorithm (RLSU) to the same simulated dataset shows that although one iteration is insufficient to accurately unmix the different components (Fig. 1d), 100 iterations produces a much higher-quality result than linear unmixing (Fig. 1e), even when negative values in the linearly unmixed results are set to zero (Supplementary Fig. 3). In our hands, RLSU also outperformed non-negative least-squares unmixing (Supplementary Note 3 and Supplementary Figs. 4–6) and a recent implementation of phasor-based unmixing, HyU^9^, for both eight-channel (Supplementary Fig. 7) and 32-channel (Supplementary Fig. 8) data. Notably, no negative quantities are generated by RLSU—indeed, this is impossible if the initial estimate of the components is everywhere positive, as the iterative update is multiplicative in nature. We discuss the appropriate Cramér–Rao lower bound in Supplementary Note 4 and show, via simulations (shown in Supplementary Fig. 9), that we reach it. Repeating this process for different SNR levels, and incorporating both Poisson (shot) noise and read noise, we found our iterative algorithm always outperformed linear unmixing (Supplementary Figs. 1, 10 and 11 and Supplementary Video 1). Importantly, read noise only distorts the quality of unmixing results when the variance of the read noise is comparable to the variance of the Poisson (shot) noise. For modern sensors for which the read noise is small, such as the 2.32 *e*^−^ read noise of the cameras used in this work, this means that the effects of read noise are only important for signal levels below five counts. We also found that RLSU outperforms linear unmixing for highly overlapping spectral signals, such as enhanced green fluorescent protein (eGFP) and enhanced yellow fluorescent protein (EYFP) (20-nm peak-to-peak separation), and can unmix signals with a peak-to-peak spectral separation as low as 4 nm using only two channels (Supplementary Note 5 and Supplementary Fig. 12).

Encouraged by these results, we applied our algorithm to data acquired on a commercially available multispectral confocal microscope (Zeiss LSM 710 with 32-channel QUASAR detector). U2OS cells transfected with a polycistronic plasmid (ColorfulCell^17^) encoding six fluorescent protein species targeted to the nucleus (TagBFP), plasma membrane (Cerulean), mitochondria (mAzamiGreen), Golgi apparatus (Citrine), endoplasmic reticulum (mCherry) or peroxisomes (iRFP670) were imaged live, with data exhibiting SNRs up to 13 (Supplementary Fig. 13). We then attempted to unmix the live-cell data using both RLSU and linear unmixing.

Linear unmixing did not always accurately reassign signals to the correct labelled structures, such as the Golgi signal incorrectly present in the mitochondria channel in Fig. 1f, and once again produced numerous pixels with unphysical negative values. In contrast, RLSU produced unmixed objects that accurately resembled the single-labelled controls (Fig. 1g), with no observable bleedthrough or misassignment of signals. A comparison with an implementation of non-negative matrix factorization (NMF), a blind spectral unmixing approach also designed to handle Poisson (shot) noise but without requiring a priori knowledge of the mixing matrix^18^, showed that although this did not produce negative quantities, none of the signals were correctly assigned (Fig. 1h). Most strikingly, substantial nuclear signal was present in the plasma membrane channel. Furthermore, despite being initialized with the correct mixing matrix, the algorithm selected an erroneous mixing matrix as its preferred solution (Supplementary Fig. 14).

Applying both linear unmixing and RLSU on various cell images of differing signal levels, we found that in all cases RLSU was more robust against channel misassignment. Figure 1i shows an example of this, where a weak mitochondrial signal has been assigned to the nuclear channel by linear unmixing, but is correctly absent in the RLSU results. In both cases, the weak background signal outside the nucleus is due to cytosolic fluorescent proteins that have not yet been redirected to the nuclear compartment by the fused targeting signal. Furthermore, we tested the sensitivity of RLSU to small errors in the mixing matrix by unmixing simulated ColorfulCell data with a mixing matrix formed by an incorrect combination of fluorophores (Supplementary Fig. 15). Through this, we found we could, for example, replace mCherry’s mixing matrix values with those for Alexa Fluor 594 with no penalty in the quality of the unmixing results, despite their slightly different spectral profiles. However, mScarlet’s more shifted spectrum led to noticeable reconstruction errors when the mixing matrix was updated to use its values.

### Acquiring video-rate spectral information

Given the performance of RLSU on low-SNR data, we sought to develop imaging hardware that would allow us to capture the necessary raw signals at rates much faster than those afforded by the point-scanning QUASAR detector. In particular, we decided that the hardware should have five key characteristics and should:

- be compatible with any camera-based microscope, by replacing the native camera;
- enable multispectral data capture at the original rate of the parent instrument, thereby maintaining temporal resolution;
- be diffraction-limited, thereby maintaining the spatial resolution of the parent instrument;
- be maximally photon-efficient, thereby maximizing SNR; and
- be easily reconfigurable to best match the spectral characters of the fluorophores used in an experiment.

**Fig. 2:**
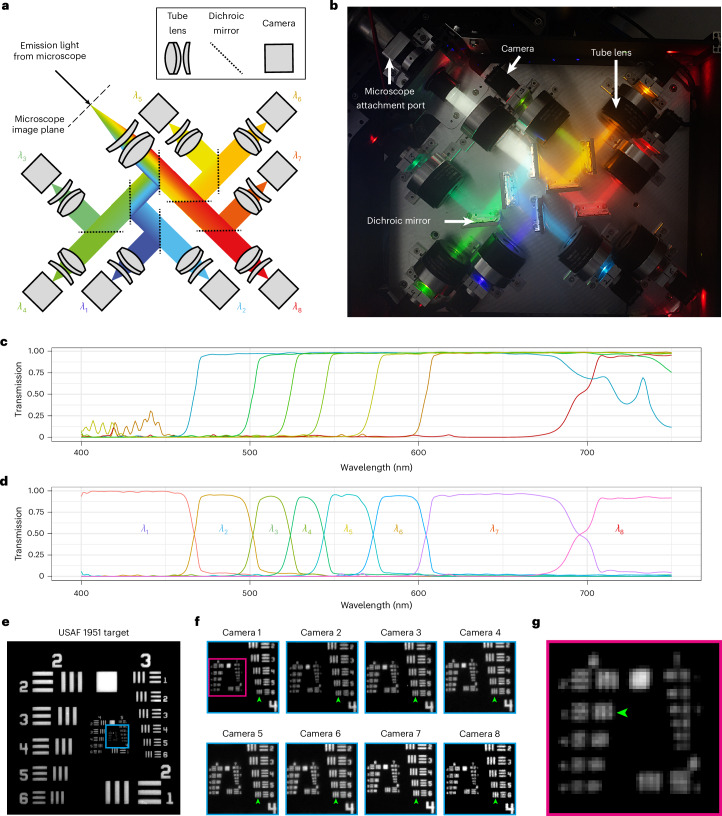
Multispectral acquisition module compatible with camera-based fluorescence microscopes. **a**, Optical path illustration for the multispectral imaging module. Light enters from the top left and is redirected, based on wavelength, onto eight cameras by seven dichroic mirrors. **b**, Photograph of the assembled multispectral imaging module. **c**, Transmission spectra of the seven dichroic mirrors used. **d**, Spectra of the eight channels arising from the spectra in **c**. **e**, USAF 1951 target imaged in the most blue channel. **f**, Enlarged views of the target region indicated by cyan box in **e**, shown for all eight channels. Green arrowheads indicate the finest resolvable group across all channels. **g**, Enlarged view of the target region indicated by the magenta box in **f**, shown for channel 1 (the green arrowhead indicates finest resolvable group in channel 1).

These considerations led us to design a system using dichroic mirrors, in a tree-like arrangement, to redirect light to multiple cameras (see the optical path illustration in Fig. 2a and hardware photograph in Fig. 2b). In contrast to conventional multispectral approaches using diffraction gratings or interferometers, this enables the capture of the full spatial and spectral information in a single shot. Furthermore, as light is merely redirected by the dichroic mirrors, and not absorbed as by a filter, there is no loss of photons through the tree-like arrangement—a photon that does not end up in a specified channel must end up in one of the others. Although this does not obviate the absorbative losses in the extra lenses used, these are small (~3%). Choosing such an approach also means that such a system could image *n* channels in less than half the time that a conventional filter switching system could image in just two, avoiding colour drift (Supplementary Fig. 16).

We settled on using seven dichroics (spectra plotted in Fig. 2c) to provide eight channels of data (spectra plotted in Fig. 2d), as this was the most effective choice for imaging over the typical 450–700-nm spectral range used in live-cell fluorescence microscopy experiments (Supplementary Note 6). In the interests of portability, rejection of excitation light is achieved using the primary dichroic, and if necessary a notch filter, in the parent instrument. Although this will affect the overall spectral profile of the detection unit, this is easily accounted for in the mixing matrix as long as the spectral profile of the dichroic/filter is known.

Raytracing showed that our design would maintain diffraction-limited resolution over the full field of view of the tube lenses used (20-mm diameter), even on the path where the light traverses three tilted dichroic mirrors (Supplementary Fig. 17). To enable future extension to 16 channels, extending the spectral range into the near infrared, we also validated that diffraction-limited resolution was maintained in the case that four tilted dichroics were used. Testing the resolution of the assembled system using a USAF 1951 target (Fig. 2e) showed that the resolving power of the system was limited by the Nyquist sampling rate (6.21 μm pixel^−1^; Supplementary Note 7). This sampling rate was chosen to match that of a typical scientific complementary metal–oxide semiconductor (sCMOS) camera, as commonly used with high-resolution fluorescence microscopes (6.5 μm pixel^−1^).

By design, the sampling rate of the system can be changed by altering the focal length ratio of the input and output lenses. As constructed, we used an input lens with 180-mm focal length, and output lenses with focal lengths of 100 mm. Alternative tube lenses with focal lengths from *f* = 165 mm to 600 mm are also available, giving effective pixel sizes spanning 3.45–20.7 μm. Further adjustments to the effective pixel size would either require replacing the output lenses or selecting different camera sensors.

To ensure maximum mechanical stability, we elected to not mount the dichroic mirrors kinematically. As a result, each image is slightly displaced off-axis owing to small angular deviations of the dichroic mirror orientation from the design specification. However, these shifts were sufficiently small that they could be corrected by a simple image registration routine and left >80% of the field of view usable (Supplementary Fig. 18).

### Multispectral spinning-disk confocal microscopy

**Fig. 3:**
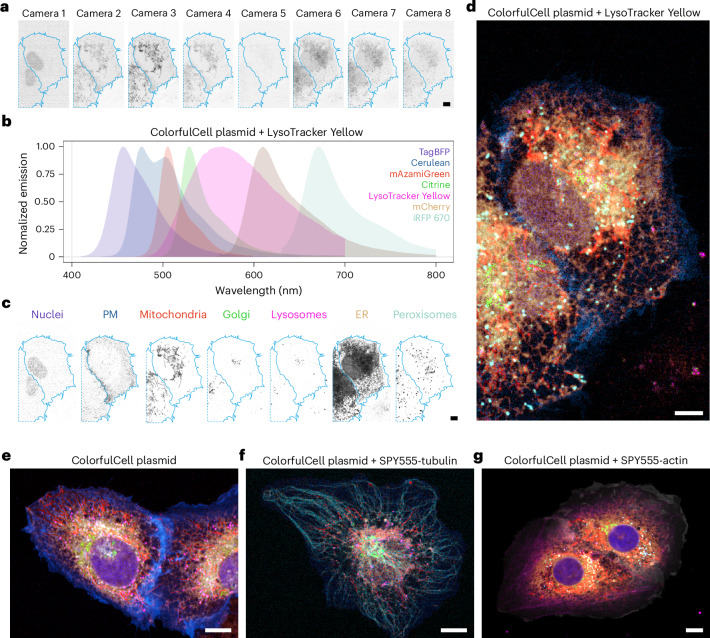
Multispectral live-cell imaging on an SDCM. **a**–**d**, U2OS cells transfected with the ColorfulCell plasmid were incubated with LysoTracker Yellow and imaged live on an SDCM equipped with the multispectral camera system. The eight individual camera views (**a**) were acquired simultaneously, then processed by RLSU unmixing using a mixing matrix generated from the emission spectra of seven fluorophores (**b**) to generate unmixed images for the seven fluorophores (**c**). Colour merge of the channels in **c** is shown in **d**. **e**–**g**, Other examples of RLSU unmixing results for multispectral spinning-disk confocal imaging of U2OS cells transfected with ColorfulCell plasmid (**e**), or additionally incubated with SPY555-tubulin (**f**) or SPY555-actin (**g**) dyes. Scale bars, 10 μm. ER, endoplasmic reticulum; PM, plasma membrane.

First, we coupled our multispectral module to a spinning-disk confocal microscope (SDCM). PSF measurements showed resolutions equivalent to those obtained without the multispectral module, demonstrating that the module did not compromise spatial resolution (Supplementary Fig. 19). Imaging U2OS cells transfected with the six-colour ColorfulCell plasmid, and stained with LysoTracker Yellow, produced eight camera images spanning the visible spectrum (Fig. 3a). To facilitate the choice of appropriate fluorophores and generation of the corresponding mixing matrix, we developed a web application, hosted at beryl.mrc-lmb.cam.ac.uk/calculators/spectral_unmixing/. This uses manufacturer-supplied spectra for the dichroics used in the module, as well as the dichroic and notch filter in the SDCM, in addition to reference fluorescence emission spectra from fpbase.org, to enable the calculation of appropriate mixing matrices and provide feedback on whether the experiment is spectrally feasible (Supplementary Fig. 20). Using a mixing matrix generated by the web application from an appropriate selection of fluorophores (Fig. 3b), we unmixed the camera images to form seven object channels (Fig. 3c).

Despite the fourfold smaller number of spectral channels compared to the QUASAR detector, we obtained unmixed images that accurately reflected the expected distributions of fluorescent proteins in their respective organelles (Supplementary Fig. 21). As the full spatial and spectral information was acquired in one exposure time, we found that imaging speeds could be increased more than 100-fold over our previous confocal QUASAR point-scanning (Supplementary Video 2). Furthermore, the flexibility of the web application interface enabled us to swap LysoTracker Yellow for other live-cell imaging dyes and recompute mixing matrices. These datasets also unmixed accurately, without the need for control measurements of singly-labelled cells to form the mixing matrix (Fig. 3e–g and Supplementary Videos 3–5). Applying LU to these datasets produced results with similar unmixing errors as seen in the previous QUASAR datasets (Supplementary Fig. 22).

Attempts to extend our imaging from 2D+*t* to 3D+*t* were challenged by a noticeable level of phototoxicity after only a few timepoints (Supplementary Fig. 23 and Supplementary Video 6), as expected for fast live-cell spinning-disk confocal microscopy. Although this could be partially mitigated by adding a recovery interval between timepoints, this compromised the imaging speed. In practice, we found that the maximum achievable volumetric imaging rate without substantial photodamage was typically of the order of one volume per minute.

### Multispectral volumetric and projection light-sheet microscopy

**Fig. 4:**
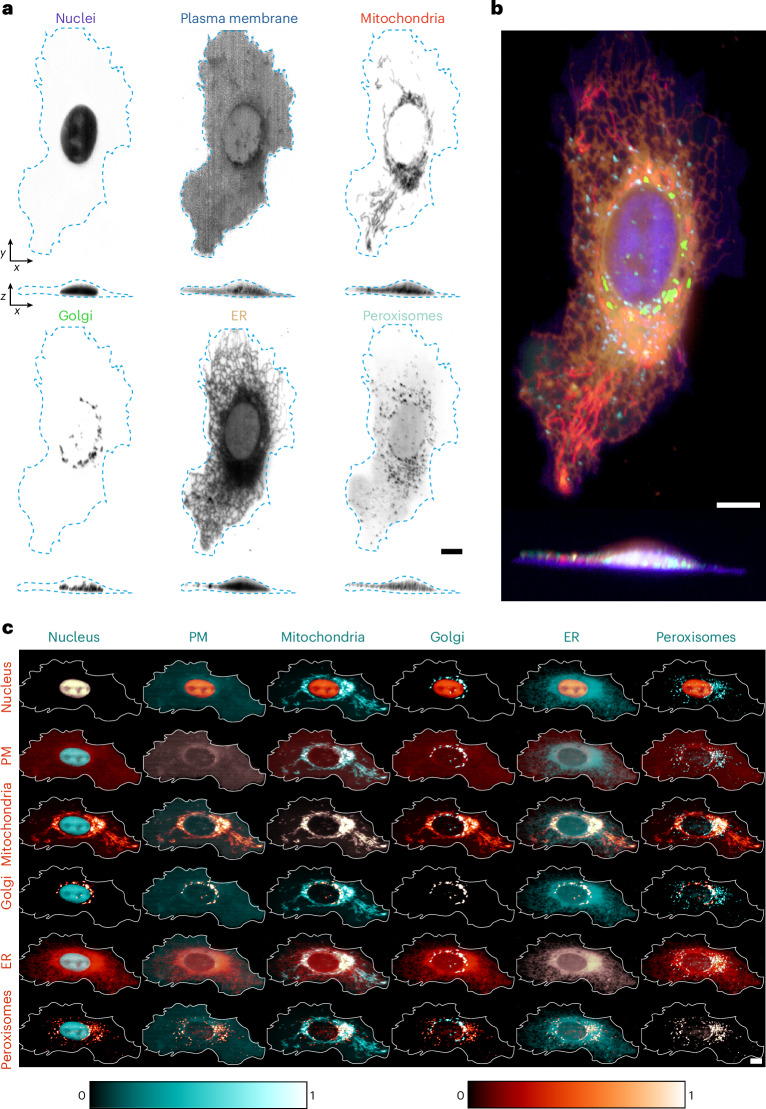
Multispectral live-cell volumetric imaging on an OPM. **a**, U2OS cells transfected with the ColorfulCell plasmid were imaged live by multispectral oblique-plane light-sheet microscopy followed by RLSU unmixing. Top–down and orthogonal-view maximum intensity projections are shown (cell boundaries are in cyan). **b**, Colour merge of the individual channels shown in **a**. **c**, Pairwise montage of the normalized channels shown in **a** (cell boundaries in white). Scale bars, 10 μm.

To reduce photodamage and increase the volumetric acquisition speed, we next coupled our multispectral acquisition module to an oblique-plane light-sheet microscope (OPM)^19,20^. PSF measurements showed that the module again did not compromise the resolution of the instrument, which was comparable to the resolution achieved with the SDCM (Supplementary Fig. 24). As before, the raw signals are correctly unmixed by RLSU into the expected six cellular compartments labelled by the ColorfulCell plasmid (Fig. 4a and Supplementary Videos 7 and 8). As expected, the gentler illumination strategy used by the multispectral OPM allowed us to routinely perform simultaneous six-colour volumetric imaging of cells for at least 200 timepoints, representing a more than tenfold improvement compared to SDCM imaging (Supplementary Video 9).

Although the colour merge, shown in Fig. 4b, provides a good overview of the cell, we found that it was often hard to discern the interactions between components with such high-dimensional datasets. To combat this, we used a ‘pairwise montage’ visualization (Fig. 4c) that shows two components at a time. Each *n* of the *N* objects is assigned a first colour and repeated *N* times down the *n*th column, then assigned a second colour and repeated *N* times across the *n*th row. In this way, the leading diagonal of the visualization shows the overlap of one object with itself (that is, the object itself is displayed in the colour obtained by adding the first colour to the second), while other elements show the overlap of one object with the others. As the object combinations are symmetric about the diagonal, part of the visualization could be excluded as it conveys the same information as is available elsewhere. However, in practice, we found that it was helpful to see both orderings of the colours.

To achieve even faster multispectral imaging speeds, we utilized the recently described shear-warp angled projection technique^21^. By adding a galvo-based image shifting unit in front of the multispectral module and synchronizing the sweep of the light sheet, focal plane and image shift, we could acquire projection images of an entire cell from arbitrary viewing angles in one camera exposure.

**Fig. 5:**
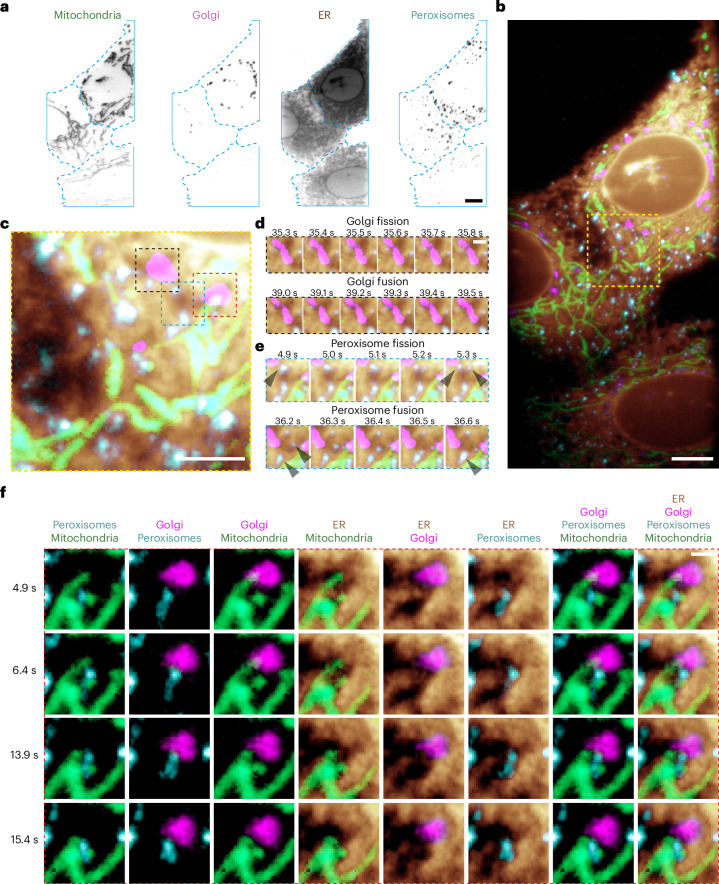
Multispectral live-cell projection imaging on an OPM. **a**, U2OS cells transfected with the ColorfulCell plasmid were imaged by multispectral light-sheet projection imaging followed by RLSU umixing (cell boundaries in cyan). Four channels of a single frame from a video are shown (Supplementary Video 10). **b**, Colour merge of the channels shown in **a**. **c**, Enlarged image of the yellow box in **b**. **d**, Later frames showing Golgi fission, then fusion, in the black box in **c**. **e**, Frames from the cyan box in **c** showing peroxisome fission/fusion. Black arrowheads indicate peroxisomes that undergo fission or fusion within the frames shown. **f**, Frames displaying two-way, three-way and four-way dynamic organelle interactions within the region delineated by the red box in **c**. Scale bars, 10 μm (**a**–**c**) and 2 μm (**d**–**f**).

This combination achieves whole-cell multispectral projections at 10 Hz (Fig. 5a,b and Supplementary Video 10). The laser powers were increased from those used for volumetric imaging to maintain signal levels, but we could not achieve sufficient irradiances for the 405-nm laser to provide acceptable signals from the TagBFP and Cerulean labels. As such, we elected to deactivate this laser to reduce any potential photodamage but continued to unmix data from cells transfected with the ColorfulCell plasmid with a six-component mixing matrix. After unmixing, both the TagBFP and Cerulean channels correctly contained no signal.

The increased acquisition rate provided by the multispectral projection imaging allowed us to see both fast subsecond organelle dynamics for hundreds of timepoints (for example, ER remodelling), but also slower dynamics within the same video. For instance, as can be seen in Fig. 5d, part of the Golgi apparatus fissions in 0.5 s and then, 4 s later, re-fuses. Similarly, Fig. 5e shows fission and fusion events for a number of peroxisomes on a similar timescale, and Fig. 5f shows examples of two-, three- and four-way organelle interactions extracted from the same dataset.

It is worth emphasizing that we have focused in the above on complex samples with a high number (six or seven) of fluorescent probes, but multispectral imaging with our approach does not compromise the imaging quality or speed for simpler samples with just, say, two fluorophores. In fact, multispectral imaging is more photon-efficient than conventional bandpass-filter imaging systems, as light is merely redirected rather than rejected. As such, although we could use a subset of our cameras corresponding to the main peaks of the fluorescence emission spectra to capture data, it is better to use all cameras all the time to enable maximally efficient photon reassignment.

To illustrate this point, we performed two-colour light-sheet imaging of macropinocytic cup closure in a *Dictyostelium* strain expressing LifeAct-mCherry and eGFP fused to a phosphatidylinositol (3,4,5)-trisphosphate (PIP_3_) reporter (Supplementary Fig. 25). This is a particularly challenging sample, as *Dictyostelium* are known to be particularly light-sensitive, and macropinocytic cup closure occurs rapidly in three dimensions, requiring fast volumetric imaging. After careful optimization of exposure times and laser powers, we could reliably acquire videos containing hundreds of timepoints without cell death, in line with our previous experience in imaging this strain on both our non-multispectral original OPM system and a field synthesis light-sheet microscope^22^. In particular, we achieved full cell volumes at 2 Hz, which was enough to follow the process of macropinocytic cup closure and, by relying on simultaneous multispectral acquisition rather than sequential acquisition, the images were devoid of motion blur or colour misregistration (Supplementary Video 11).

### Imaging the dynamics of receptor sorting using de novo-designed receptor minibinders

Fluorescent protein fusions provide fluorescence images free of non-specific binding, but tagging at endogenous levels requires time-consuming genome editing, a process that must be repeated for each colour/target used. As a proof-of-concept experiment, we instead used de novo-designed protein-binding proteins (minibinders), labelled with small-molecule dyes, to visualize the endosomal sorting of endogenous cell-surface receptors.

Endosomal sorting is the process by which cells sort different transmembrane receptors towards three major routes following their endocytosis: degradation in lysosomes, recycling back to the plasma membrane, or retrograde transport to the Golgi apparatus^23^. Imaging endosomal sorting is difficult, first because endosomes move rapidly in three dimensions and second because overexpression of fluorescently labelled receptors can be detrimental. Recently, we developed pipelines to computationally design specific binders for proteins of interest^24,25^. We therefore thought to fluorescently label minibinders, expressed and purified from *Escherichia coli*, that recognize the extracellular region of endogenous transmembrane receptors, and use them to reveal the dynamics of receptor trafficking and sorting in cells (Supplementary Fig. 26a). These de novo-designed binders offer five advantages for multiplexed labelling of endogenous transmembrane receptors when compared to antibodies or fluorescent protein fusions introduced through genome editing:

- they are small (~80–100 amino-acid residues) and so are less likely to perturb trafficking;
- they are designed to express in, and purify from, bacteria well, facilitating access to the reagent;
- they can be specifically labelled with bright organic dyes in a way that does not affect their target-binding ability, as opposed to non-specific labelling for antibodies;
- they are designed to be monovalent and so do not induce receptor clustering;
- they can be designed to be pH-independent, so as not to detach from their target in the acidic environment of late endosomes.

**Fig. 6:**
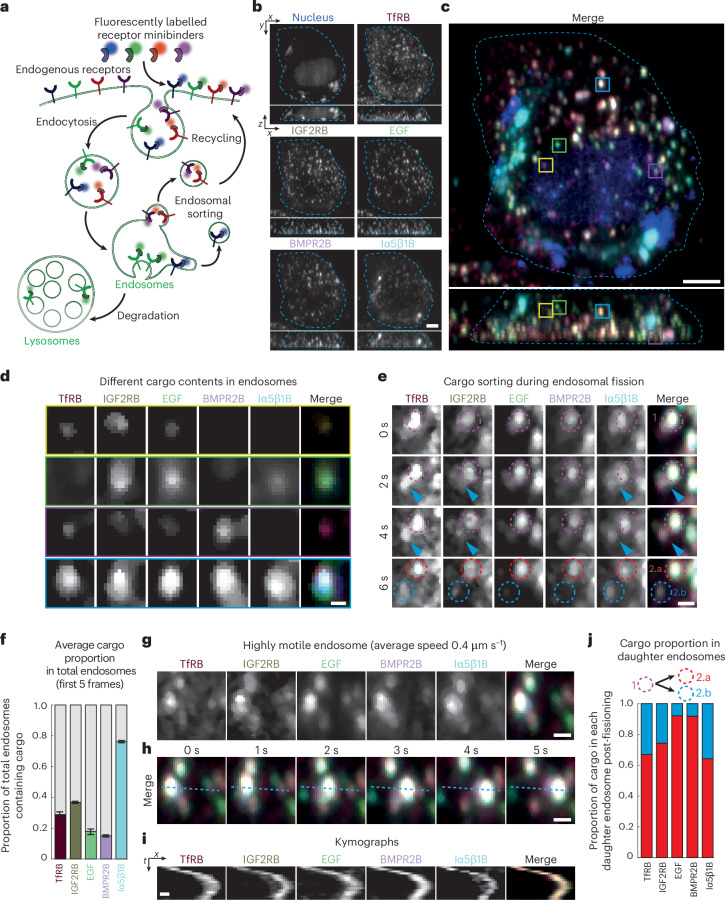
Multispectral light-sheet imaging of intracellular trafficking using computationally designed receptor binders. **a**, Schematic depicting the use of fluorescently labelled, computationally designed receptor binders for imaging receptor trafficking. **b**, Single frame of the RLSU unmixed results (Supplementary Video 13) of a TagBFP-NLS-expressing HeLa Kyoto cell loaded with four fluorescently labelled binders (TfRB, IGF2RB, BMPR2B and Iα5β1B) and one fluorescently labelled receptor ligand (EGF). Top–down and orthogonal-view maximum intensity projections are shown (cell boundaries in cyan). **c**, Colour merge of the channels in **b**. **d**, Individual channels and colour merge of the endosomes indicated by yellow, green, magenta and cyan boxes in **c**. **e**, Individual channels and colour merge of a single endosome during cargo sorting. Frames from Supplementary Video 13 displaying fissioning of this compartment are shown. Magenta dashed circles indicate the parent endosome (labelled ‘1’). Cyan arrowheads indicate the elongation of an intermediate tubule. Red and cyan circles indicate the two daughter endosomes post-fissioning (labelled ‘2.a’ and ‘2.b’, respectively). **f**, Graph depicting the average proportion of total endosomes (*n* = 1,872) containing each cargo across the first five frames of Supplementary Video 13. Error bars denote s.d. **g**, Individual channels and colour merge of a highly motile endosome at *t* = 0. **h**, Colour merge of the endosome in **g** undergoing directional transport. **i**, Kymographs of the highly motile endosome shown in **g** and **h**. **j**, Graph depicting the cargo proportion transferred from the the parent endosome (magenta dashed circle, endosome 1) to each daughter endosome post-fissioning (red and cyan dashed circles, endosomes 2.a and 2.b, respectively) in **e**. Scale bars, 5 μm (**b**,**c**); 1 μm (**d**,**e**,**g**,**h**); 0.5 μm and 2.5 s (**i**).

As a proof-of-concept experiment, we fluorescently labelled four minibinders targeting the transferrin receptor (TfRB), the insulin-like growth factor 2 receptor (IGF2RB), the bone morphogenetic protein receptor type 2 (BMPR2B) and integrin α5β1 receptors (Iα5β1B; Methods and Supplementary Fig. 26b present the minibinder design and Supplementary Fig. 26c biochemical characterization). These four minibinders were labelled with a combination of fluorophores that we had previously validated could be unmixed when imaged using the multispectral OPM (Supplementary Table 3 and Supplementary Fig. 26). When incubated individually with cells, fluorescent minibinders internalize into punctate dynamic structures (Supplementary Fig. 27a; also Supplementary Fig. 27b to show that minibinders are unlikely to be internalized by fluid-phase endocytosis). These structures were reminiscent of endosomes, as confirmed by their colocalization with WASH, an early/sorting endosome marker^26^ (Supplementary Fig. 27c,d and Supplementary Video 12). We thus incubated U2OS cells expressing nuclear TagBFP with all four binders simultaneously alongside labelled epidermal growth factor (EGF) and imaged them with the multispectral OPM (Fig. 6b,c).

Over time, binders were internalized and appeared in highly motile, diffraction-limited objects, as expected for receptors trafficking within the endocytic pathway (Supplementary Video 13). Importantly, the combination of the multispectral imaging module and the rapid imaging afforded by the OPM allowed us to achieve a volumetric imaging rate of 3 Hz in all channels (Supplementary Video 14). This not only allowed us to track all endosomes in the cell in 3D without motion blur, but also with the absence of colour misregistration caused by delays in acquiring colour channels in typical sequential schemes (Fig. 6g–i).

This allowed us to determine the content of each endosome, which revealed that individual compartments showed markedly different distributions of the different minibinders, and thus of their cognate receptors (Fig. 6d, quantified in 6f). For example, the cyan-boxed compartment in Fig. 6c,d contains all labelled receptors and EGF, whereas the yellow-boxed compartment lacks integrin α5β1 and the BMP receptor. Such a selective enrichment of specific receptors in specific endosomes is expected, as we selected receptors known to traffic via different routes, and they would thus tend to be sorted away from each other (Supplementary Fig. 26a).

The high spatiotemporal resolution of the multispectral OPM coupled with the increased brightness conferred by the fluorescent minibinders allowed us to directly image the flow of the different receptors within the endomembrane system. For instance, Fig. 6e shows an endosome containing all labelled binders, plus EGF. Within a few seconds, this compartment elongates a tubule and fissions into two daughter endosomes, with one daughter endosome lacking EGF and BMPR2 (Fig. 6j presents quantification). Hence, during this fission event, the EGF and the BMP receptor binders have been sorted away from the integrin, IGF2 and transferrin receptor binders. Conversely, we could image fusion events during which the content of organelles exchange receptors (Supplementary Fig. 28).

## Discussion

In summary, we have developed an iterative spectral unmixing algorithm capable of handling low-SNR datasets along with camera-based multichannel acquisition hardware. Together, these developments enable fast multispectral microscopy on any camera-based microscope, such as the spinning-disk confocal and oblique-plane microscopes used here. The diffraction-limited hardware design ensures spatiotemporal resolution is uncompromised and, in the future, could be trivially extended to an increased number of channels. Currently, the post-acquisition spectral unmixing code takes longer to process a frame than the acquisition hardware takes to acquire it (~1 s versus 10–250 ms). In the future, this speed could be improved using algorithmic acceleration, such as the approach described in ref. ^27^.

The cameras we chose are inherently less sensitive than typical cameras used for scientific imaging (with quantum yields of ~70% versus ~95%), but the fact that light is merely redistributed to a different detector, rather than filtered, offsets this. If further sensitivity is required, an alternative arrangement using two scientific cameras and commercially available four-way image splitters could be used. We did not investigate this possibility here for reasons of cost and the associated reduction in the usable field of view, but are currently developing such a system for single-molecule imaging.

Although we saw no evidence that co-illumination with multiple simultaneous wavelengths led to an increased rate of photobleaching or phototoxicity, multispectral imaging in itself does nothing to reduce these effects. One-colour imaging allows for the use of the most photostable fluorophore available, but multispectral imaging necessarily requires the use of additional, less photostable, alternatives. As such, continued fluorophore improvement, such as the creation of StayGold^28^, and the development of new technologies to decrease photodamage, such as optical triplet-state depletion^29^ and engineered imaging media^30,31^, will be important in the future development of multispectral imaging.

The use of de novo-designed minibinders, as demonstrated here, offers an attractive alternative to genetically encoded fluorophores, as they can be used to label targets at endogenous levels with substantially more photostable small-molecule dyes. Such binders allowed us to study the sorting of endogenous cell-surface receptors without genome editing, an approach that could potentially be applied to patient-derived cells. However, although we foresee fluorescent binders as an upcoming reagent of choice for targets with an extracellular domain, further work is required to enable efficient cytosolic delivery for intracellular targets.

Altogether, the developments described herein enable the study of multiple, simultaneous subcellular interactions at subsecond timescales over minutes or hours. Crucially, this comes with no penalty in spatiotemporal resolution or detection sensitivity. In the future, we envisage extending the spectral range into the near infrared to enable better deep-tissue imaging, and combining the multispectral imaging unit with single-molecule localization microscopy to substantially enhance resolution.

## Methods

### Multispectral imaging unit design and construction

After deciding on an eight-channel system (Supplementary Note 6), we consulted catalogues of commercially available dichroic mirrors to select seven that would span the visible spectrum in roughly equal steps. We selected zt532rdc, zt594rdc, zt670rdc-xxrxt, zt491rdc, zt458rdc, zt514rdc and zt561rdc, all from Chroma Technology. Constructing a system that splits light onto eight separate cameras, rather than eight regions of one camera, seemed simpler and would also give a larger field of view. As such, we investigated machine vision systems that could support eight or more cameras and selected the xiX platform from Ximea. As we desired an effective pixel pitch similar to that of sCMOS cameras (6.5 μm pixel^−1^) and wanted to use off-the-shelf tube lenses for the image relays (to maintain diffraction-limited performance), we selected a combination of the IMX250 imaging chip (3.45 μm pixel^−1^) with an *f*  = 180 mm input lens and *f* = 100 mm output lenses (Ximea MX050MG-SY, Thorlabs TTL180-A, Thorlabs TTL100-A) to give an effective pixel pitch of 6.21 μm pixel^−1^.

With these components selected, we designed a computer-aided design (CAD) model using Solidworks of an eight-way image-splitter comprising a central aluminium alloy base plate to which all dichroics, cameras and output lenses were attached via holders, and a ‘peninsula’ plate to hold the input lens and C-mount thread. With this design, the input lens focal length can be freely changed, with the peninsula swapped for one of the appropriate length to keep the input image plane in the correct location. The spacings between components had previously been checked using Zemax OpticStudio to ensure that diffraction-limited imaging was possible over the entire field of view (Supplementary Fig. 17).

Components were milled in-house using a Haas TM 1E and assembled. To fix dichroics to the holders, we used DOWSIL 730 FS (Dow Corning) as this does not contract while curing (and hence does not warp the dichroic) and can easily be removed with a razor blade if required. Cameras were roughly placed near the focal plane of each output lens using measurements from the CAD model and then finely focused by imaging the bricks of a distant building (~700 m) through a window, having previously calculated that this distance would produce the same image location as imaging at infinity.

The cameras were controlled by Micro-Manager, using a manufacturer-supplied device adaptor. As the ‘Multi Camera’ device adaptor, which allows the use of multiple cameras, only supports four cameras by default, we edited and recompiled this component to increase the limit to eight. Camera synchronization was handled via transistor–transistor logic (TTL) signals and the manufacturer’s synchronization box (xSWITCH, Ximea).

### Multispectral point-scanning confocal microscopy

For multispectral point-scanning confocal microscopy, U2OS cells expressing the ColorfulCell plasmid were imaged live on a commercial laser scanning confocal microscope (Zeiss LSM 710) equipped with a QUASAR detector, a ×63, 1.4-NA oil-immersion objective (Zeiss Plan-Apochromat, ×63/1.4 oil DIC 420782-9900) and a heated enclosure for imaging at 37 °C and 5% CO_2_. The 405-nm (diode 405-30), 488-nm (argon laser, LASOS Lasertechnik, RMC 781 Z1), 561-nm (DPSS 561-10) and 633-nm (HeNe 633) excitation wavelengths were used for simultaneous excitation of the sample. For the ultraviolet (UV) light path, the MBS 405 dichroic mirror was selected to direct the 405-nm excitation light to the sample. For the visible light path, the MBS 488/561/633 dichroic mirror was selected to direct the excitation light from the 488-, 561- and 633-nm lasers to the sample. The resulting emission was imaged on the QUASAR detector (410.5 nm to 694.9 nm in 32 channels, 9.2-nm spectral resolution) by selecting the ‘Lambda’ mode and directing the light to ‘ChS Detector’ (Spectral detector) in the software. Live cells were imaged in 2D or 3D *Z*-stacks (1,912 × 1,912 pixels) with 4× line averaging, in line sequential mode, with a 2.196-μs pixel dwell time resulting in ~18.8 s per plane imaged and a 70.6-nm effective pixel size. To calibrate the gain and hence SNR in Supplementary Fig. 13, we used the single-image gain and offset calibration software developed by Heintzmann et al.^32^.

### Multispectral spinning-disk confocal microscopy

Imaging was performed on a spinning-disk confocal instrument composed of a Zeiss AxioObserver microscope stand equipped with a fast piezo stage (ASI), a ×63, 1.4-NA oil-immersion objective (Zeiss Plan-Apochromat, ×63/1.4 oil DIC 420782-9900) and a spinning-disk confocal unit (CrestOptics X-Light V3). The spinning-disk confocal unit had two camera ports: a Photometrics Prime 95B back-illuminated sCMOS camera was attached to the perpendicular camera port, and the eight-channel multispectral camera unit was attached to the straight-on camera port via a C-mount thread. A motorized mirror determined to which detector emitted fluorescence was sent. The multispectral camera unit was mounted on pedestal posts (Thorlabs) for stability and to ensure that the unit was at the correct height in relation to the optical path height of the spinning-disk confocal unit. Excitation was provided via a multiline, solid-state laser illuminator (89-North LDI-7) with the following wavelengths available: 405, 445, 470, 520, 528, 555 and 637 nm. The entire system was operated by Micro-Manager. The eight cameras of the multispectral camera acquisition module were controlled by the xSWITCH (Ximea), with cameras 1–7 set to general purpose input (GPI) and camera 8 set to general purpose output (GPO). In the control software, the exposure-start trigger signals for cameras 1–7 were set to be synchronized to the rising-edge exposure signal of camera 8 during an acquisition. In this way, the eight cameras acquired data simultaneously. The other hardware components were controlled via a National Instruments DAQ card (NI PCIe-6321), where a 5 V general purpose output (GPO_5V) trigger from the xSWITCH box controlling the cameras was used to trigger the laser lines and piezo stage during acquisitions. In this way the lasers and the piezo stage motion were triggered based on the rising edge of the camera exposure signals. A quad-band dichroic mirror (ZET405/470/555/640rpc-UF1, Chroma), emission filter (ZET405/470/555/640m-OD8) and excitation cleanup filter (ZET405/470/555/640x, Chroma) were used to enable simultaneous four-colour excitation using the 405-, 470-, 555- and 637-nm lasers. The resulting emission from the sample was recorded on the eight cameras of the multispectral acquisition unit described above. The temperature of the sample was kept at 37 °C using a temperature control chamber (microscopeheaters.com) for live-cell imaging.

### Multispectral oblique-plane light-sheet microscopy

Oblique-plane microscopy was performed on a custom-built set-up (Supplementary Fig. 29), which we previously described in ref. ^33^. In brief, fluorescence was collected by a ×100 1.35-NA silicon-immersion objective (Nikon MRD73950) and directed through an *f* = 200 mm tube lens (Thorlabs TTL200-A) and *f* = 70 mm scan lens (Thorlabs CLS-SL) onto a galvanometric mirror (Thorlabs GVS001). This mirror acted to scan the image plane, and light-sheet illumination, through the sample in a telecentric manner. Light reflected off the mirror was then directed through an *f* = 39 mm scan lens (Thorlabs LSM03-VIS), another *f* = 200 mm tube lens (Thorlabs TTL200-A) and a ×40 0.95-NA air-immersion objective (Nikon MRD70470) into the remote focusing volume^34^. A plane tilted at 30° to the optical axis was then imaged by a ×40 1.0-NA solid-immersion objective (AMS-AGY v1, Applied Scientific Instrumentation) and another *f* = 200 mm tube lens (Thorlabs TTL200-A) onto the input plane of the multispectral camera system.

Excitation light from 405-, 488-, 561- and 638-nm lasers (LBX-405-100-CSB-PPA, LBX-488-100-CSB-PPA and LBX-638-100-CSB-PPA, Oxxius & OBIS 561 nm LS 100 mW, Coherent) was made co-linear and coupled into a polarization-maintaining single-mode fibre (PM-S405-XP, Thorlabs). A reflective parabolic collimator (RC12APC-P01, Thorlabs) attached to the other end of the fibre, produced a 12-mm beam (1/*e*^2^ diameter) which was focused by an *f* = 50 mm achromatic cylindrical lens (LJ1695RM-A, Thorlabs) to form a light sheet. The NA of the light sheet could be varied via an adjustable slit (Thorlabs VA100CP) placed before the cylindrical lens. Another adjustable slit (Thorlabs VA100CP) was placed in the focal plane of the cylindrical lens to limit the lateral extent of the light-sheet illumination. After cropping, the light sheet was directed by an *f* = 75 mm achromatic doublet (Thorlabs AC254-75-A-ML) and two kinematically mounted mirrors onto a kinematically mounted mirror located in a relayed pupil plane. The first two mirrors were used to position the light-sheet illumination correctly in the pupil, with appropriate tilt, and the third mirror could be used to scan the light sheet from side to side in sample space, facilitating alignment. The illumination light was then coupled into the main beam path, between the second *f* = 200 mm tube lens and a ×40 0.95-NA air-immersion objective with a quad-band dichroic beamsplitter (Semrock Di03-R405/488/561/635-t3-25×36).

As with the Crest V3 spinning-disk confocal set-up, the microscope was controlled via Micro-manager^35^ and hardware synchronized with a National Instruments DAQ card (NI PCIe-6321), installed in a custom PC. Of note, this PC featured a ×4 NVMe SSD PCIe mounting card (M.2 Xpander-Aero, MSI) to ensure data from all eight cameras could be written to disk without bandwidth limitations, and a 10G fibre network card to allow acquired data to be transferred to long-term storage promptly. Oblique-plane light-sheet microscopy stacks were computationally deskewed using our home-made software suite lsfm_tools, implementing a linear interpolation, which is available from https://github.com/jdmanton/lsfm_tools.

### Single-channel spinning-disk confocal microscopy

For sequential, single-channel imaging of cells (Supplementary Fig. 27b,c), imaging was performed using a spinning-disk confocal instrument composed of a Nikon Ti stand equipped with a perfect focus system, a fast piezo Z-stage (ASI) and a ×60 1.4-NA oil-immersion objective (Plan-Apochromat Lambda), and a spinning-disk head (Yokogawa CSU-X1). Images were recorded with a Photometrics Prime 95B back-illuminated CMOS camera run in pseudo global shutter mode and synchronized with the spinning-disk wheel. Excitation was provided by 488- and 630-nm lasers (Coherent OBIS mounted in a Cairn laser launch) and imaged using dedicated single-bandpass filters for each channel mounted on a Cairn Optospin wheel (Chroma 525/50 for eGFP and Semrock 647lp for Alexa Fluor 647). The temperature was kept at 37 °C using a temperature control chamber (microscopeheaters.com). The system was operated with Metamorph.

### Image registration software

Image registration software was implemented in Python 3 using the tifffile, numpy and imreg_dft modules. Reference images, either of back-illuminated lens tissue or of spinning-disk confocal pinholes, were collected and used to generate affine transformations to register each channel to channel 1 via cross-correlation (examples are provided in Supplementary Fig. 18). Affine transform parameters were saved to disk and later used to register experimental data, before unmixing.

### Spectral unmixing software

Richardson–Lucy spectral unmixing software was developed in Python 3 using the numpy, cupy, tifffile and pandas modules and is available from https://github.com/jdmanton/rlsu. Briefly, registered TIFF stacks are loaded, along with a CSV file representing the mixing matrix for the experiment in question. For each *z* plane of the stack, image data are reshaped into a 2D matrix and the initial RLSU estimate is initialized with a matrix of all ones. Matrix multiplication is carried out on the graphics processing unit. After a user-defined number of iterations (default: 1,000), RLSU is terminated and the RLSU result is reshaped into a hyperstack, which is then saved to disk as a TIFF. When processing oblique-plane light-sheet microscopy data, multispectral images were registered and unmixed first, then deskewed.

### Cell culture

U2OS FlipIn Trex cells and HeLa Kyoto cells (RRID:CVCL_1922) were cultured in DMEM-Glutamax (Gibco) supplemented with 10% fetal bovine serum (FBS, Gibco) and 1% penicillin-streptomycin (Gibco) at 37 °C in 5% CO_2_. 3T3 FlipIn cells stably expressing eGFP WASH^26^ were cultured in DMEM-Glutamax (Gibco) supplemented with 10% donor bovine serum (Gibco) and 1% penicillin-streptomycin (Gibco) at 37 °C and 5% CO_2_. Cells were transfected with Lipofectamine 3000 (Invitrogen) according to the manufacturer’s instructions, and imaged after one day of expression. Cells were regularly screened for mycoplasma using a MycoAlert Mycoplasma Detection Kit (Lonza). On the day of the experiment, transfected (or stable) cells were always plated on glass-bottomed dishes (World Precision Instruments, FD35) coated with fibronectin (Sigma, F1141, 50 μg ml^−1^ in phosphate buffered saline (PBS)), for 2 h at 37 °C in DMEM-10% serum. For imaging, the medium was then changed to pre-warmed, filtered Leibovitz’s L15 medium (Gibco), enriched with 4.5 g l^−1^ glucose, 10% FBS and 20 mM HEPES (Gibco). In some instances, live-cell dyes, such as SPY 555 tubulin, SPY 555 actin (Spirochrome) or LysoTracker Yellow HCK 123 (Thermo Fisher Scientific) were used. Lyophilized live-cell dyes were reconstituted in 50 μl dimethyl sulfoxide to prepare a 1,000× stock solution according to the manufacturer’s instructions. This stock solution was stored at −20 °C. On the day of the experiment, the probe was diluted to a 1× staining solution in full growth medium and vortexed briefly. Cells in imaging dishes were incubated at 37 °C with staining solution for 1 h. The staining solution was then removed, the cells were washed in PBS and warm imaging medium was added for imaging.

### *Dictyostelium* culture and imaging

The *Dictyostelium discoideum* strain HM3478 used in this work is derived from Ax2(Kay)^36^ by transformation^37^ with the plasmid pPI304 (available from Addgene under ID 113232) expressing reporters for PIP_3_ and actin (PH-PkbE-eGFP, LifeAct-mCherry, respectively). Cells were grown at at 22 °C in HL5 medium (Formedium) containing 10 μg ml^−1^ G418. They were harvested in log phase and imaged in simple upregulation medium (SUM) medium, which maintains macropinocytosis but reduces auto-fluorescence, as described previously^38,39^.

### Minibinder design

Full details of the design of the transferrin receptor minibinder are provided in ref. ^24^, and details of the IGF2R minibinder design in ref. ^40^. These were designed using an established Rosetta design pipeline^25^. Manuscripts describing the design of the BMPR2 and integrin α5β1 minibinders are currently in preparation.

### Constructs

The ColorfulCell plasmid used throughout this Article, which encodes six fluorescent protein targeted to different organelles, was described previously in ref. ^17^ and obtained from Addgene. For NLS-TagBFP expression (Fig. 6), a construct encoding a nuclear localization sequence (NLS) followed by TagBFP was synthethized by Twist Bioscience and cloned into a plasmid containing a cytomegalovirus promoter.

For integrin α5β1, IGFR2 and BMPR2 minibinder production, genes encoding the minibinders were optimized for bacterial expression using codon optimization and RNA ddG minimization as described previously^41^. Minibinder sequences were then flanked in 5′ with a FseI restriction side followed by DNA sequence encoding a unique reactive cysteine KKCKK to enable downstream maleimide labelling (next section) and in 3′ by a 10× histidine tag for protein purification followed by an AscI restriction site. The resulting synthetic construct was synthesized by Twist Bioscience, and subcloned into a modified pGEX vector to express a protein of interest downstream of the gluthathione *S*-transferase (GST) purification tag followed by tobacco etch virus and 3C cleavage sequences^33^. Ultimately, this strategy resulted in minibinders flanked by GST-TEV-3C-KKCKK in the N terminus and 10× His in the C terminus (that is, GST-TEV-3C-KKCKK-Minibinder-10×His).

### Minibinder purification and labelling

Full details of minibinder purification and labelling are presented in Supplementary Note 8.

### Quantification of cargo abundances

To determine the proportion of the total endosomes containing each cargo in Supplementary Video 13, commercial image analysis software was used (Imaris, Oxford Instruments). In brief, the five cargo channels were projected in ImageJ to create a pseudochannel corresponding to the total trafficking compartments present in the video. In Imaris, each channel (including the ‘total compartments’ pseudochannel) was segmented in 3D using the ‘Spots’ function, accounting for the anisotropy of the PSF. As the signals in each of the channels were near the diffraction limit, the *xy* and *z* diameters used for segmentation were 0.5 μm and 1 μm, respectively. An object-based colocalization method was carried out for each cargo channel with the ‘total compartments’ pseudochannel in the software with the distance for colocalization classified as being below 0.5 μm. This allows for the proportion of each cargo colocalizing with the total segmented signals to be determined at each timepoint. For normalization, the number of colocalizing signals for each channel was determined as a ratio compared with the total segmented signals. These data were averaged across the first five timeframes of the video (Fig. 6f).

## Online content

Any methods, additional references, Nature Portfolio reporting summaries, source data, extended data, supplementary information, acknowledgements, peer review information; details of author contributions and competing interests; and statements of data and code availability are available at 10.1038/s41566-025-01745-7.

## Supplementary information

Supplementary Information Supplementary Notes 1–8, Supplementary Figs. 1–29

Supplementary Video 1 RLSU outperforms linear unmixing with simulated multispectral datasets

Supplementary Video 2 Multispectral spinning-disk confocal imaging of organelle trafficking

Supplementary Video 3 Multispectral spinning-disk confocal imaging

Supplementary Video 4 Multispectral spinning-disk confocal imaging of microtubules and various organelles

Supplementary Video 5 Multispectral spinning-disk confocal imaging of actin and various organelles

Supplementary Video 6 Volumetric multispectral spinning-disk confocal microscopy exhibits significant photobleaching

Supplementary Video 7 Multispectral volumetric light-sheet imaging

Supplementary Video 8 Multispectral volumetric light-sheet imaging timelapse

Supplementary Video 9 Long-term, volumetric imaging by multispectral oblique-plane light-sheet microscopy

Supplementary Video 10 Fast multispectral live-cell imaging by projection light-sheet microscopy

Supplementary Video 11 Fast volumetric imaging of live *Dictyostelium discoideum* cells

Supplementary Video 12 Validation that de novo-designed binders reach early-sorting endosomes in live cells

Supplementary Video 13 Multispectral live-cell light-sheet imaging of intracellular trafficking using de novo-designed receptor minibinders

Supplementary Video 14 Fast multispectral live-cell light-sheet imaging of intracellular trafficking using de novo-designed receptor minibinders

## Data Availability

Data are available from the corresponding author on request.

## Code availability

Spectral unmixing code is available from https://github.com/jdmanton/rlsu.
