# Supplementary material for: Multispectral live-cell imaging with uncompromised spatiotemporal resolution
Source: Nat Photonics. 2025 Sep 8;19(10):1146–56. doi: 10.1038/s41566-025-01745-7 (PMC12488498; doi:10.1038/s41566-025-01745-7)
Supplement: Supplementary file 1 — Supplementary Notes 1–8, Supplementary Figs. 1–29 [file 41566_2025_1745_MOESM1_ESM.pdf]

nature photonics

Supplementary information

https://doi.org/10.1038/s41566-025-01745-7

Multispectral live-cell imaging with uncompromised spatiotemporal resolution

In the format provided by the authors and unedited

A Richardson–Lucy spectral unmixing

In the Richardson–Lucy algorithm, an estimate of the underlying object, u_t, is updated by the iterative step:

$$u_{t+1} = u_t \cdot \mathrm{H}^{\mathrm{T}} \left(\frac{d}{\mathrm{H} u_t} \right), \tag{S1}$$

where d is the measured (noisy) data, H is the measurement operator and H^{T} is the transpose (dual) of the measurement operator [12, 13]. Both the multiplication and division operate elementwise. For deconvolution applications, H represents a convolution with the point spread function, with H^{T} being a convolution with the flipped (in x, y and z) point spread function.

For Richardson–Lucy spectral unmixing (RLSU), we set H to the mixing matrix that describes the contribution of each underlying object to each channel. For example, consider one object, A, that contributes 5 counts to channel 1 for every 15 counts to channel 2, along with another object, B, that contributes 0 counts to channel 1 (*i.e.* all counts go into channel 2). Then,

$$\mathrm{H} = \begin{bmatrix} 0.25 & 0.00 \\ 0.75 & 1.00 \end{bmatrix}, \tag{S2}$$

and

$$\mathrm{H}^{\mathrm{T}} = \begin{bmatrix} 0.25 & 0.75 \\ 0.00 & 1.00 \end{bmatrix}. \tag{S3}$$

Let us now say that, in an experiment, A emits with a rate of 100 photons/s and B emits with a rate of 200 photons/s. For perfect detection efficiency over an integration time of 1 s, we would then expect:

$$\hat{d} = \begin{bmatrix} 0.25 & 0.00 \\ 0.75 & 1.00 \end{bmatrix} \begin{bmatrix} 100 \\ 200 \end{bmatrix} = \begin{bmatrix} 25 \\ 275 \end{bmatrix}. \tag{S4}$$

However, as we are dealing with counts, Poisson statistics apply and so the elements of d are drawn from Poisson distributions with the elements of $\hat{d}$ as rate parameters. As such, we may in fact measure:

$$d = \begin{bmatrix} 33 \\ 284 \end{bmatrix}. \tag{S5}$$

Traditional spectral unmixing (linear unmixing, LU) would then apply the (pseudo)inverse of H to d, to obtain:

$$\begin{bmatrix} 4 & 0 \\ -3 & 1 \end{bmatrix} \begin{bmatrix} 33 \\ 284 \end{bmatrix} = \begin{bmatrix} 132 \\ 185 \end{bmatrix}. \tag{S6}$$

Hence, after standard linear unmixing, we infer that A emits with a rate of 132 photons/s and B emits with a rate of 185 photons/s.

For RLSU, we begin with an initial estimate:

$$u_0 = \begin{bmatrix} 1 \\ 1 \end{bmatrix}, \tag{S7}$$

which produces predicted data

$$\mathrm{H} u_0 = \begin{bmatrix} 0.25 & 0.00 \\ 0.75 & 1.00 \end{bmatrix} \begin{bmatrix} 1 \\ 1 \end{bmatrix} = \begin{bmatrix} 0.25 \\ 1.75 \end{bmatrix}. \tag{S8}$$

This choice of u_0 is arbitrary, but all elements must be positive definite in order to ensure a positive result. Note that the predicted data are not necessarily integer, even though the real measured data are. The first updated estimate is then given by:

$$u_1 = \begin{bmatrix} 1 \\ 1 \end{bmatrix} \cdot \begin{bmatrix} 0.25 & 0.75 \\ 0.00 & 1.00 \end{bmatrix} \begin{bmatrix} 33/0.25 \\ 284/1.75 \end{bmatrix} = \begin{bmatrix} 154.7143\ldots \\ 162.2857\ldots \end{bmatrix} \tag{S9}$$

Further updates then give:

$$u_2 = \begin{bmatrix} 151.4032 \\ 165.5968 \end{bmatrix}, \quad u_3 = \begin{bmatrix} 148.5256 \\ 168.4744 \end{bmatrix}, \quad u_4 = \begin{bmatrix} 146.0386 \\ 170.9614 \end{bmatrix}, \quad u_5 = \begin{bmatrix} 143.8994 \\ 173.1006 \end{bmatrix}, \quad \ldots \tag{S10}$$

After the 24th iteration, we have

$$u_{24} = \begin{bmatrix} 132.4331 \\ 184.5669 \end{bmatrix}, \tag{S11}$$

which rounds to

$$\hat{u} = \begin{bmatrix} 132 \\ 185 \end{bmatrix}. \tag{S12}$$

Further iterations produce floating point numbers ever closer to 132 and 185, *i.e.* RLSU converges to the same solution as obtained by applying the inverse of the mixing matrix to the measured data (Supplementary Equation S6).

At first glance, this process may seem pointless — we have used many matrix multiplications to obtain the same result as we could easily obtain with just one. However, now consider a modified, more realistic, experiment, for which we have:

$$H = \begin{bmatrix} 0.0882 & 0.0009 & 0.0166 & 0.0081 \\ 0.5002 & 0.0365 & 0.0206 & 0.2662 \\ 0.2697 & 0.2896 & 0.0171 & 0.5238 \\ 0.1419 & 0.6731 & 0.9457 & 0.2019 \end{bmatrix}, \quad \hat{d} = \begin{bmatrix} 11.3798\ldots \\ 82.3518\ldots \\ 110.0174\ldots \\ 196.2509\ldots \end{bmatrix}, \quad d = \begin{bmatrix} 13 \\ 96 \\ 104 \\ 186 \end{bmatrix}, \tag{S13}$$

with each underlying object producing an expected count of 100. Now, RLSU produces an estimate of

$$u_{\mathrm{RLSU}} = \begin{bmatrix} 111.3737 \\ 0.0000 \\ 150.6401 \\ 136.9862 \end{bmatrix}, \tag{S14}$$

while the standard LU approach produces

$$u_{\mathrm{inv}} = \begin{bmatrix} 98.5806 \\ -46.9786 \\ 179.4886 \\ 167.9094 \end{bmatrix}. \tag{S15}$$

As well as producing an unphysical negative number, the LU estimates for the third and fourth component are pushed further away from the ground truth value of 100. Calculating root-mean-square-errors gives 90.2 for LU and 59.3 for RLSU. Setting the second component of u_{inv} to zero and recomputing the RMSE gives 72.3, *i.e.* still higher than that of RLSU. Arguably, the Kullback–Leibler divergence is a more appropriate measure of accuracy, but this cannot be computed for the LU approach due to the negative number present in the estimate.

Together, these (somewhat contrived) demonstrations illustrate the general observation that RLSU produces estimates equivalent to LU when LU predicts all-positive estimates, but produces more accurate estimates when LU predicts estimates containing some negative numbers. Importantly, the RLSU estimates in this case are more accurate than a modified LU estimate where all negative numbers have been zeroed.

B Simultaneous spectral unmixing and deconvolution

As both diffractive blurring and spectral mixing are linear operations (H_{blur} and H_{mix}, respectively), their combined effect is also a linear operator (H_{full}) and hence should be amenable to inversion using the Richardson–Lucy algorithm. To test this, we simulated datasets in which eight ground truth objects corresponding to the letters in the word SPECTRUM (Supplementary Figure 2a) were each blurred with eight different point spread functions (PSFs, with size increasing from bluest channel to reddest channel). These blurred images were then mixed according to a mixing matrix (thereby completing the action of the composite operation) and Poisson (shot) noise added (Supplementary Figure 2b).

To calculate a Richardson–Lucy iteration, we need to know the form of the transpose of the operation, H^T_{full}. Following linear algebra, we know that

$$H^T_{full} = (H_{mix}H_{blur})^T = H^T_{blur}H^T_{mix}, \tag{S16}$$

and so we need to mix with the transpose of the mixing matrix first, and then blur with flipped PSFs. Compared with normal Richardson–Lucy spectral unmixing (RLSU, Supplementary Figure 2c), incorporating deconvolution into the unmixing produces sharper images without compromising the quality of the unmixing (Supplementary Figure 2). We validate that the combined action of unmixing and deconvolving does not lead to errors in signal quantification by comparing the mean value of each ground truth object to those produced by standard linear unmixing, RLSU and the combined deconvolution-unmixing (Supplementary Figure 2). Furthermore, Supplementary Figure 2 shows that the mean squared error between ground truth and unmixed objects is minimised by the deconvolution-unmixing approach. However, a visual inspection shows that the deconvolution-unmixing results suffer from the same kind of high-frequency ‘squiggle’ artefacts that plague normal Richardson–Lucy deconvolution and so we expect that, in the future, better results will be obtained by incorporating some form of explicit or implicit regularisation. It must be emphasised that these artefacts arise from the deconvolution part of the iteration and are not present in the Richardson–Lucy spectral unmixing as used in the rest of the manuscript.

C Comparison of Richardson–Lucy and non-negative least squares spectral unmixing

While our results in the main text show that Richardson–Lucy spectral unmixing (RLSU) outperforms linear unmixing (LU) and linear unmixing with negative values set to zero (LUNN), this is arguably not a fair fight as LU is non-iterative. Myriad non-negative least squares (NLS) algorithms exist, which can also be applied to the problem of spectral unmixing and benefit, similar to RLSU, from the knowledge that the underlying objects cannot have negative values. Here, we investigate the performance of NLS compared with RLSU, using the MATLAB `lsqnonneg` function (in our testing, this produced equivalent results to the Landweber algorithm and the Mathematica `FindMinimum` function).

First, we note that LU acts as a maximum likelihood estimator for the case of Gaussian noise. In contrast, RLSU acts as a maximum likelihood estimator for the case of Poisson noise. At high signal levels, the true Poisson-distributed shot noise is well approximated by a Gaussian noise model and so we should expect both algorithms to give the same answer in these situations. Similarly, NLS should produce identical results to LU in this case (as there is sufficient signal to preclude the prediction of negative values with LU) and so this also achieves the maximum likelihood estimate for Gaussian noise. However, at lower signal levels the Gaussian noise approximation is inaccurate. Furthermore, NLS assumes that the measured data are homoskedastic, i.e. the variance of the measured counts in each channel are equal. This would only be the case in the unlikely situation that all channels see the same number of expected counts, as for a Poisson-distributed signal the variance scales with the signal level (i.e. higher signals have higher variance).

These considerations led us to investigate two cases through simulations in which, for each signal level, we drew 100 times from appropriate Poisson distributions to generate noisy datasets which were processed with both RLSU and NLS. The number of times each method produced a mean squared error less than the other method was recorded, along with the number of ties (i.e. instances where both algorithms produced the same result). This was repeated ten times per signal level, with the mean and standard deviations being shown in Supplementary Figure 4. First, we considered the situation in which all objects emit at the same rate and the mixing matrix is

$$\frac{1}{3}\begin{bmatrix} 2 & 1 & 0 & 0 \\ 1 & 1 & 1 & 0 \\ 0 & 1 & 1 & 1 \\ 0 & 0 & 1 & 2 \end{bmatrix}, \tag{S17}$$

so that we have equal expected counts, and hence variance, in all four channels. The results of the simulation are shown in Supplementary Figure 4a. Clearly, RLSU outperforms NLS at low signal levels (i.e. low expected object photon emission numbers), but at higher signal levels the number of ties is almost 100, i.e. both algorithms perform equally well.

We then considered the situation in which all objects emit at the same rate, but the mixing matrix is

$$\frac{1}{6}\begin{bmatrix}4 & 0 & 0 & 0\\ 2 & 3 & 2 & 0\\ 0 & 3 & 2 & 2\\ 0 & 0 & 2 & 4\end{bmatrix}. \tag{S18}$$

In this situation, rather than expecting signals of $(1,1,1,1)/4$ times the object emission level, we now expect $(4,7,7,6)/24$ times the object emission level, i.e. the total signal is the same, but is now distributed unequally. As the variance of the Poisson noise depends on the expected count in each channel, this mixing matrix produces heteroskedastic data even for equal object emission levels. Our expectation that this leads to worse performance for NLS is confirmed in Supplementary Figure 4b where, now, even at high expected photon emission numbers RLSU outperforms NLS. As RLSU outperforms NLS on homoskedastic, low count data and heteroskedastic data of any count level, we conclude that RLSU is a more appropriate algorithm to use, especially as there is no real speed advantage to using NLS.

D Cramér–Rao lower bound analysis of spectral unmixing

To calculate the Cramér–Rao lower bound for estimating objects given Poisson-noisy mixed signals, we begin with the model for the expected photon counts:

$$\mu_k = \sum_{l=1}^{L} \mathrm{H}_{kl} x_l, \quad k = 1, 2, \ldots, K \tag{S19}$$

for L fluorophore species and K channels. For the sake of simplicity, we assume that $K = L$ and that the mixing matrix, H, is invertible. Furthermore:

$$\mathrm{H}_{kl} = \int d\lambda F_k(\lambda) G_l(\lambda), \tag{S20}$$

with $F_k(\lambda)$ the detection spectrum and $G_l(\lambda)$ the emission spectrum, is assumed to be normalised such that

$$\sum_k \mathrm{H}_{kl} = 1 \quad \text{for all } l. \tag{S21}$$

Then, the total number of expected detected photons satisfies:

$$\sum_k \mu_k = \sum_{k,l} \mathrm{H}_{kl} x_l = \sum_l x_l, \tag{S22}$$

so that in the unmixing the total number of photons is conserved.

The log-likelihood is:

$$\log \mathscr{L} = \sum_k \left[m_k \log \mu_k - \mu_k - \log(m_k!) \right], \tag{S23}$$

with m_k the observed photon counts. These satisfy $\langle m_k \rangle = \mu_k$ and $\langle m_k m_l \rangle = \mu_k \mu_l + \mu_k \delta_{kl}$, according to Poisson statistics. The derivative of $\log \mathscr{L}$ with respect to the estimated x_l is:

$$\frac{\partial \log \mathscr{L}}{\partial x_l} = \sum_k \frac{\partial \log \mathscr{L}}{\partial \mu_k} \frac{\partial \mu_k}{\partial x_l} = \sum_k \left[\frac{m_k}{\mu_k} - 1 \right] \mathrm{H}_{kl} = \sum_k \frac{m_k}{\mu_k} \mathrm{H}_{kl} - 1. \tag{S24}$$

For the Cramér–Rao lower bound we need the Fisher information matrix:

$$F_{ij} = \langle \frac{\partial \log \mathscr{L}}{\partial x_i} \frac{\partial \log \mathscr{L}}{\partial x_j} \rangle = 1 - \sum_k \frac{\langle m_k \rangle}{\mu_k} \mathrm{H}_{ki} - \sum_k \frac{\langle m_k \rangle}{\mu_k} \mathrm{H}_{kj} + \sum_{k,l} \frac{\langle m_k m_l \rangle}{\mu_k \mu_l} \mathrm{H}_{ki} \mathrm{H}_{lj} = \sum_k \frac{1}{\mu_k} \mathrm{H}_{ki} \mathrm{H}_{kj}. \tag{S25}$$

We can write this conveniently in matrix form as:

$$\mathrm{F} = \mathrm{H}^{\mathrm{T}} \mathrm{M}^{-1} \mathrm{H}, \tag{S26}$$

with $M_{kl} = \mu_k \delta_{kl}$ and $\mathrm{M}^{-1}_{kl} = \frac{1}{\mu_k} \delta_{kl}$. Now, the inverse of F follows as:

$$\mathrm{F}^{-1} = \mathrm{H}^{-1} \mathrm{M} (\mathrm{H}^{-1})^{\mathrm{T}}, \tag{S27}$$

and so the Cramér–Rao lower bound is:

$$\langle \Delta x_l^2 \rangle \geq \left[\mathrm{F}^{-1}\right]_{ll} = \sum_k \mu_k \left[(\mathrm{H}^{-1})_{lk} \right]^2. \tag{S28}$$

This is also what one would expect if the estimator is:

$$\hat{x}_l = \sum_k (\mathrm{H}^{-1})_{lk} m_k, \tag{S29}$$

and then, using error propagation:

$$\langle \Delta \hat{x}_l^2 \rangle = \sum_{k,p} (\mathrm{H}^{-1})_{lk} (\mathrm{H}^{-1})_{lp} \langle \Delta m_k \Delta m_l \rangle = \sum_k \mu_k \left[(\mathrm{H}^{-1})_{lk} \right]^2 . \tag{S30}$$

To demonstrate that we approach this Cramér–Rao lower bound, we ran a simulation of eight ground truth objects, each with value 100, mixed using the following mixing matrix (obtained from our web app using spectra for TagBFP, Citrine, mAzamiGreen, Qdot 565, Qdot 585, Qdot 605, Qdot 655 and Qdot 705):

$$\begin{bmatrix} 0.0093 & 0.2376 & 0.0005 & 0.0003 & 0.0000 & 0.0000 & 0.0001 & 0.8259 \\ 0.1681 & 0.4641 & 0.0190 & 0.0030 & 0.0007 & 0.0003 & 0.0010 & 0.0973 \\ 0.3825 & 0.1817 & 0.0682 & 0.0038 & 0.0006 & 0.0003 & 0.0010 & 0.0431 \\ 0.2671 & 0.0908 & 0.6250 & 0.1201 & 0.0322 & 0.0193 & 0.0182 & 0.0213 \\ 0.1180 & 0.0253 & 0.2742 & 0.6957 & 0.4525 & 0.0077 & 0.0016 & 0.0096 \\ 0.0390 & 0.0001 & 0.0090 & 0.1597 & 0.4916 & 0.2084 & 0.0264 & 0.0024 \\ 0.0147 & 0.0003 & 0.0040 & 0.0169 & 0.0217 & 0.7313 & 0.3742 & 0.0003 \\ 0.0013 & 0.0000 & 0.0001 & 0.0004 & 0.0007 & 0.0326 & 0.5774 & 0.0000 \end{bmatrix} \tag{S31}$$

1,000 independent realisations of Poisson (shot) noise were generated and the noisy signals unmixed using RLSU. Supplementary Figure 9 shows the distribution of the reconstructed values, with the means shown as black triangles. These are correctly located around 100, with the experimental standard deviation (shown as a solid errorbar) matching well with the square root of the Cramér–Rao lower bound (shown as a dotted errorbar).

E Effects of decreased spectral separation on unmixing results

As RLSU was developed to reconstruct ground truth objects from spectrally overlapping datasets, we wanted to assess its performance when decreasing the degree of spectral separation between fluorophores. Two channel spectrally-overlapping Poisson (shot) noisy datasets were simulated using ground truth objects 'S' and 'P', the emission spectra of EGFP and EYFP, and an idealised dichroic mirror with an infinitely steep cut-on wavelength equidistant from the spectral peaks of EGFP (510 nm) and EYFP (530 nm). EGFP and EYFP were chosen as they have significant spectral overlap, near identical spectral shape and only 20 nm peak-to-peak spectral separation. As shown in Supplementary Figure 12a, after 100 iterations, RLSU was able to faithfully reconstruct the ground truth objects ($\mathrm{RMSE}_S = 0.289, \mathrm{RMSE}_P = 0.247$) whilst the results from linear unmixing contained many negative pixel values (indicated in red) and erroneous pixel reassignment ($\mathrm{RMSE}_\mathrm{S} = 0.604, \mathrm{RMSE}_\mathrm{P} = 0.636$).

To further test the degree of spectral separation required for accurate reconstruction via RLSU, the EGFP and EYFP emission spectra were shifted by 5 nm towards the cut-on wavelength of the dichroic mirror resulting in a 10 nm peak-to-peak spectral separation (Supplementary Figure 12b). In this condition, RLSU could still accurately reconstruct the ground truth objects ($\mathrm{RMSE}_\mathrm{S} = 0.823, \mathrm{RMSE}_\mathrm{P} = 0.851$, compared with $\mathrm{RMSE}_\mathrm{S} = 0.998, \mathrm{RMSE}_\mathrm{P} = 1.039$ for linear unmixing). This was also true with a 4 nm peak-to-peak spectral separation (Supplementary Figure 12c, $\mathrm{RMSE}_\mathrm{S} = 1.829, \mathrm{RMSE}_\mathrm{P} = 1.897$ for RLSU and $\mathrm{RMSE}_\mathrm{S} = 2.11, \mathrm{RMSE}_\mathrm{P} = 2.182$ for linear unmixing). However, with a 0 nm spectral separation the marginally different spectral shapes of EGFP and EYFP provided insufficient information for accurate spectral unmixing (Supplementary Figure 12d).

F Selecting the optimum number of channels

A dichroic mirror splits multicolour light into two paths, one transmitted and one reflected. Hence, by arranging dichroic mirrors in a full-branched tree-like format, 2^N colour channels are produced using $2^N - 1$ dichroic mirrors, where N is the number of levels of the tree. One, two and four channels are each an insufficient number (as they would not allow for unmixing more than one, two or four fluorophore species), while 32 channels or more would require an infeasible number of dichroic mirrors. The question is then whether the extra spectral information provided by 16 channels outweighs the significantly greater investment in optics and detectors required compared to an eight-channel system.

As most samples are only labelled with fluorophores emitting in the 450 nm to 650 nm range, we can consider imaging the same 200 nm spectral range with either 16 channels in 12.5 nm wavebands, or eight 25 nm wavebands. A 25 nm bandwidth is similar to that employed in many single-channel imaging systems using a bandpass filter, meaning that raw signal levels would be comparable. Furthermore, Jahr et al. previously showed using their hyperspectral light sheet microscope that a 25 nm bandwidth still provided sufficient information to spectrally unmix EYFP from EGFP [6], two of the most spectrally similar fluorophores in common usage.

This suggests that the only advantage of a 16-channel system would be if more than eight fluorophore species needed to be unmixed (as N channels are required to unmix N species), or if the flexibility to image across a larger spectral range (*i.e.* keeping the same 25 nm waveband width but extending into the far-red and near-infrared) were needed. As such, we selected eight channels, and hence seven dichroic mirrors, as the optimum number. However, we note that, if more than eight, but less than 16, channels are desired, this can be achieved without investment in a full 16-channel system by adopting a non-fully-branched tree layout (*e.g.* nine channels can be produced by just adding one dichroic mirror and an additional

detector to an appropriate path). The number of unmixable components can be extended beyond eight, while maintaining eight detection channels, by also incorporating excitation-based unmixing, but this precludes the possibility of simultaneous capture of all necessary data and hence compromises temporal resolution.

G USAF 1951 target resolution testing

Testing the resolution of the assembled system using a USAF 1951 target showed that at least group 5–6 could be clearly resolved across all channels, corresponding to 57.0 lp/mm (Figure 2e). For camera 1, viewing ~ 465 nm, group 6–3 was resolvable, while 6–4 was not. 6–3 corresponds to 80.6 lp/mm, close to the Nyquist-sampling limit of 80.5 lp/mm.

H Supplementary methods

Minibinder purification and labelling

Biochemical characterisation of the minibinders used in this study is presented in Supplementary Figure 26c. Unless stated otherwise, all purification steps were performed at 4 °C in the cold room. SDS-PAGE was performed using NuPAGE 4–12% Bis-Tris gels (ThermoFisher) according to the manufacturer's instructions. InstantBlue (Sigma) was used for total protein staining of gels. Protein concentrations were determined using a BCA protein assay kit (Pierce) or by absorbance at 280 nm in a Nano Drop spectrophotometer (ThermoFisher) used in cuvette mode. In the latter case, molar absorption coefficients calculated from the amino acid sequences were used, and when characterising minibinders labelled with organic dyes, absorbance at 280 nm was corrected for dye absorbance using the correction factors provided by the manufacturer. In coomassie-stained gel imaging were performed on a ChemiDoc Touch instrument (Bio-Rad).

For integrin $\alpha 5\beta 1$, IGF2R and BMPR2 minibinders, pGEX vectors encoding GST-TEV-3C-KKCKK-Minibinder-10×His (see above) were transformed into E. coli BL21(DE3) (Novagen, 69450-3), and bacteria were grown in 2×TY enriched with 100 µg/ml carbenicillin (Melford, C46000) at 37 °C, with 220 RPM shaking. Once culture reached an OD600 of 0.8, bacteria were then cooled down before induction at at 20 °C overnight (~18 hours) with 0.5 mM isopropylthio-β-galactoside (IPTG, Generon, 02122). Cells were harvested through centrifugation at 4000 *g* for 20 minutes at 4 °C. The bacterial pellet was then stored at −20 °C until protein purification.

For GST-3C-integrin $\alpha 5\beta 1$ minibinder-KKCKK-10×His purification, bacterial pellets were resuspended in 30 ml LYSIS buffer [25 mM HEPES, 150 mM NaCl, 1 mM DTT, 1 mM EDTA, 10 µg/ml DNAse, 1 mM $MgCl_2$, 70 µg/ml lysozyme and cOmplete mini EDTA-free protease inhibitor cocktail (Roche, 11836170001)] per liter of cultured E. coli pellet. Resuspended bacteria were incubated at 4 °C for 15 mins with rolling prior to sonication on ice for 2 minutes 30 seconds (10 seconds on, 30 seconds off, 40 % amplitude). Lysate was then centrifuged at 40 000 *g* for 40 minutes at 4 °C. The soluble supernatant was incubated with 1 ml glutathione sepharose 4B resin (GE Healthcare, 17-0756-01) for 2.5 hours at 4 °C with rolling. After incubation, resin and supernatant was poured into a gravity flow column and resin was washed extensively with LYSIS buffer without protease inhibitors/DNAseI/Lysosyme and eluted with ELUTION buffer [25 mM HEPES, 150 mM NaCl, 1 mM EDTA, 1 mM DTT, 10 mM reduced glutathione, pH 7.2]. To remove the reduced glutathione, eluted protein was dialysed overnight at 4 °C into STORAGE buffer [25 mM HEPES, 150 mM NaCl, 1 mM EDTA, 1 mM DTT, pH 7.2]. After dialysis, protein was incubated with 40 U of GST-tagged HRV 3C Protease (Pierce, 88946) to remove the GST tag and incubated overnight at 4 °C. Free GST and 3C protease were removed by incubating the dialysed protein with 1 ml of fresh glutathione sepharose 4B resin for 1.5 hours at 4 °C with rolling. Upon running through a disposable collumn (Bio-Rad), flow through containing the integrin $\alpha 5\beta 1$ minibinder-KKCKK-10×His was collected and flash frozen in liquid nitrogen for storage.

IGF2R minibinder-KKCKK-10×His was purified in a similar fashion, with few modifications: LYSIS buffer was modified into [25 mM HEPES, 150 mM KCl, 1 mM DTT, 1 mM EDTA, 10 µg/ml DNAse, 1 mM $MgCl_2$, 70 µg/ml lysozyme, 10 % glycerol + cOmplete mini EDTA-free protease inhibitor cocktail, pH 7.4]. GST-bound binder GST-minibinders bound to glutathione sepharose 4B were washed first in WASH1 [25 mM HEPES, 150 mM KCl, 1 mM DTT, 1 mM EDTA, 10 % glycerol, pH 7.4], secondly in WASH2 [WASH1 with 500 mM KCL final] and finally washed in WASH1 again. ELUTION buffer was [25 mM HEPES, 150 mM KCl, 1 mM DTT, 1 mM EDTA with 20 mM reduced glutathione, pH 8]. Eluted protein was dialysed overnight in [25 mM HEPES, 150 mM KCl, 1 mM DTT, 1 mM EDTA, pH 7.4] followed by further dialysis into STORAGE buffer [25 mM HEPES, 150 mM KCl, 1 mM EDTA, pH 7.4], before GST and 3C removal as above.

Similarly, for BMPR2 minibinder-KKCKK-10×His, LYSIS buffer was [25 mM HEPES pH 8, 150 mM NaCl, 1 mM DTT, 1 mM EDTA, 10 µg/ml DNAse, 1 mM $MgCl_2$, 70 µg/ml lysozyme + cOmplete mini EDTA-free protease inhibitor cocktail]. GST-minibinders bound to glutathione sepharose 4B were washed first in WASH1 [25 mM HEPES pH 8, 150 mM NaCl, 1 mM EDTA, 1 mM DTT] and then washed in WASH2 [25 mM HEPES pH 8, 500 mM NaCl, 5 mM betamercaptoethanol, 20 % glycerol, 0.003 % DDM] and then washed a final time with WASH1. ELUTION buffer was [25 mM HEPES pH 8, 150 mM NaCl, 1 mM DTT, 1 mM EDTA and 10 mM reduced glutathione]. Eluted protein was incubated with 40 U of HRV 3C Protease to remove the GST tag and incubated overnight at 4 °C with simultaneous dialysis into [25 mM HEPES, 150 mM NaCl, 1 mM DTT, 1 mM EDTA, pH 7.4] followed by further dialysis the next day into STORAGE buffer [25 mM HEPES, 150 mM NaCl, 1 mM EDTA, pH 7.4]. GST and 3C protease were removed as above.

Single cysteine versions of a transferrin receptor minibinder (2DS25), as well as an interface knockout (KO) variant abolishing receptor binding were expressed and purified as previously reported [24].

Minibinder fluorescent labelling

Biochemical characterisation, in particular in gel fluorescence of the minibinders used in this study is presented in Supplementary Figure 26c, and validation of their use in cells is presented in Supplementary Figure 27. For labelling of transferrin receptor minibinder, and respective interface knockout control, a 10-fold molar excess of Alexa Fluor 488 C5 maleimide (ThermoFisher) or maleimide Alexa Fluor 647 C2 maleimide (ThermoFisher) was added dropwise whilst vortexing to a 200 µL solution of minibinder at 1 mg/ml in [20 mM Tris pH 7.4, 100 mM NaCl, 1 mM TCEP]. After incubation overnight in the dark at 4 °C with rocking, free dye was removed through 6 sequential rounds of dilution into 500 µL PBS followed by concentration down to 50 µL using an Amicon Ultra 0.5 ml centrifugal filter unit with a 3 kDa MWCO (Pierce).

For IGF2R minibinder labelling, a 10-fold molar excess of ATTO 514 maleimide (ATTO-TEC) was added dropwise to a 200 µL solution of minibinder in [SI25mM HEPES, SI150mM mM NaCl, SI1mM EDTA, pH 8] whilst vortexing, followed by a 3-hour incubation at room temperature with rocking in the dark. Excess free dye was removed through 9 sequential rounds of dilution into 500 µL PBS followed by concentration down to 50 µL using an Amicon Ultra 0.5 ml centrifugal filter unit with a 10 kDa MWCO.

For BMPR2 minibinder labelling, 10 µL of Alexa Fluor 647 C2 maleimide (ThermoFisher) was added dropwise to 1 mL of purified binder (10 µM) in STORAGE buffer [25 mM HEPES, 150 mM NaCl, 1 mM EDTA, pH 7.4], followed by a 2-hour incubation at room temperature with rocking in the dark. This ratio provides a 10-fold molar excess of dye over protein. Despite multiple attempts with different techniques, the excess free Alexa Fluor 647 dye could not be removed, but control experiments using the same amount of quenched dye alone showed that free dye at the low concentrations we use for binder experiments does not lead to appreciable endosomal signal (data not shown).

For integrin $\alpha5\beta1$ minibinder labelling, purified binder was dialised against PBS to remove the DTT present in the STORAGE buffer. Then 25 µL of 0.2 M sodium bicarbonate, pH 9 was added to 250 µL protein in PBS to increase pH to 8.3 followed by dropwise addition of 10 µL of a 10 mM solution of CF680R NHS in DMSO while vortexing. After a 2-hour incubation at room temperature with rocking in the dark, free dye was removed through seven sequential rounds of dilution into 500 µL PBS followed by concentration down to 50 µL using an Amicon Ultra 0.5 ml centrifugal filter unit with a 3 kDa MWCO.

Fluorescent minibinder uptake

To monitor receptor trafficking at endogenous levels (Figure 6), we used a pulse-chase experimental design with the purified fluorescent minibinders described above. HeLa Kyoto cells transiently expressing NLS-TagBFP for 24h were plated onto fibronectin-coated imaging dishes with a small 10 mm glass coverslip (World Precision Instruments, FD35-10) and allowed to adhere for one hour in 50 µl of DMEM media enriched with 10 % FBS and 1 % P/S. After the cells had adhered, the medium was removed and replaced with 50 µl of ice-cold DMEM containing 5 µl of TfR binder Alexa Fluor 488 (stock concentration 90 µM, so 6 µM final), 2 µl of IGF2R binder ATTO 514 (stock concentration 21 µM, so 0.6 µM final), 10 µl of BMPR2 binder Alexa Fluor 647 (stock concentration 10 µM, so 1.3 µM final), 5 µl of I$\alpha5\beta1$ binder CF680R (stock concentration 200 µM, so 14 µM final) and 2 µg/ml of EGF Alexa Fluor 555 (catalogue number: E35350, ThermoFisher), all in PBS. Cells were then kept on ice for 30 minutes to prevent endocytosis and allow for fluorescently-labelled binder proteins to bind to their respective receptors. Note that the medium did not contain FBS in order to serum-starve the cells, which causes receptors to cluster at the plasma membrane. After 30 minutes on ice, the medium was removed, the cells were washed five times with PBS to remove excess unbound minibinder and the medium was replaced with 50 µl of pre-warmed L15 medium supplemented with 10 % FBS, 4.5 g/l of glucose and 20 mM of HEPES to trigger receptor endocytosis. After 10 minutes in the pre-warmed media the cells were imaged on the multispectral OPM. The 405, 488, 561 and 638 nm lasers were used to excite the six fluorophores in the sample simultaneously and the resulting emission was recorded for every plane on the eight cameras of the multispectral acquisition module. Eight-channel full cell volumes were imaged between 1-3 Hz, as per the imaging conditions described in Supplementary Table 3.

To show that each minibinder could be uptaken by cells individually (Supplementary Figure 27a), HeLa Kyoto cells were incubated with indicated minibinders at the same concentration as we used in the multiplexed uptake experiment described above, replacing the other binders by a corresponding volume of PBS, and cells were imaged on the multispectral OPM as above. To demonstrate that endocytosed minibinders indeed reached early-sorting endosomes, NIH-3T3 stably expressing GFP-WASH, a sorting endosome marker, were plated onto fibronectin-coated imaging dishes as above and incubated with 50 µl of L15 media supplemented with 10 % DCS, 20 mM of HEPES, 4.5 g/l glucose and with either 5 µl of TfR binder Alexa Fluor 647 (stock concentration 8.5 µM, so 0.7 µM final) or 10 µl of BMPR2 binder Alexa Fluor 647 (stock concentration 10 µM, so 1.7 µM final). The cells were incubated with the binders on the microscope at 37 °C for 10 minutes to allow receptor internalisation prior to imaging using the single channel spinning disk confocal microscope described above.

Receptor	Abbreviation	Size / aa	Affinity / nM	Fluorophore
Transferrin receptor	TfRB	88 (128)	30	Alexa Fluor 488
Insulin-like growth factor 2 receptor	IGF2RB	124 (164)	40	ATTO 514
Bone morphogenetic protein receptor type 2	BMPR2B	59 (99)	4	Alexa Fluor 647
Integrin $\alpha 5\beta 1$	I$\alpha 5\beta 1$B	65 (105)	2.2	CF680R

Supplementary Table 2: De novo designed protein-binding protein details.
For size column, number in brackets includes PC-tag, N- and C-terminal linkers and 10×His tag.

Figure	Modality	Cell type	Pixel pitch / nm	Z spacing / nm	Exposure / ms	Interval / s	N_Z	N_C	N_T	λ_{ex} /nm	Labels	LUTs
3a,c,d	SDCM	U2OS (37 °C)	98.5	N/A	100	15	1	7	50	405	NLS-TagBFP	Blue
											Lyn-Cerulean	Duo intense cyan
										470	MTS-mAzamiGreen	Red
											GTS-Citrine	Green
										555	LysoTracker Yellow HCK-123	Magenta
											mCherry-KDEL	Circus yellow
										640	iRFP670-SKL	Cyan
3e	SDCM	U2OS (37 °C)	98.5	N/A	100	15	1	6	100	405	NLS-TagBFP	Blue
											Lyn-Cerulean	Duo intense cyan
										470	MTS-mAzamiGreen	Red
											GTS-Citrine	Green
										555	mCherry-KDEL	Circus yellow
										640	iRFP670-SKL	Magenta
3f	SDCM	U2OS (37 °C)	98.5	N/A	200	15	1	7	25	405	NLS-TagBFP	Blue
											Lyn-Cerulean	Duo intense cyan
										470	MTS-mAzamiGreen	Red
											GTS-Citrine	Green
										555	SPY555-tubulin	Pop cyan
											mCherry-KDEL	Circus yellow
										640	iRFP670-SKL	Magenta
3g	SDCM	U2OS (37 °C)	98.5	N/A	300	45	1	7	25	405	NLS-TagBFP	Blue
											Lyn-Cerulean	Grey
										470	MTS-mAzamiGreen	Red
											GTS-Citrine	Green
										555	SPY555-actin	Magenta
											mCherry-KDEL	Circus yellow
										640	iRFP670-SKL	Cyan
4a	OPM	U2OS (37 °C)	111.6	500	250	10	201	6	1	405	NLS-TagBFP	Blue
											Lyn-Cerulean	Duo intense cyan
										488	MTS-mAzamiGreen	Red
											GTS-Citrine	Green
										561	mCherry-KDEL	Circus yellow
										638	iRFP670-SKL	Grey

Supplementary Table 3: Experimental details.
N_Z is the number of z-planes acquired, N_C is the number of different fluorophores used, N_T is the number of timepoints and λ is the wavelength of laser excitation used. LUTs refers to the ImageJ lookup-tables used to visualise data.

Figure	Modality	Cell type	Pixel pitch / nm	Z spacing / nm	Exposure / ms	Interval / s	N_Z	N_C	N_T	λ_{ex} /nm	Labels	LUTs
5	OPM	U2OS (37 °C)	111.6	Projection	100	0.1	N/A	4	500	488	MTS-mAzamiGreen	Green
											GTS-Citrine	Magenta
										561	mCherry-KDEL	Circus yellow
										638	iRFP670-SKL	Cyan
6	OPM	HeLa (37 °C)	111.6	750	19	1.3	67	6	35	405	NLS-TagBFP	Blue
										488	Alexa Fluor 488 TfR binder	Quartetto red
											ATTO 514 IGFR2 binder	Quartetto yellow
										561	Alexa Fluor 555 EGF	Quartetto green
										638	Alexa Fluor 647 BMPR2 binder	Quartetto magenta
											CF680R Iα5β1 binder	Cyan
SF23	SDCM	U2OS (37 °C)	98.5	200	100	45	51	5	16	405	Lyn-Cerulean	Duo intense cyan
										470	MTS-mAzamiGreen	Red
											GTS-Citrine	Green
										555	mCherry-KDEL	Circus yellow
										640	iRFP670-SKL	Magenta
SF25	OPM	*D. discoideum* (20 °C)	111.6	750	20	0.5	27	2	40	488	PIPpkgE-PH-EGFP	Duo intense cyan
										561	LifeAct-mCherry	Duo intense yellow
SF27a	OPM	NIH 3T3 (37 °C)	111.6	750	50	1	81	1	1	488	Alexa Fluor 488 TfR binder	Black ink wash
											ATTO 514 IGF2R binder	
										638	Alexa Fluor 647 BMPR2 binder	
											CF680R Iα5β1 binder	
SF27b	SDCM	NIH 3T3 (37 °C)	110	N/A	120	1	N/A	2	121	488	EGFP-WASH	Green
										638	Alexa Fluor 647 TfR binder	Magenta
SF27c	SDCM	NIH 3T3 (37 °C)	110	N/A	120	1	N/A	2	50	488	EGFP-WASH	Green
										638	Alexa Fluor 647 BMPR2 binder	Magenta
SF27d,e	SDCM	NIH 3T3 (37 °C)	110	N/A	120	1	N/A	1	1	488	EGFP-WASH	Black ink wash
											Alexa Fluor 488 TfR binder	

[Supplementary Table 3 continued]

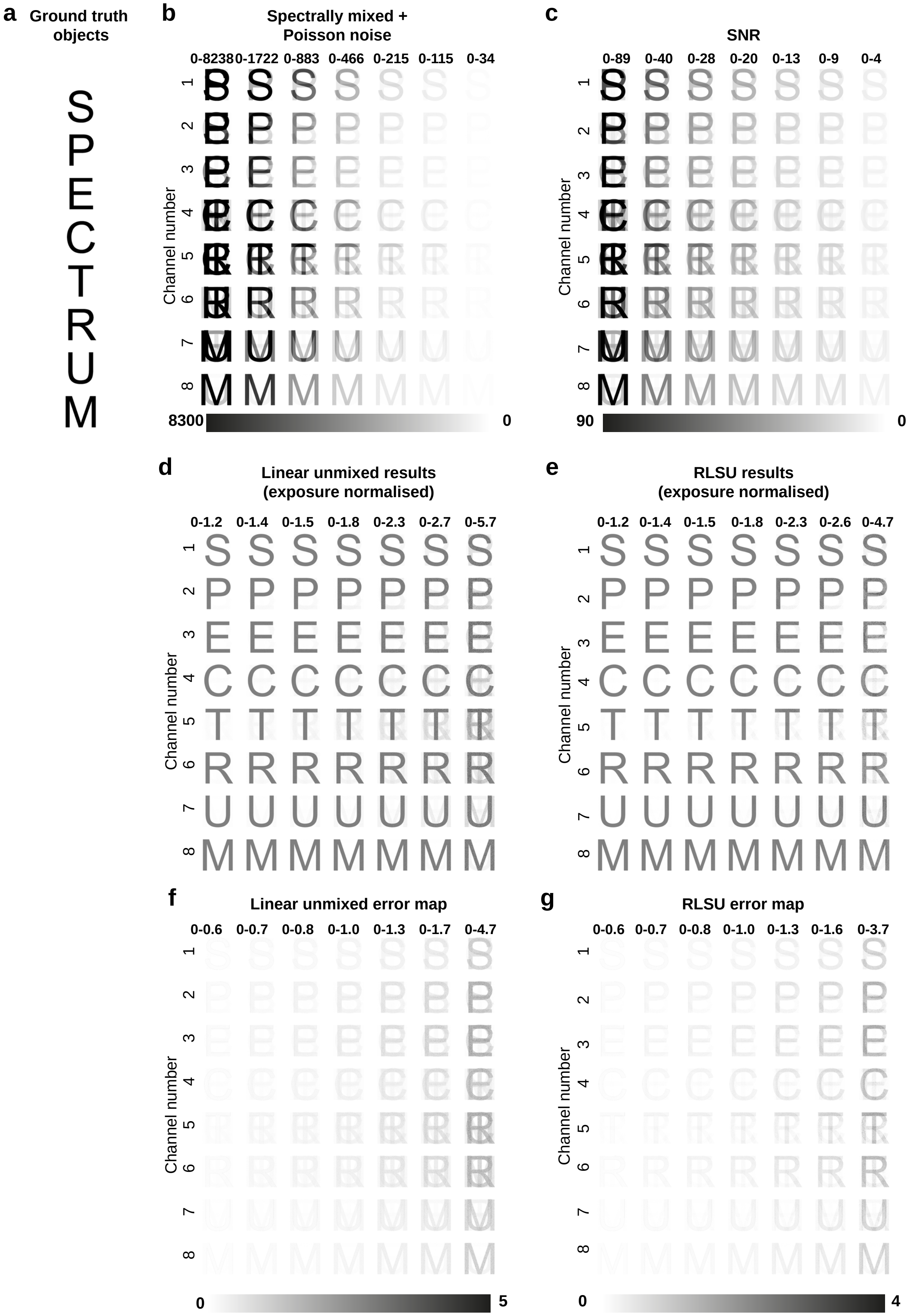

Supplementary Figure 1: RLSU outperforms linear unmixing for unmixing simulated multispectral datasets with varying SNR.
a Ground truth objects. **b** Mixed data incorporating Poisson (shot) noise, generated from (a) at different signal levels (high signal – low signal, right – left, respectively). This simulates the detection of mixed images with reducing exposure time. **c** SNR maps of data shown in (b). Linearly unmixed (**d**) and RLSU unmixed (**e**) results of data shown in (b). Results are normalised to the exposure time used to generate (b). **f-g** Error maps of data shown in (d-e). RLSU produces fewer errors than linear unmixing when reconstructing objects from mixed noisy images at every SNR tested. Data shown in grayscale inverted LUT with the range of pixel values shown numerically above each column.

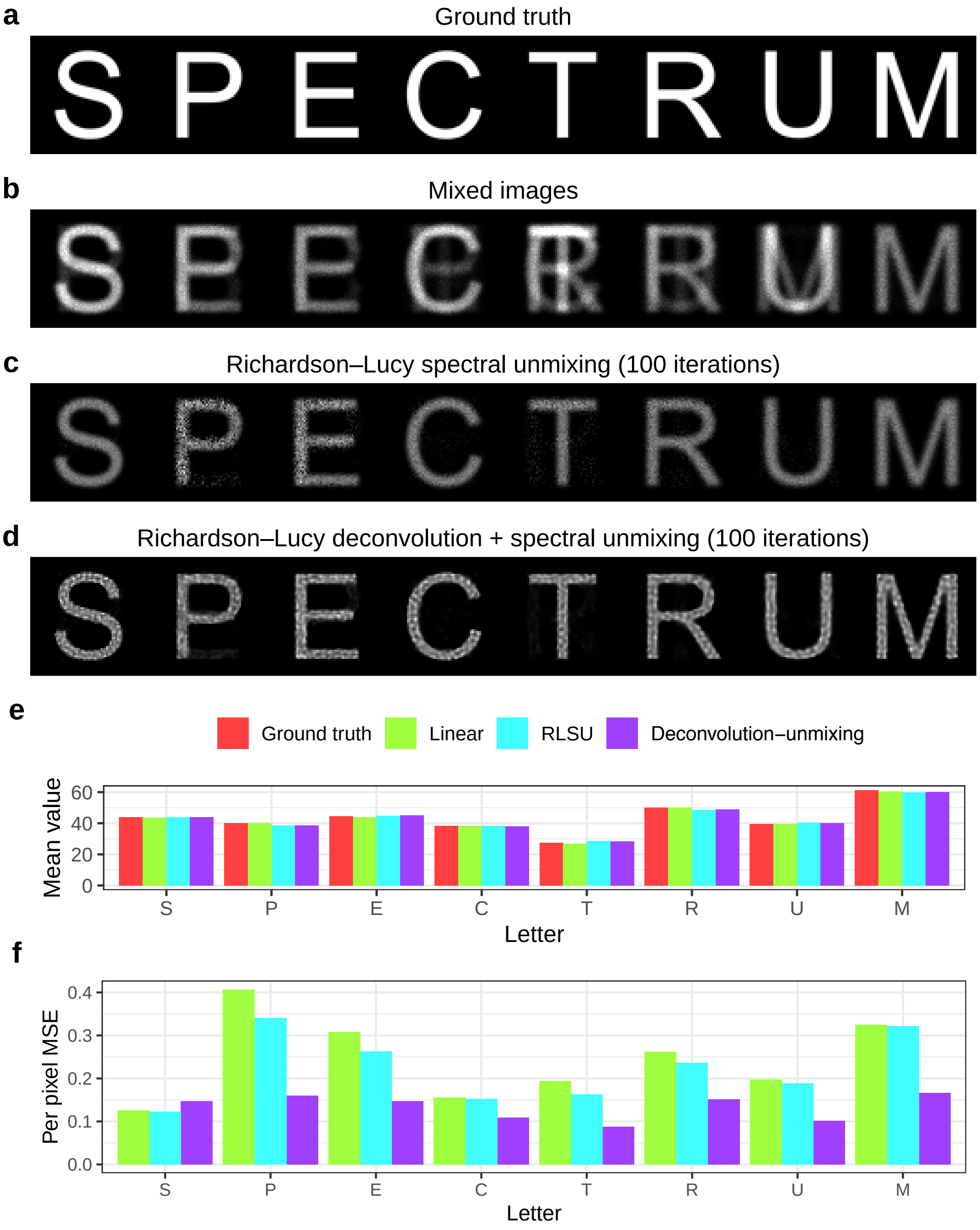

Supplementary Figure 2: Richardson-Lucy deconvolution and spectral unmixing.
a Ground truth objects, taken as letters from the word SPECTRUM. **b** Blurred, mixed noisy raw data, generated from (a). **c** Unmixed objects produced using RLSU without deconvolution after 100 iterations. **d** Unmixed objects produced using simultaneous Richardson–Lucy deconvolution-unmixing after 100 iterations. **e** Mean pixel values for each object (ground truth) or unmixed reconstructions resulting from linear unmixing, RLSU or simultaneous RL deconvolution-unmixing. **f** Per pixel mean squared error for unmixed reconstructions resulting from linear unmixing, RLSU or simultaneous RL deconvolution-unmixing.

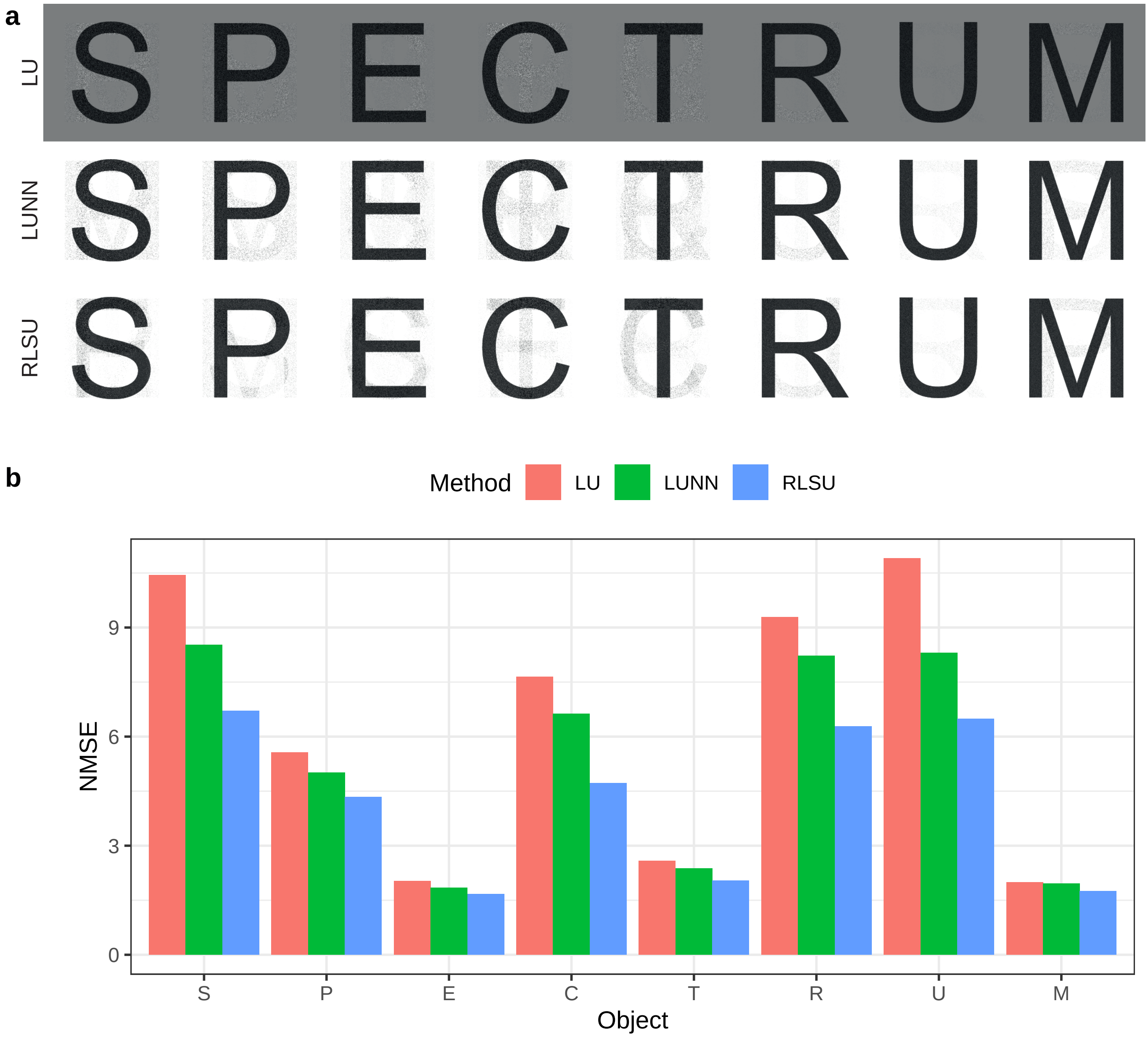

Supplementary Figure 3: RLSU outperforms linear unmixing with negative values set to zero.
a Results for unmixing the data shown in Figure 1 using linear unmixing (LU), linear unmixing with negative values set to zero (LUNN) and RLSU. Images are shown in an inverted lookup table for clarity. Note that, particularly for the letters C and T, RLSU results have less bleedthrough from other letters compared to the other two methods. **b** Normalised mean square errors (NMSE) for the results shown in a. For all letters RLSU produces the lowest squared error.

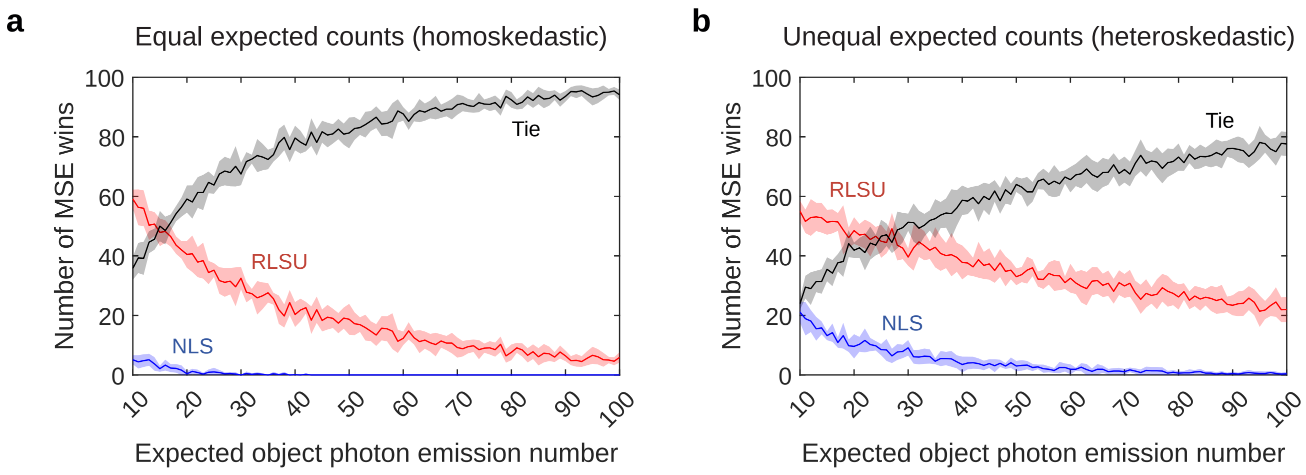

Supplementary Figure 4: Comparison of RLSU with NLS spectral unmixing.
Each plot shows the number of wins in the simulations detailed in Supplementary Note C, i.e. a result with smaller mean square error (MSE) than the competing algorithm, with RLSU in red, NLS in blue and the number of ties in black. The shaded regions correspond to one standard deviation over 10 trials of 100 noisy datasets. **a** Results for the equal expected counts (and therefore homoskedastic) mixing matrix. **b** Results for the unequal expected counts (and therefore heteroskedastic) mixing matrix. In both cases RLSU performs better than NLS at low photon counts and still outperforms NLS at higher photon counts in the heteroskedastic case.

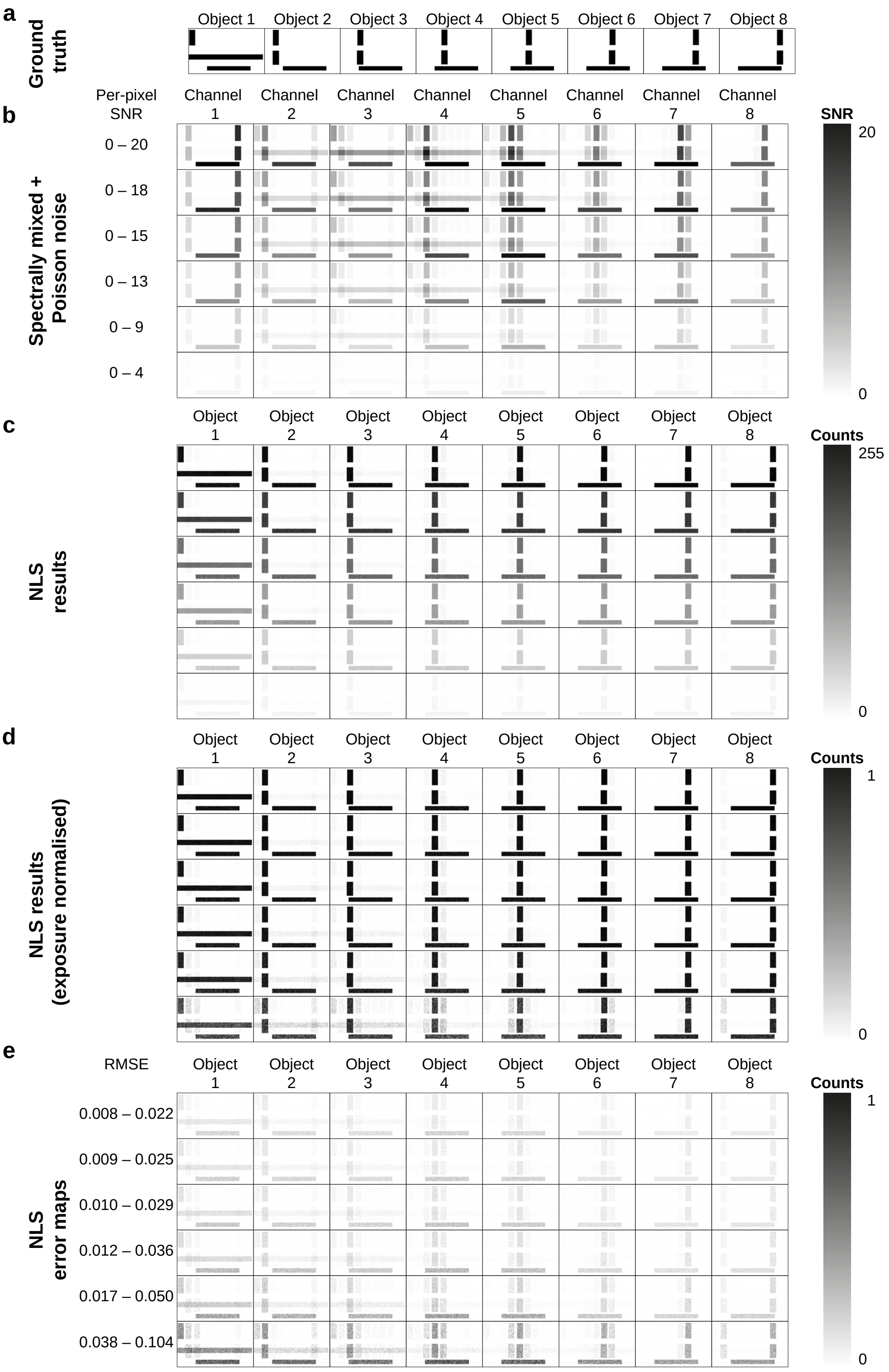

Supplementary Figure 5: Assessment of NLS performance for unmixing simulated multispectral data of varying SNR.
a Simulated ground truth objects. **b** Mixed data incorporating Poisson (shot) noise, generated from (a) at different signal levels (high signal – low signal, top – bottom, respectively). This simulates the detection of mixed images with reducing exposure time. **c** Unmixed reconstructions using NLS. **d** Exposure-normalised reconstructions using NLS. Results are normalised to the exposure time used to generate (b). **e** Error maps of NLS reconstructions. RMSE range shown for all eight unmixed objects.

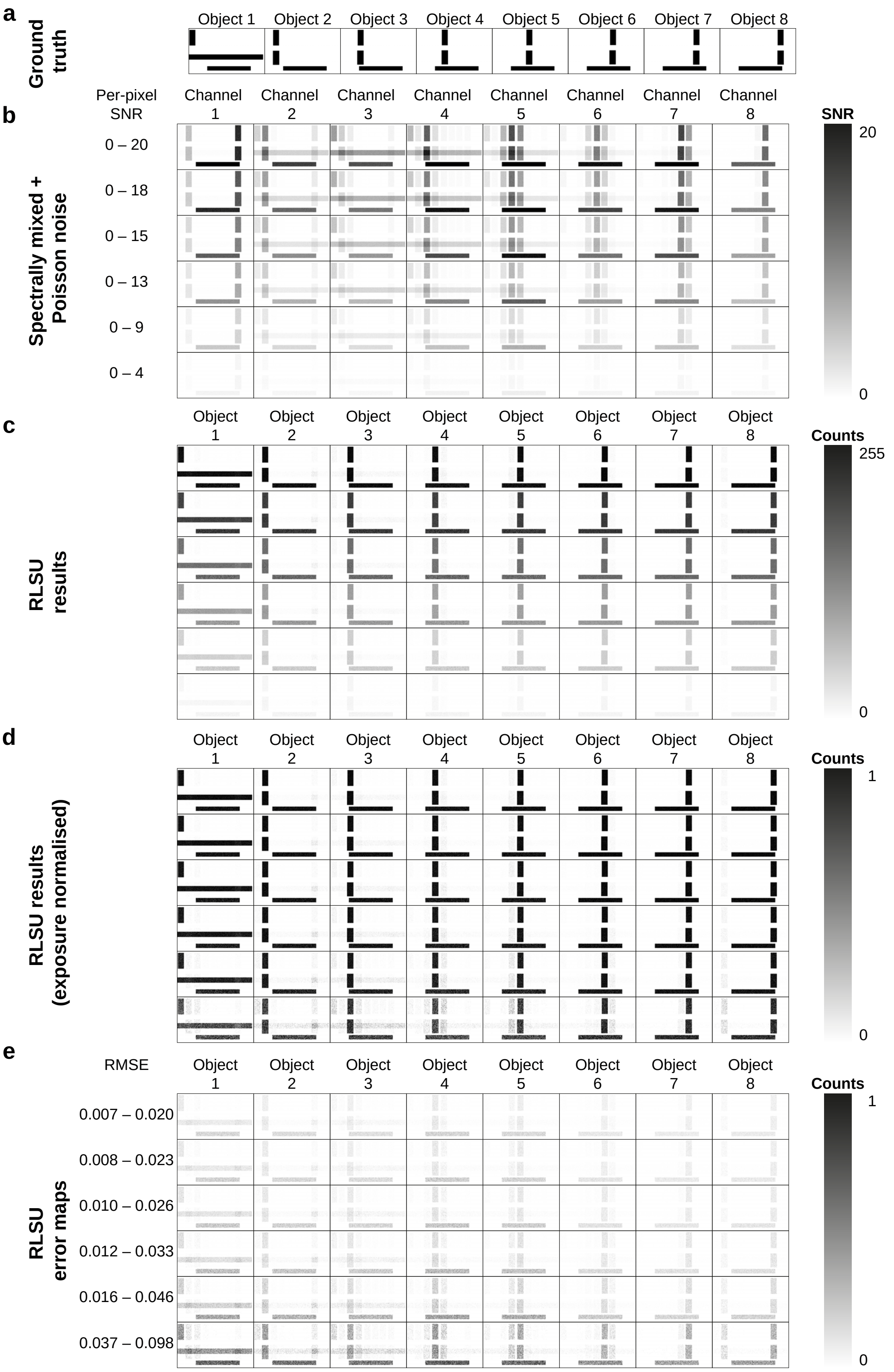

Supplementary Figure 6: Assessment of RLSU performance for unmixing simulated multispectral data of varying SNR.
a Simulated ground truth objects. **b** Mixed data incorporating Poisson (shot) noise, generated from (a) at different signal levels (high signal – low signal, top – bottom, respectively). This simulates the detection of mixed images with reducing exposure time. **c** Unmixed reconstructions using RLSU. **d** Exposure-normalised reconstructions using RLSU. Results are normalised to the exposure time used to generate (b). **e** Error maps of RLSU reconstructions. RMSE range shown for all eight unmixed objects.

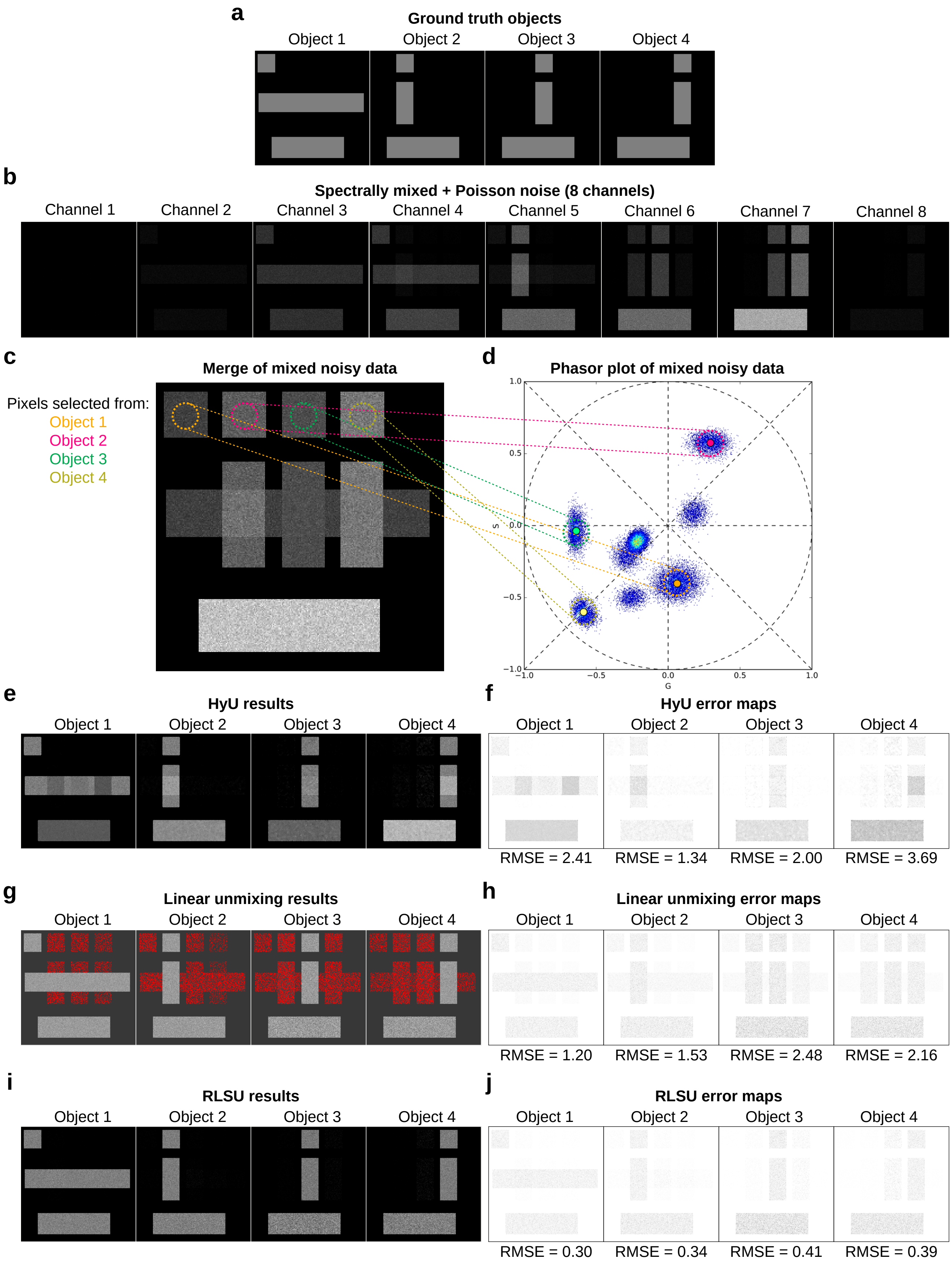

Supplementary Figure 7: RLSU outperforms phasor-based unmixing (HyU) at unmixing simulated eight-channel multispectral data. **a** Four ground truth objects. **b** Mixed, Poisson-noisy 8-channel data, generated from (a), designed to be similar to data acquired from our multispectral imaging module. **c** Overlay of data in (b). Coloured dashed circles represent ROIs in the mixed data with pixels deriving solely from object 1 (orange), 2 (magenta), 3 (green) or 4 (yellow). **d** Phasor plot of data in (b). Coloured dashed circles represent pixels associated with single-labelled objects in phasor space. **e** Unmixed object reconstructions generated using HyU. **f** Pixel-wise error map (inverted LUT) of HyU reconstructions, with root mean square error (RMSE) noted below. **g** Unmixed reconstructions using linear unmixing. Red pixels indicate negative pixel values. **h** Error map (inverted LUT) of linear unmixed reconstructions, with RMSEs below. **i** Unmixed object reconstructions generated using RLSU. **j** Error map (inverted LUT) of RLSU reconstructions, with RMSEs below.

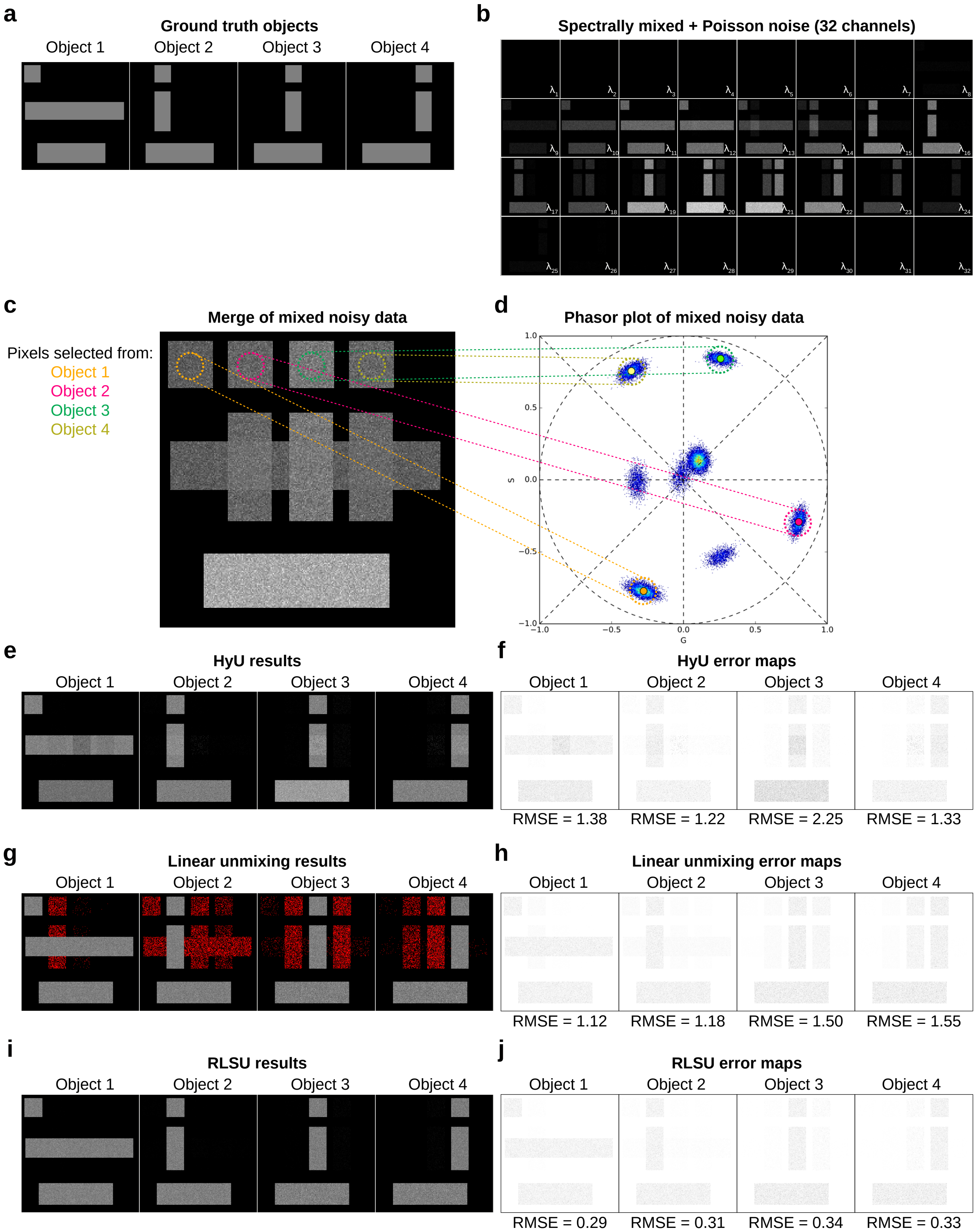

Supplementary Figure 8: RLSU outperforms phasor-based unmixing (HyU) at unmixing simulated 32-channel multispectral data. **a** Four ground truth objects. **b** Mixed, Poisson-noisy 32-channel data, generated from (a), designed to be similar to data acquired from Zeiss QUASAR detectors. **c** Overlay of data in (b). Coloured dashed circles represent ROIs in the mixed data with pixels deriving solely from object 1 (orange), 2 (magenta), 3 (green) or 4 (yellow). **d** Phasor plot of data in (b). Coloured dashed circles represent pixels associated with single-labelled objects in phasor space. **e** Unmixed object reconstructions generated using HyU. **f** Pixel-wise error map (inverted LUT) of HyU reconstructions, with root mean square error (RMSE) noted below. **g** Unmixed reconstructions using linear unmixing. Red pixels indicate negative pixel values. **h** Error map (inverted LUT) of linear unmixed reconstructions, with RMSEs below. **i** Unmixed object reconstructions generated using RLSU. **j** Error map (inverted LUT) of RLSU reconstructions, with RMSEs below. Error maps are shown in inverted LUT with darker pixels representing larger errors.

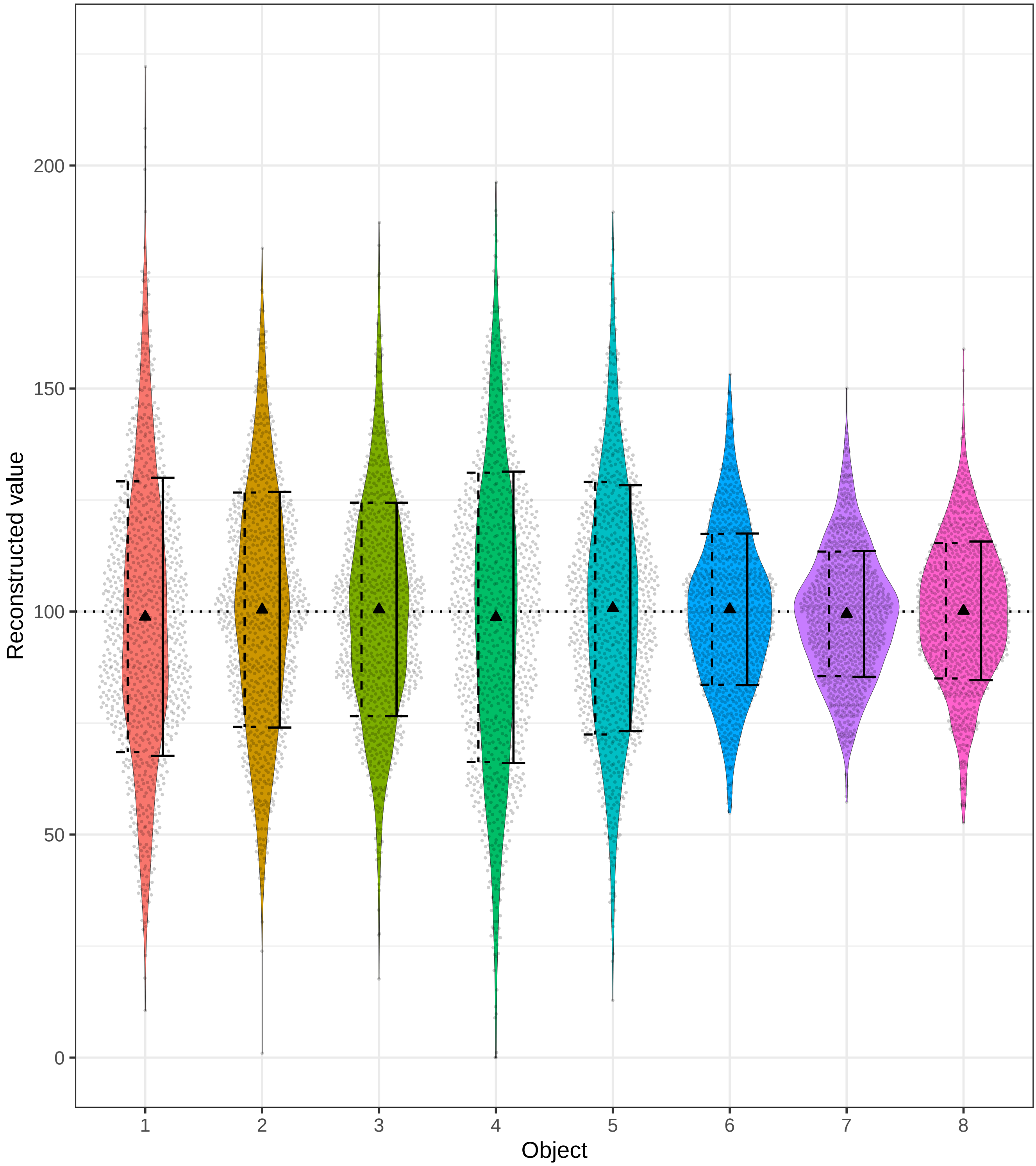

Supplementary Figure 9: Cramér–Rao lower bound analysis.
Distribution of RLSU reconstructed values from 1000 independent sets of Poisson-noisy mixed signals for eight ground truth objects of value 100 (see Supplementary Note D for details). Black triangles show the mean of the 1000 values, correctly sitting around 100. Dashed error bars show the square root of the appropriate Cramér–Rao lower bound, with the standard deviation of the 1000 independent reconstructions shown as a solid error bar.

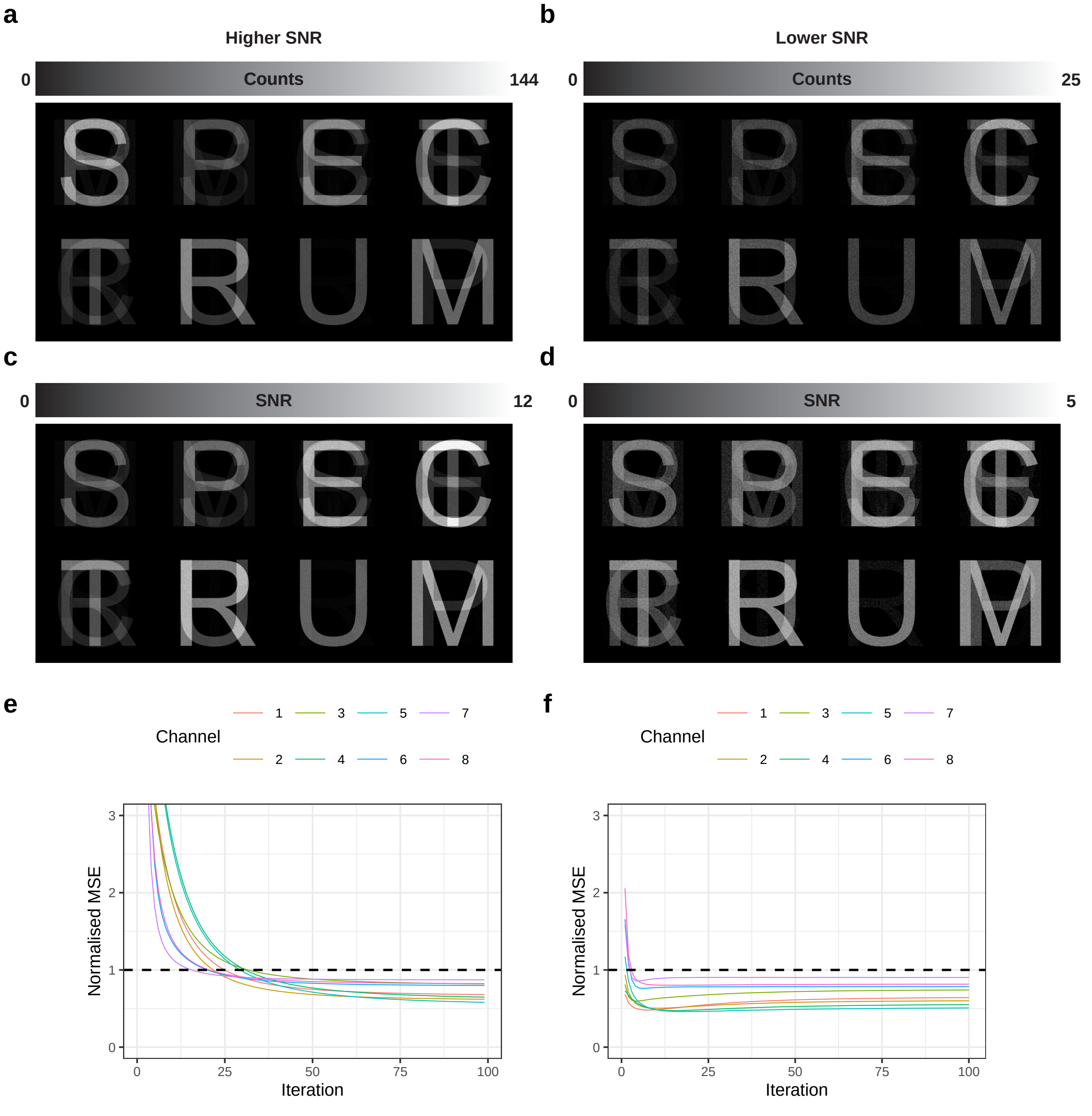

Supplementary Figure 10: RLSU outperforms linear unmixing for unmixing both low and high SNR simulated multispectral datasets. **a** Simulated mixed noisy images of higher SNR data. **b** Simulated mixed noisy images of lower SNR data. **c** SNR map of (a). **d** SNR map of (b). **e** Normalised mean squared error of unmixed results at different iterations of RLSU versus linear unmixing for unmixing higher SNR data (a). Normalised mean squared error (RLSU) shown for all eight channels in coloured lines, whilst the linear unmixing result is indicated by dashed horizontal black line. **f** Normalised mean squared error of unmixed results at different iterations of RLSU versus linear unmixing for unmixing lower SNR data (b). Normalised mean squared error (RLSU) shown for all eight channels in coloured lines, whilst the linear unmixing result is indicated by dashed horizontal black line. Note that in both cases within 100 iterations RLSU converges to a solution and outperforms linear unmixing in all eight channels.

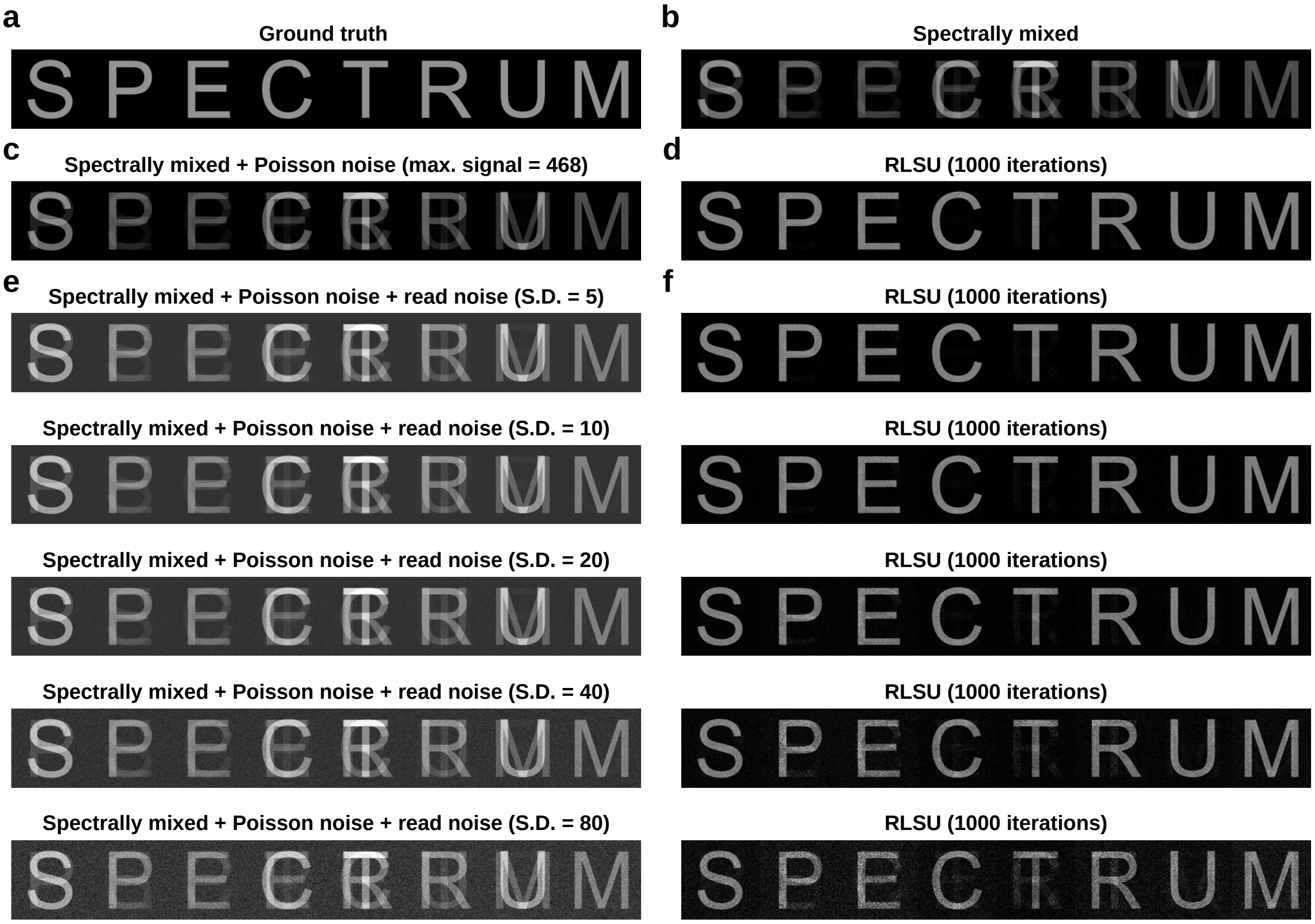

Supplementary Figure 11: RLSU unmixing of simulated data incorporating Poisson (shot) and read noise.
a Eight simulated ground truth objects corresponding to different fluorophores. **b** Spectrally mixed, noise-free data. **c** Spectrally mixed data with Poisson (shot) noise incorporated, and a maximum realised signal of 468 counts. **d** RLSU reconstruction of ground truth objects from data in (c). **e** Data from (c) with simulated camera background (100 counts) and increasing amounts of Gaussian-distributed read noise. **f** RLSU reconstructions from data in (e). 100 counts were removed prior to unmixing to remove simulated 'camera background'. Results are only noticeably different from the zero-read-noise case at the standard deviation = 20 level (i.e. when the variance of the read noise is comparable to the variance of the Poisson (shot) noise), a much higher level of read noise than found in modern sensors.

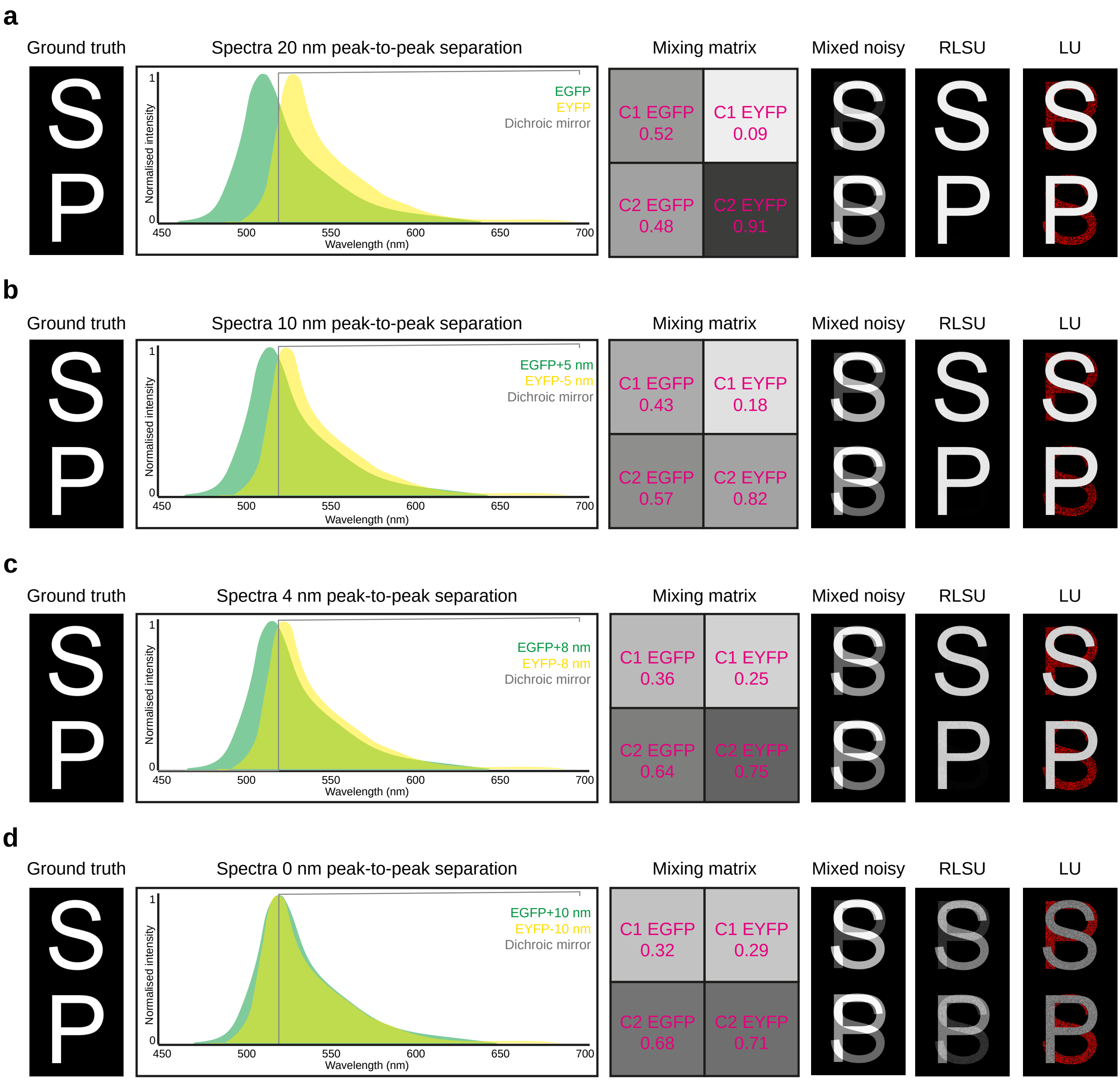

Supplementary Figure 12: Comparison between RLSU and LU for unmixing simulated 2-channel data of increasing spectral overlap. **a** Ground truth, spectra, mixing matrix, mixed images, RLSU result and LU result for 20 nm peak-to-peak spectral separation. **b** Ground truth, spectra, mixing matrix, mixed images, RLSU result and LU result for 10 nm peak-to-peak spectral separation. **c** Ground truth, spectra, mixing matrix, mixed images, RLSU result and LU result for 4 nm peak-to-peak spectral separation. **d** Ground truth, spectra, mixing matrix, mixed images, RLSU result and LU result for 0 nm peak-to-peak spectral separation. See Supplementary Note E.

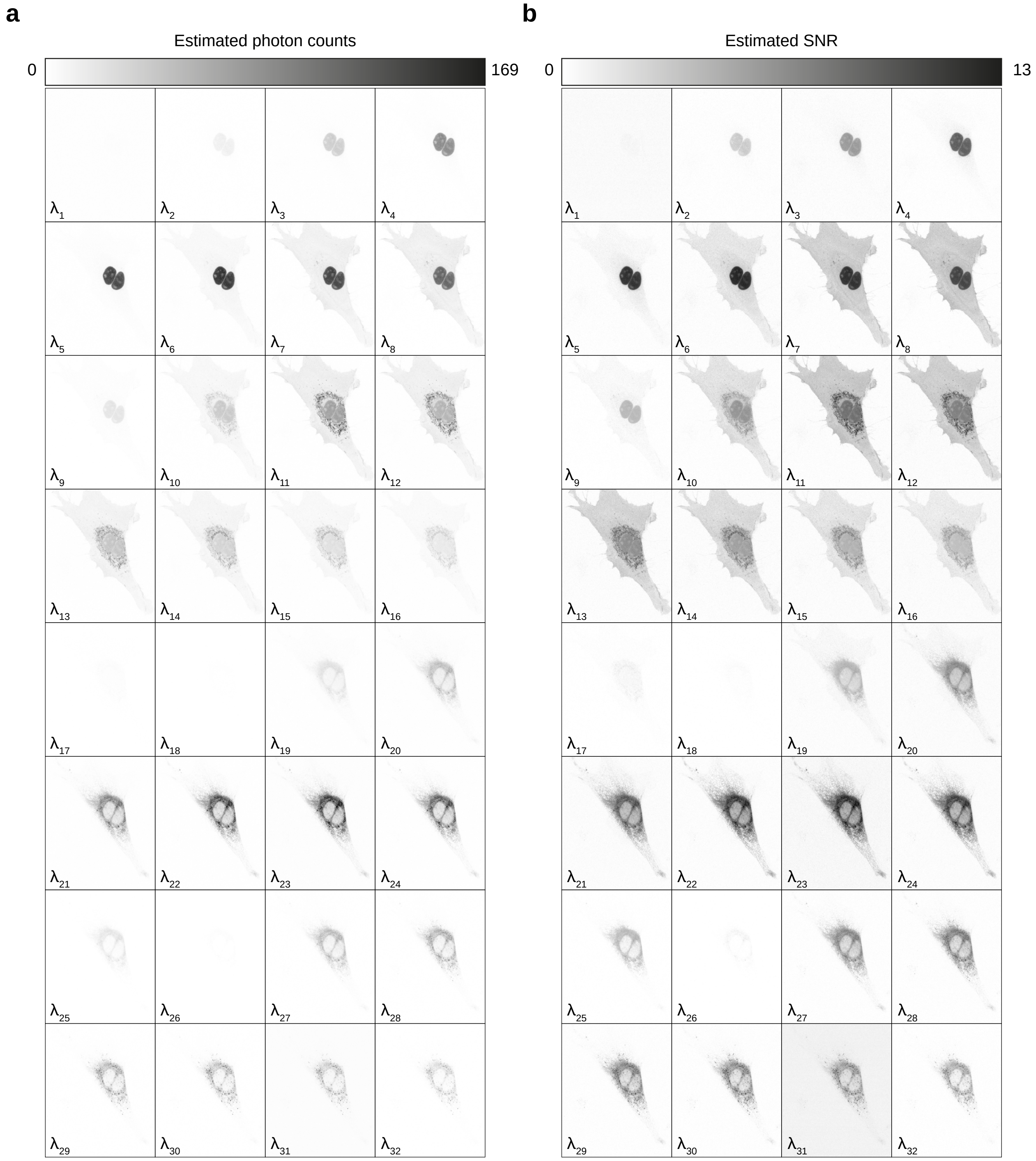

Supplementary Figure 13: Estimated SNR of Zeiss QUASAR data.
a Raw 32 channel data of a live U2OS cell transfected with ColorfulCell. Estimated photon counts are shown for each channel. **b** Estimated SNR values for data in (a) shown for each channel. An inverted LUT is used with darker pixels representing higher SNR. SNRs were calculated using the single-image approach of Heintzmann et al., as detailed in the Methods section.

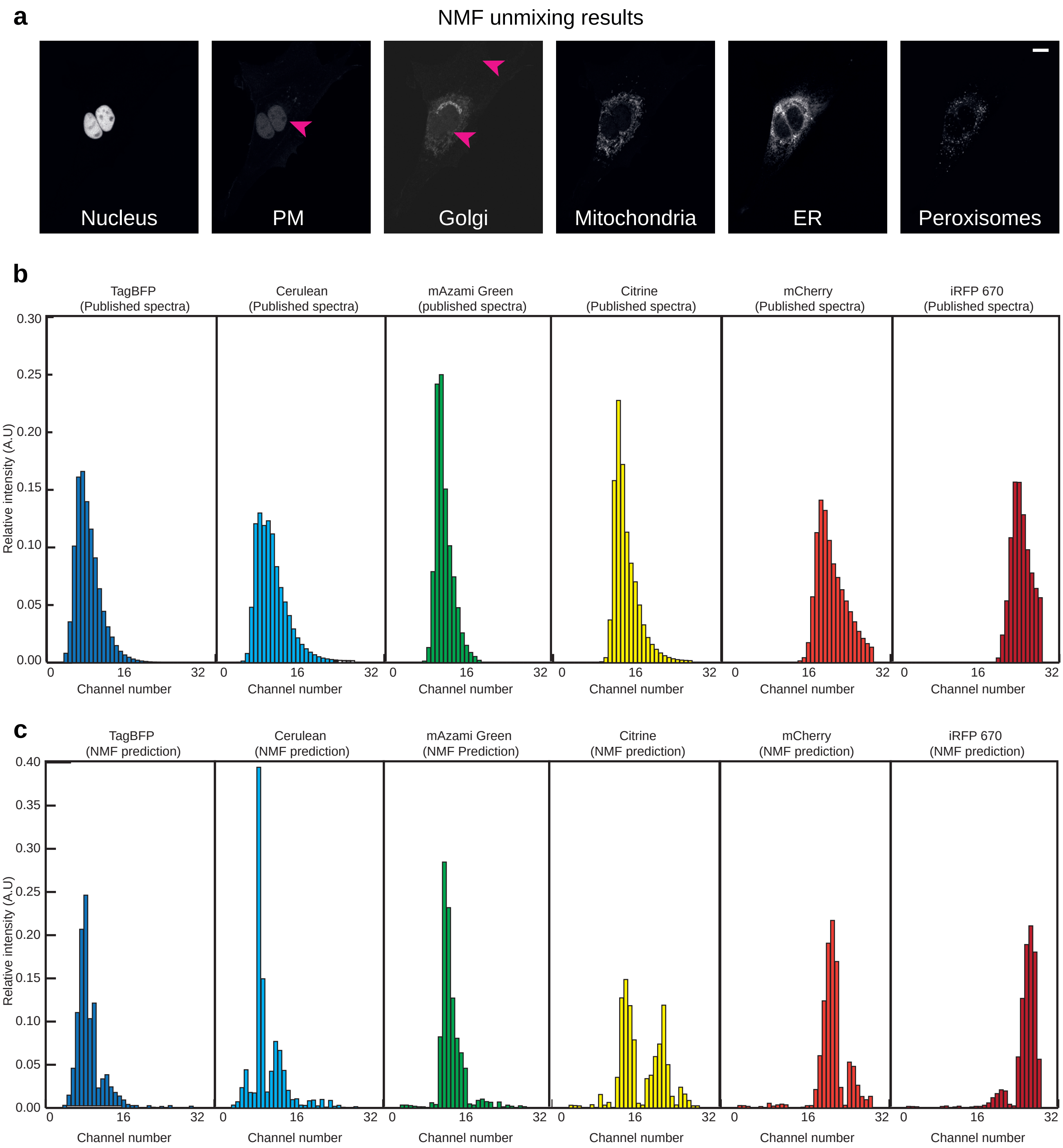

Supplementary Figure 14: NMF produces erroneous reconstructions of ground truth objects and inaccurate predictions of the mixing matrix when unmixing multispectral live-cell imaging data from a Zeiss QUASAR spectral detector.
a NMF reconstructions from a multispectral live-cell imaging experiment. Magenta arrows indicate examples of erroneous pixel reassignment. **b** Graphical depiction of the mixing matrix, formed from published emission spectra, for each of the six fluorophores used to label the sample. Each subplot represents the proportion of the emitted signal collected in each of the 32-channels. **c** Same as in (b) but for the mixing matrix estimated by NMF. Scale bar = 10 μm.

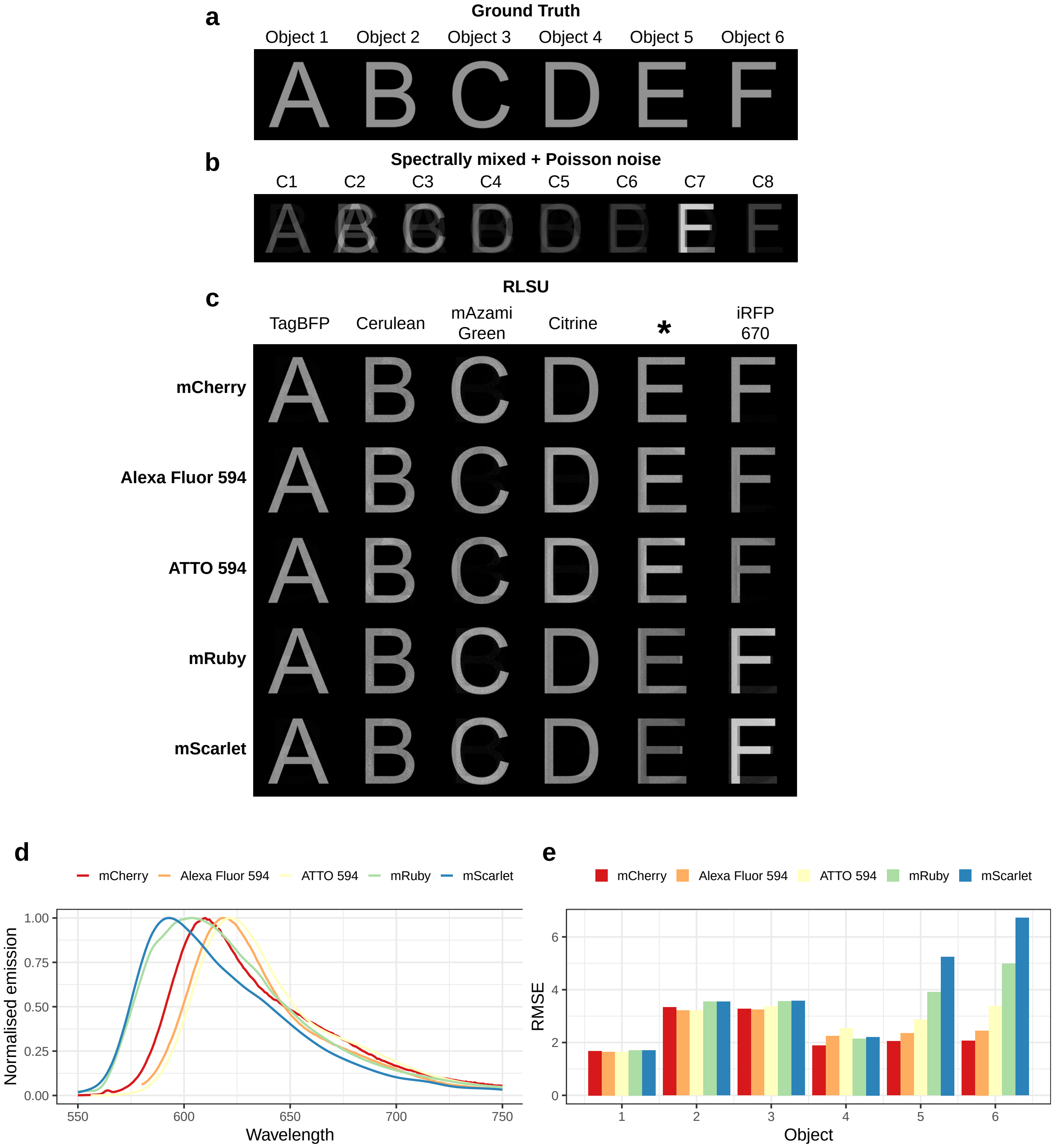

Supplementary Figure 15: Assessment of RLSU performance when using incorrect mixing matrices.
a Ground truth objects. **b** Spectrally mixed data with Poisson (shot) noise incorporated. Data was mixed by a mixing matrix related to the following fluorophores: TagBFP, Cerluean, mAzami Green, Citrine, mCherry and iRFP 670. **c** Data in (b) was unimixed using RLSU and different mixing matrices. The top row was reconstructed after unmixing with the correct mixing matrix. The other rows were reconstructed after unmixing with incorrect mixing matrices, where mCherry was substituted with Alexa Fluor 596, ATTO 594, mRuby and mScarlet, respectively. This was done to assess how well RLSU could reconstruct ground truth objects when unmixing with erroneous mixing matrices. **d** Emission spectra of mCherry and other fluorophores used to form mixing matrices to produce unmixed data in (b). **e** Root mean squared errors associated with reconstructing ground truth objects after unmixing with the correct mixing matrix, and erroneous mixing matrices where mCherry was substituted for other fluorophores.

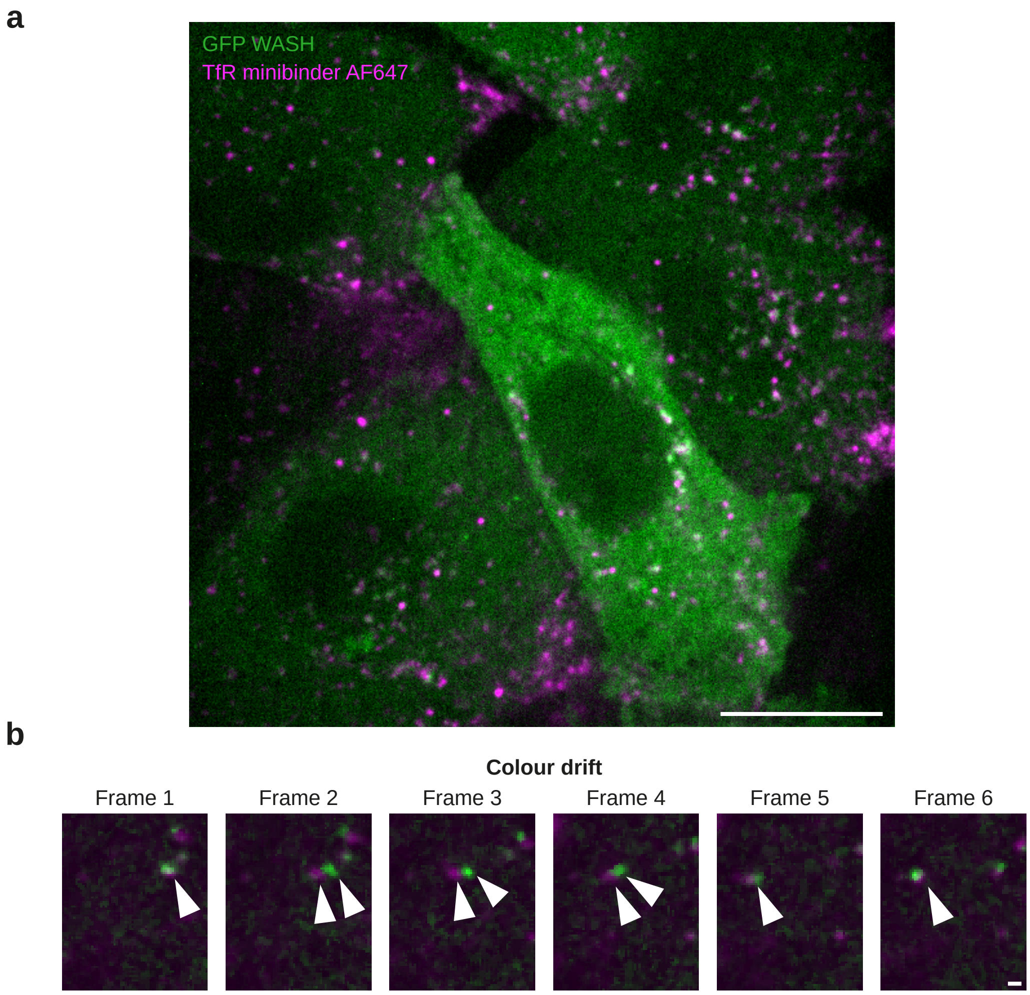

Supplementary Figure 16: Sequential two-colour live imaging of fast moving structures leads to colour drift artefacts.
a NIH 3T3 cells stably expressing EGFP-WASH and incubated with TfR minibinder Alexa Fluor 647 were imaged live by spinning disk confocal microscopy (single confocal plane shown). Scale bar = 10 µm. **b** Frames from the movie demonstrating colour drift artefacts, where the endosome has moved during filter-switching. This erroneously makes the green and magenta labels appear spatially distinct. Scale bar = 1 µm. Scale bars = 10 µm.

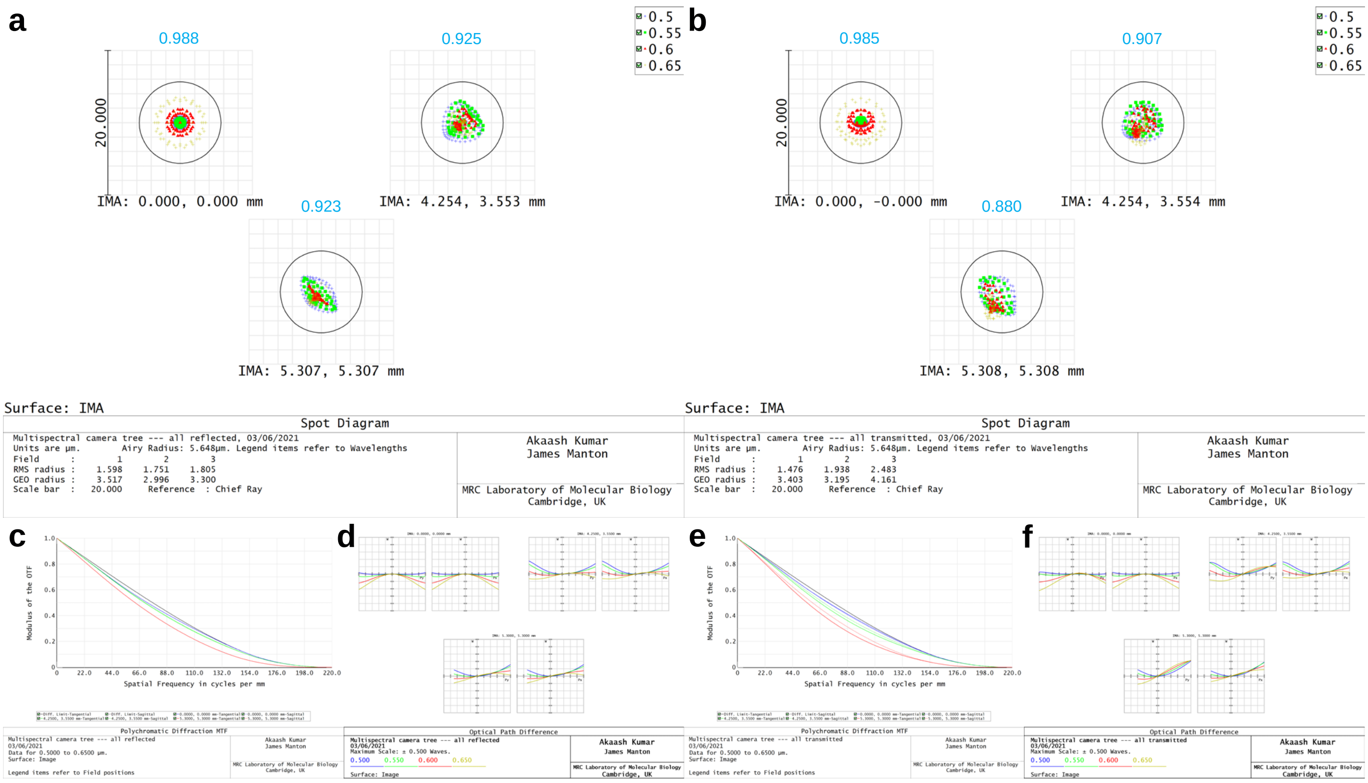

Supplementary Figure 17: Raytracing analysis of eight channel imaging hardware.
a–b Spot diagrams for central field, edge of camera chip and edge of tube lens image circle for (a) path in which light is reflected off all dichroics and (b) path in which light transmitted through all dichroics. Solid line shows Airy radius, demonstrating diffraction-limited performance across all fields for 500 nm to 650 nm. Cyan numbers indicate Strehl ratios. **c** Modulation transfer functions for reflected path. **d** Optical path difference plots for reflected path. **e** Modulation transfer functions for transmitted path. **f** Optical path difference plots for transmitted path.

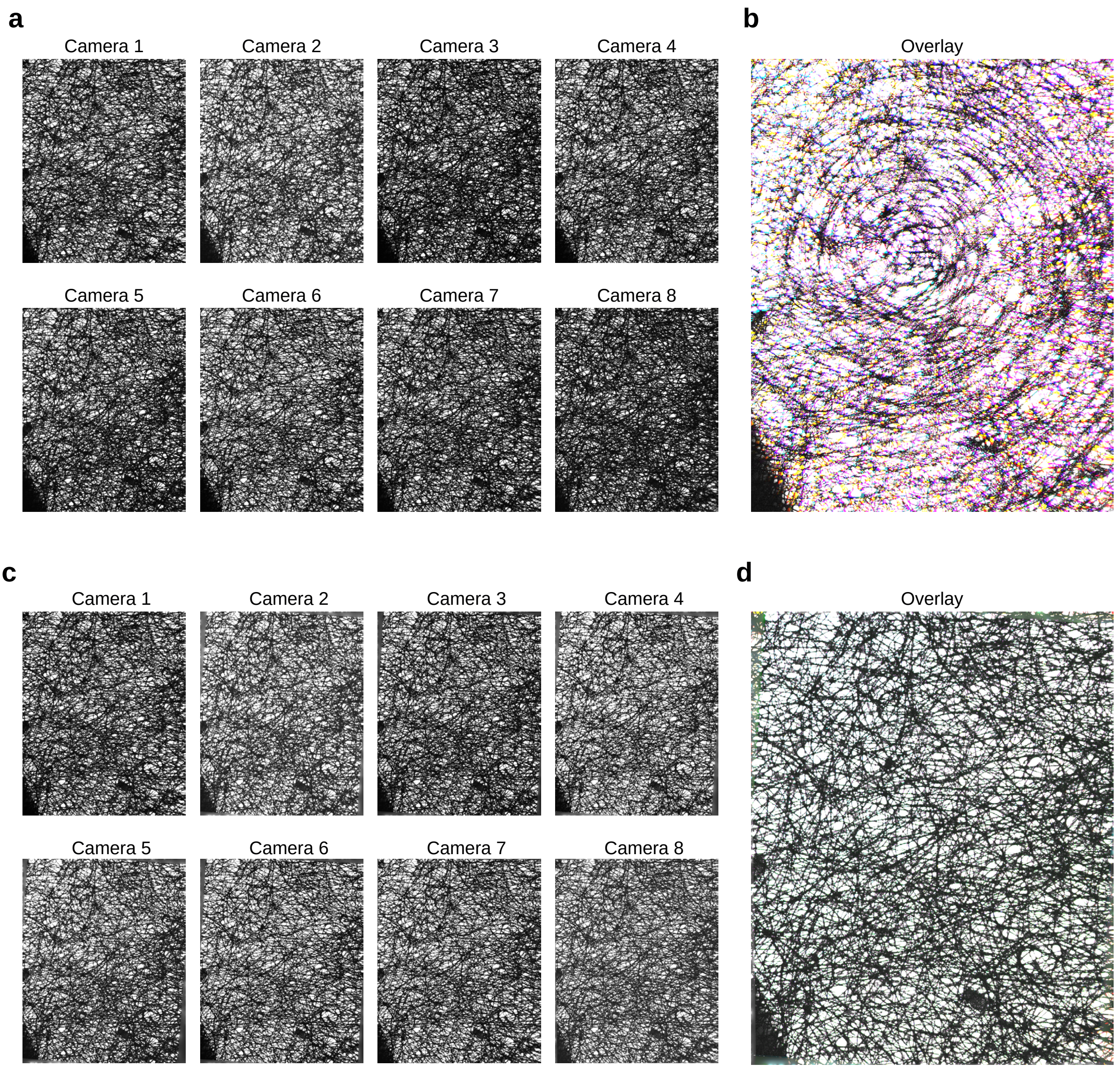

Supplementary Figure 18: Computational registration using lens tissue images.
a Images aquired by all 8 cameras of lens tissue placed in the microscope image plane. **b** Overlay of the data shown in a. **c** Data from (a), after computational registration. **d** Overlay of the data post computational registration.

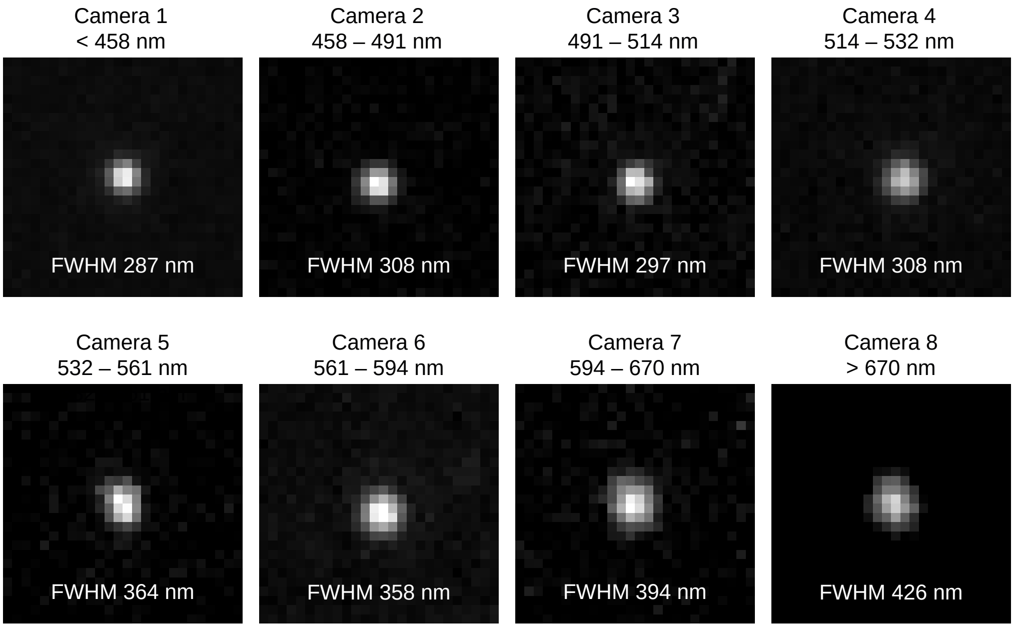

Supplementary Figure 19: Multispectral spinning disk microscope point spread functions.
Point spread functions and full-width-half-maximums displayed for the eight cameras on the multispectral spinning disk microscope used in this study. Pixel pitch = 98.5 nm/px.

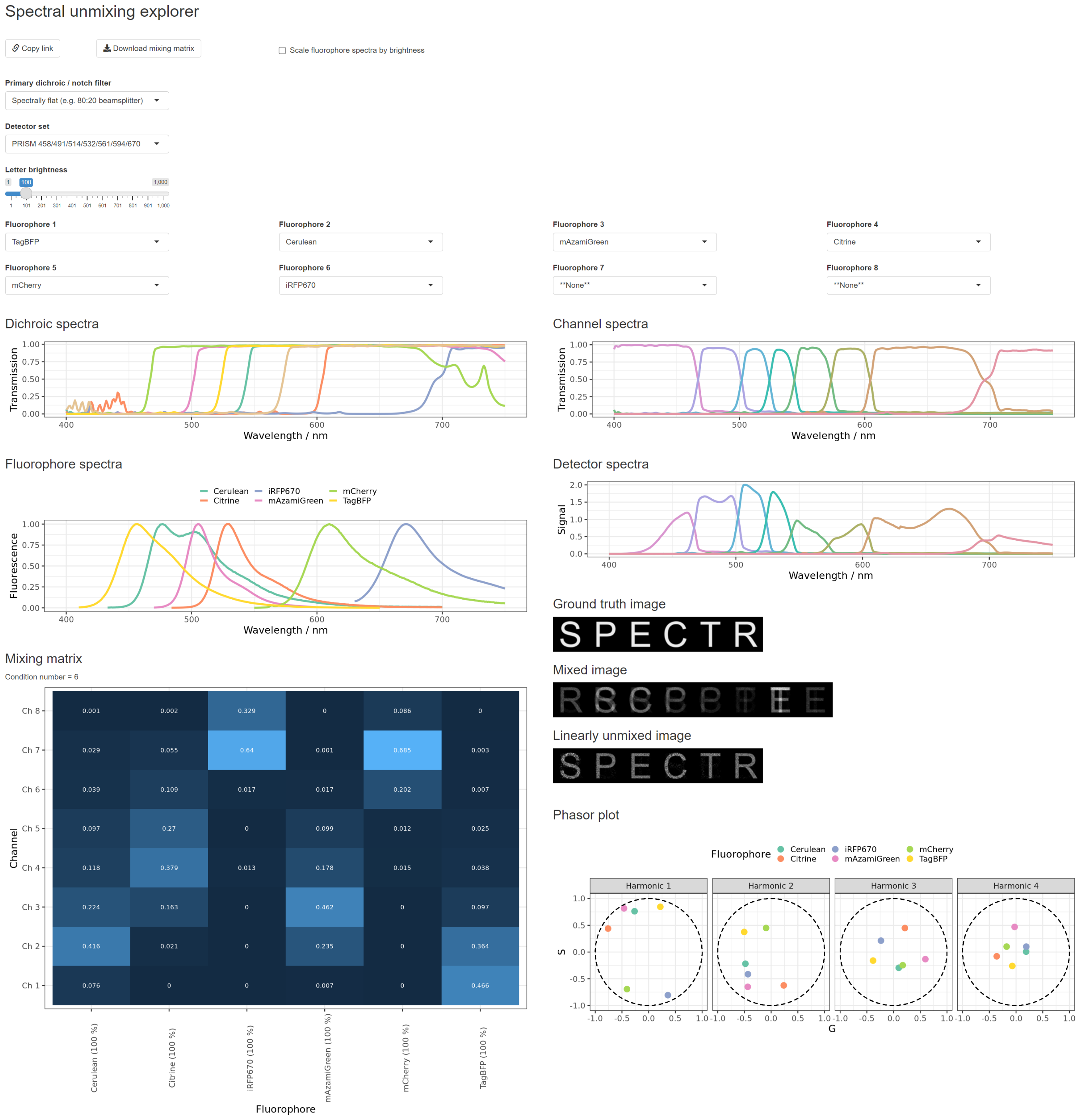

Supplementary Figure 20: Screenshot of web application developed to facilitate the choice of fluorophores and the computation of unmixing matrices.
Screenshot of web application hosted at beryl.mrc-lmb.cam.ac.uk/calculators/spectral_unmixing/. Users select fluorophores, using spectral information taken from fpbase.org, and select an appropriate dichroic set. The application then plots the relevant spectra, calculates a mixing matrix (shown as a blue heatmap) and produces simulated raw and linearly unmixed data (to the right of the heatmap). These simulated results, along with the condition number (printed under the mixing matrix heading) and phasor plots (bottom right) help the user check that their fluorophore and dichroic selection is appropriate. In particular, a small condition number, well-dispersed points on the phasor diagrams, and well-unmixed simulated data are desired. If the user wishes to share their selection with another user, a button is provided to generate a link which, when clicked, will preload the application with all the relevant data.

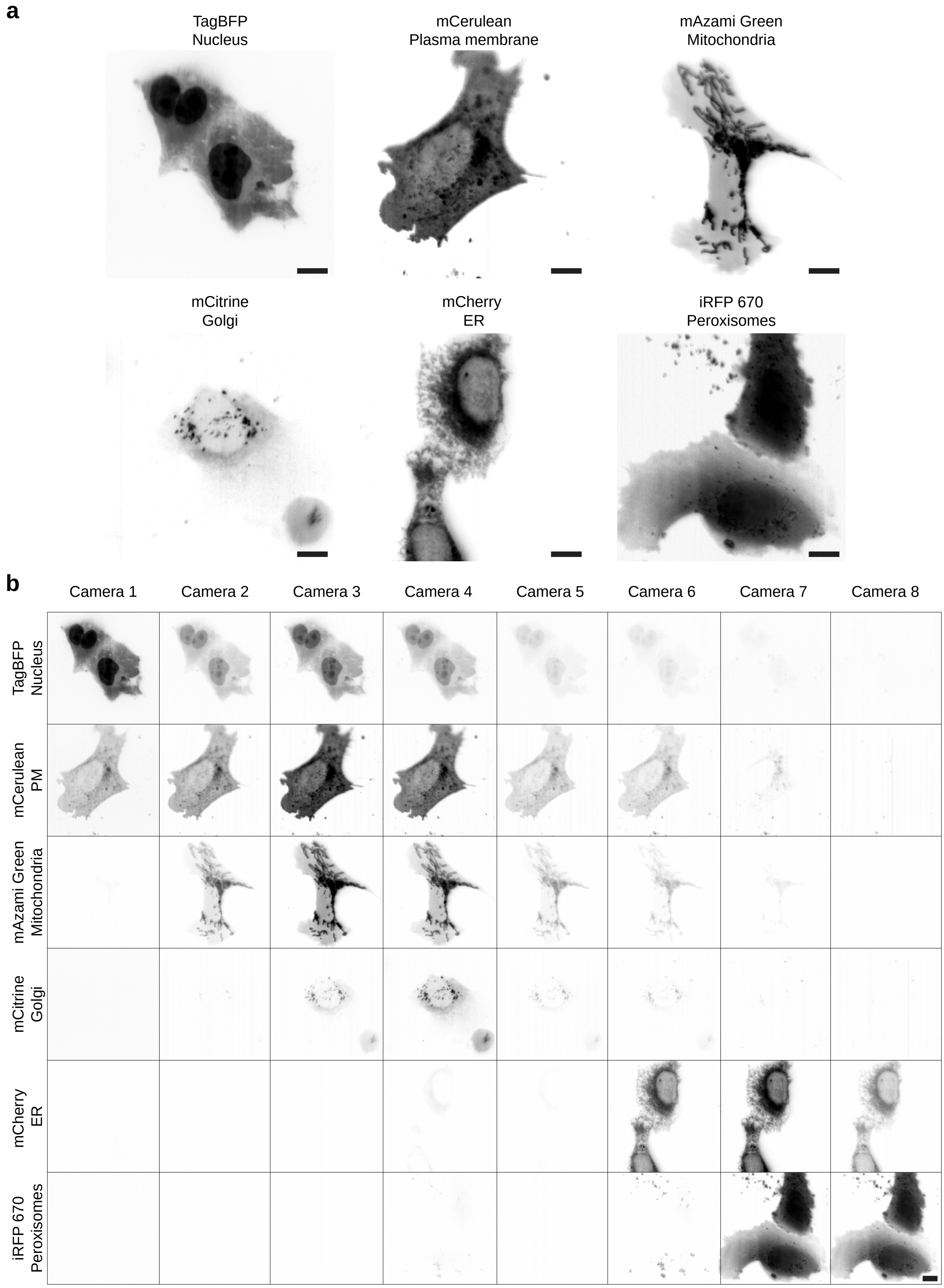

Supplementary Figure 21: Single-labelled controls of the individual fluorescent markers encoded by ColorfulCell.
a Maximum intensity projections of U2OS cells transfected with individual fluorescent markers present in the ColorfulCell plasmid. This provides definitive examples of what each of the components should look like post-unmixing. Data acquired using the multispectral OPM followed by registering and summing the data from the eight channels (summed intensity projection). **b** Signals across all 8 cameras are shown for each of the cells presented in (a). These signals can be measured for each marker to determine a measured mixing matrix for subsequent RLSU unmixing of ColorfulCell data. Scale bars = 10 µm.

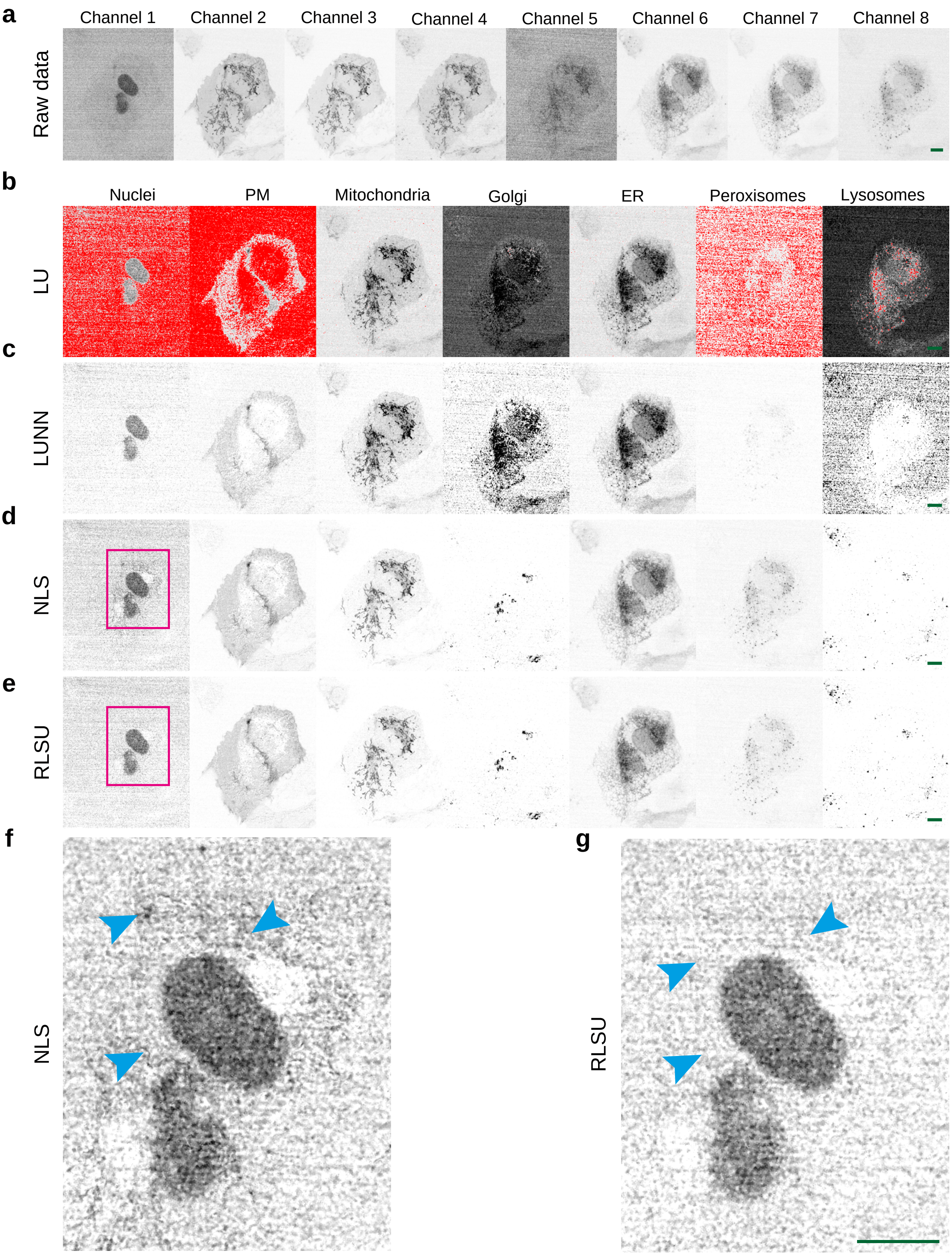

Supplementary Figure 22: Comparison of RLSU, linear unmixing and non-negative least squares (NLS) for unmixing live-cell multi-spectral data.
a Raw spectrally mixed data. Unmixed object reconstructions of organelle targets using **b** LU, **c** retaining non-negative pixel values only from the LU results, **d** using NLS and **e** RLSU. Zoom of unmixed nuclei reconstructions using **f** NLS and **g** RLSU corresponding with magenta boxes in (d) and (e). Cyan arrows indicate erroneous residual signal unrelated to the nuclei present in the unmixed NLS result but appropriately absent from the RLSU result. PM = plasma membrane, ER = endoplasmic reticulum. Negative pixel values are shown in red, an inverted LUT is used to display the data. Scale bars = 10 µm.

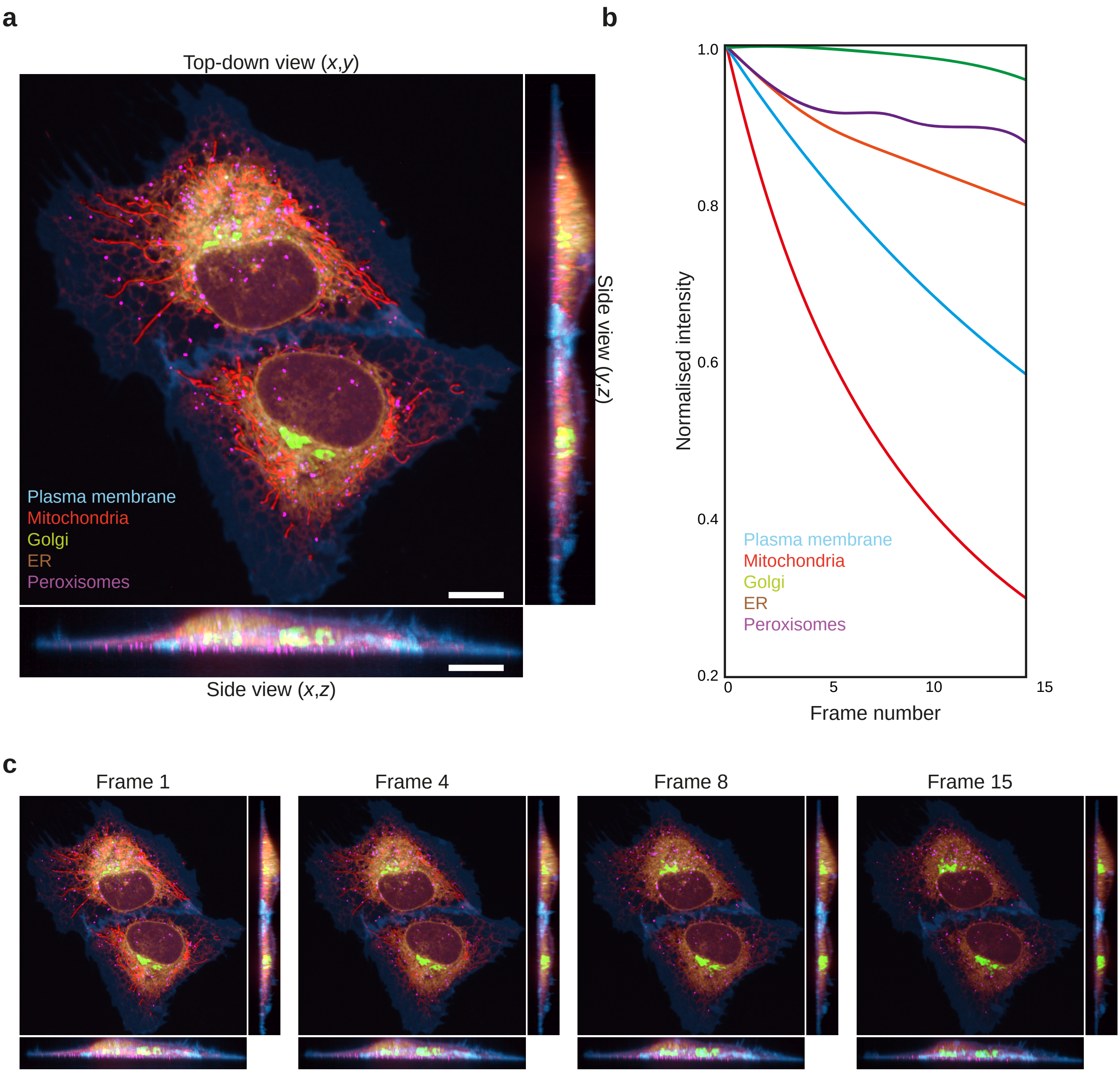

Supplementary Figure 23: Multispectral spinning disk confocal microscope causes photodamage during fast volumetric acquisitions. **a** Live U2OS cells transfected with the ColorfulCell plasmid were imaged by multispectral confocal spinning disk followed by RLSU unmixing. Images represent orthogonal maximum intensity projections of confocal volumes in indicated axis (51 planes ; 200 nm increment). **b** Normalised intensity over time for signals in shown in (a). **c** Timelapse volumetric aquisition showing progressive signal bleaching and cellular photodamage (*e.g.* cell shrinking). see also Supplementary Video 6.

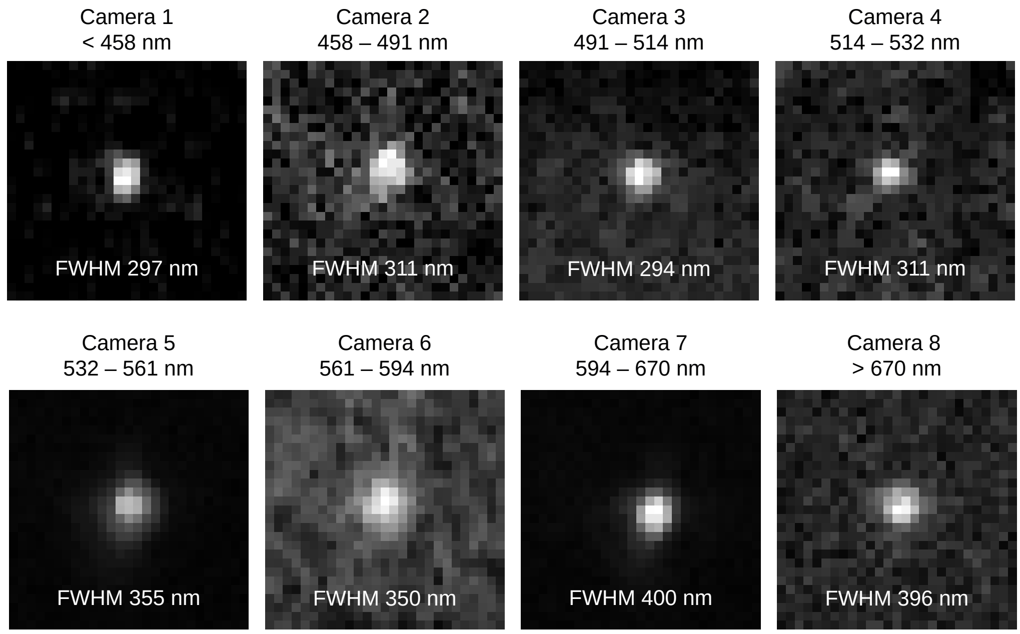

Supplementary Figure 24: Multispectral oblique plane light sheet microscope point spread functions.
Point spread functions and full-width-half-maximums displayed for the eight cameras on the multispectral oblique plane light sheet microscope. Pixel pitch = 111.6 nm/px.

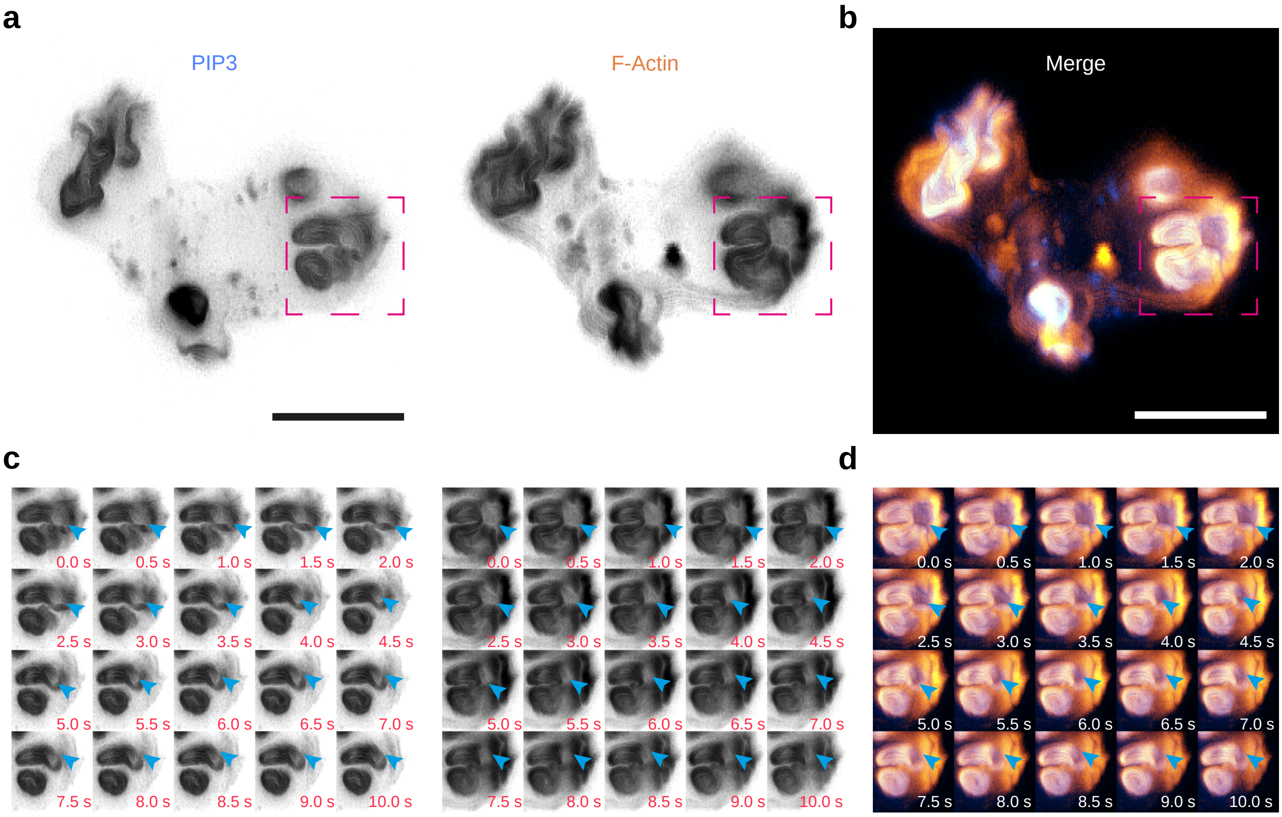

Supplementary Figure 25: Fast volumetric imaging of live *Dictyostelium discoideum* cells.
a Maximum intensity projections of live *Dictyostelium discoideum* cell expressing EGFP-fused to a PIP3 reporter and LifeAct-mCherry. Data acquired using the multispectral oblique plane light sheet microscope at 2 Hz. See also Supplementary Video 11. **b** Colour merge of data in (a). **c** Frames from timelapse (cyan dashed box in (a)) showing macropynocitic cup closure. Time interval 0.5 s between frames. **d** Colour merge of (c). Scale bars = 10 μm.

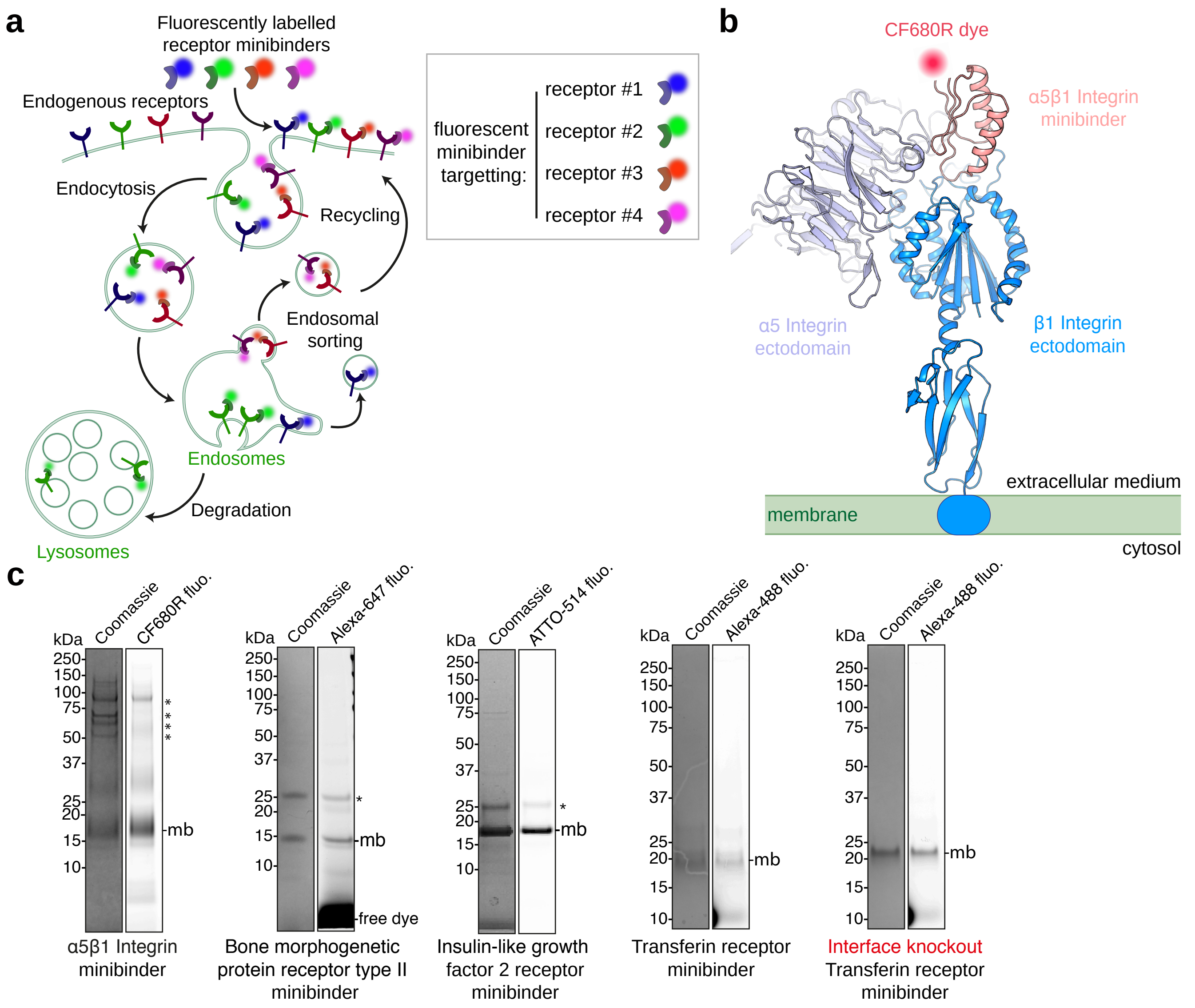

Supplementary Figure 26: Biochemical characterisation of de novo designed receptor minibinders.

a Principle of the experiment. Cells are incubated with a panel of de novo designed fluorescent minibinders targetting different endogenous receptors on ice to block endocytosis. After 30 minutes, cells are washed extensively and incubated with warm serum-containing medium to trigger endocytosis of the receptors bound to their cognate fluorescent minibinders. The fluorescence signal of the different minibinders can then be used to directly image the sorting of the different receptors into different organelles, for instance between early/sorting endosomes and late endosomes. Different organelles are expected to gradually have different ratios of the different minibinders if sorting occurs. On the contrary, if no sorting occurs, or if the minibinders enter the cell non-specifically by fluid-phase endocytosis, we expect all compartments to consistently have the same ratios of the different minibinders. **b** Example of de novo designed fluorescent minibinder against integrin $\alpha5\beta1$. Note that the minibinder is monovalent, so does not dimerise the receptor, and that the reactive cysteine used to functionalise the minibinder with the organic dye CF680R is positioned away from the surface binding to the receptor. **c** SDS-PAGE gel of indicated receptor minibinder followed showing in-gel fluorescence and Coomassie blue staining. Asterisk indicates contaminants (usually leftover GST from the purification).

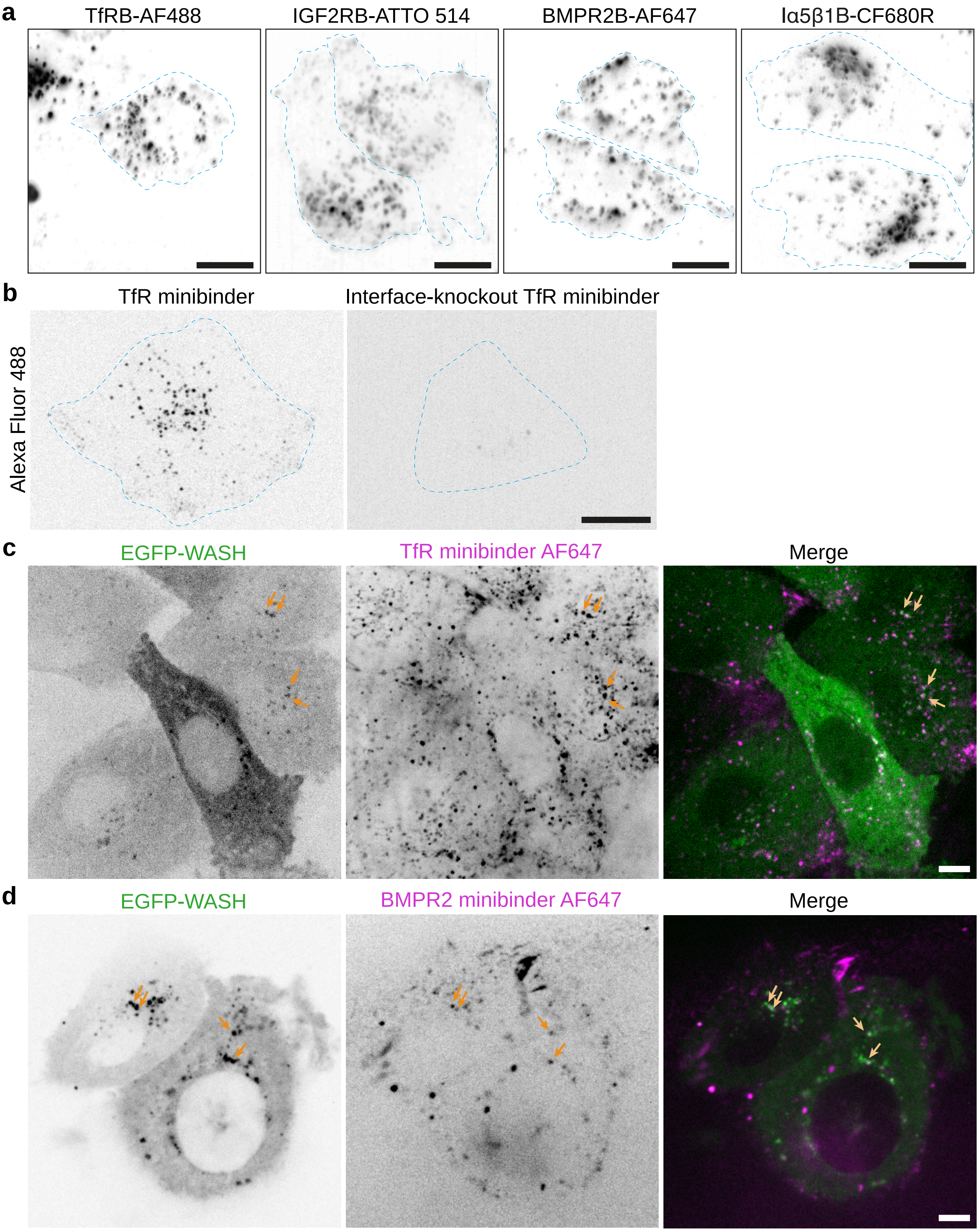

Supplementary Figure 27: Validation of de novo designed receptor minibinders for imaging of receptor trafficking in live cells.
a HeLa Kyoto cells incubated with indicated fluorescent minibinders (cyan dashed line indicates cell boundaries). Images correspond to maximum intensity projections from multispectral OPM data. This is a control related to Figure 6a showing all four fluorescent minibinders can be uptaken by cells individually. **b** HeLa Kyoto cells incubated with Alexa Fluor 488 labelled, TfR minibinder (left) or a respective interface knockout mutant (right), were imaged live with spinning disk confocal microscopy. Images correspond to single confocal planes (cyan dashed line indicates cell boundaries). Note that no signal is observed for the interface knockout mutant [24], suggesting that the fluorescent signal observed in the wildtype minibinder corresponds to minibinder bound to its target transferrin receptor, and not non-specific fluid phase uptake of the fluorescent minibinder. **c** NIH 3T3 cells stably expressing EGFP-WASH and incubated with TfR minibinder Alexa Fluor 647 were imaged live by spinning disk confocal microscopy (single confocal plane shown). Note the colocalisation between EGFP and Alexa Fluor 647 (arrow) suggesting that the TfR minibinder is trafficking through WASH-positive early/sorting endosomes. **d** NIH 3T3 cells stably expressing EGFP-WASH and incubated with BMPR2 binder Alexa Fluor 647 were imaged live by spinning disk confocal microscopy (single confocal plane shown). Note the colocalisation between EGFP and Alexa Fluor 647 (arrow) suggesting that the BMPR2 minibinder is trafficking through WASH-positive early/sorting endosomes. Scale bars = 10 µm.

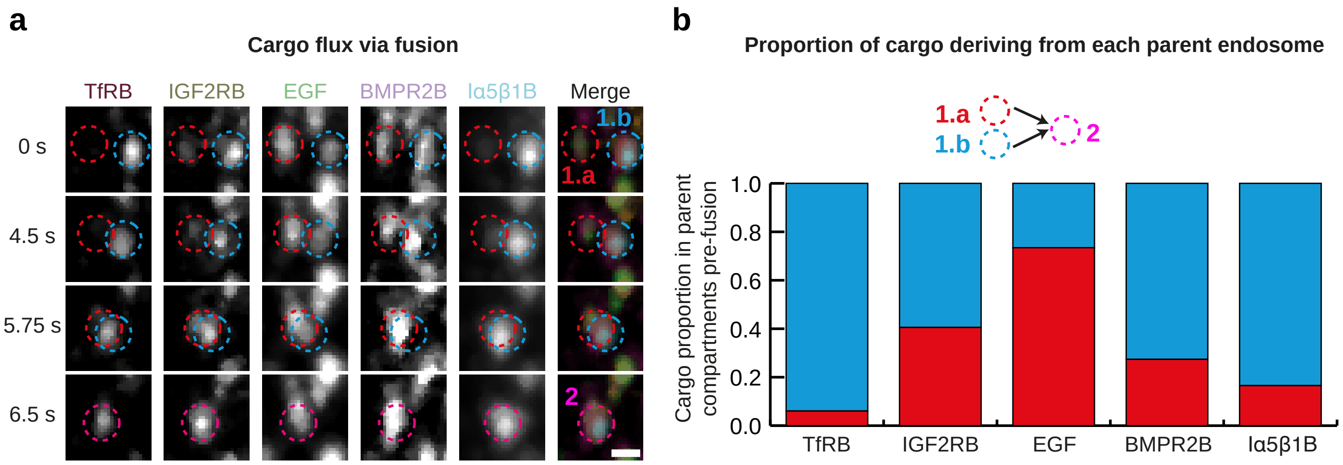

Supplementary Figure 28: Multispectral light sheet imaging of endosomal fusion using de novo designed receptor minibinders.
a Frames from Supplementary Video 14 showing cargo flux during endosomal fusion. A maximum intensity projection is shown. Individual channels and colour merge are shown. Red and cyan dashed circles indicate two endosomes, 1.a and 1.b respectively, which fuse to form endosome 2, indicated by a magenta dashed circle. Scale bar = 1 µm. **b** Quantification of the event shown in (a) to show the proportion of cargo in endosome 2 deriving from endosomes 1.a and 1.b.

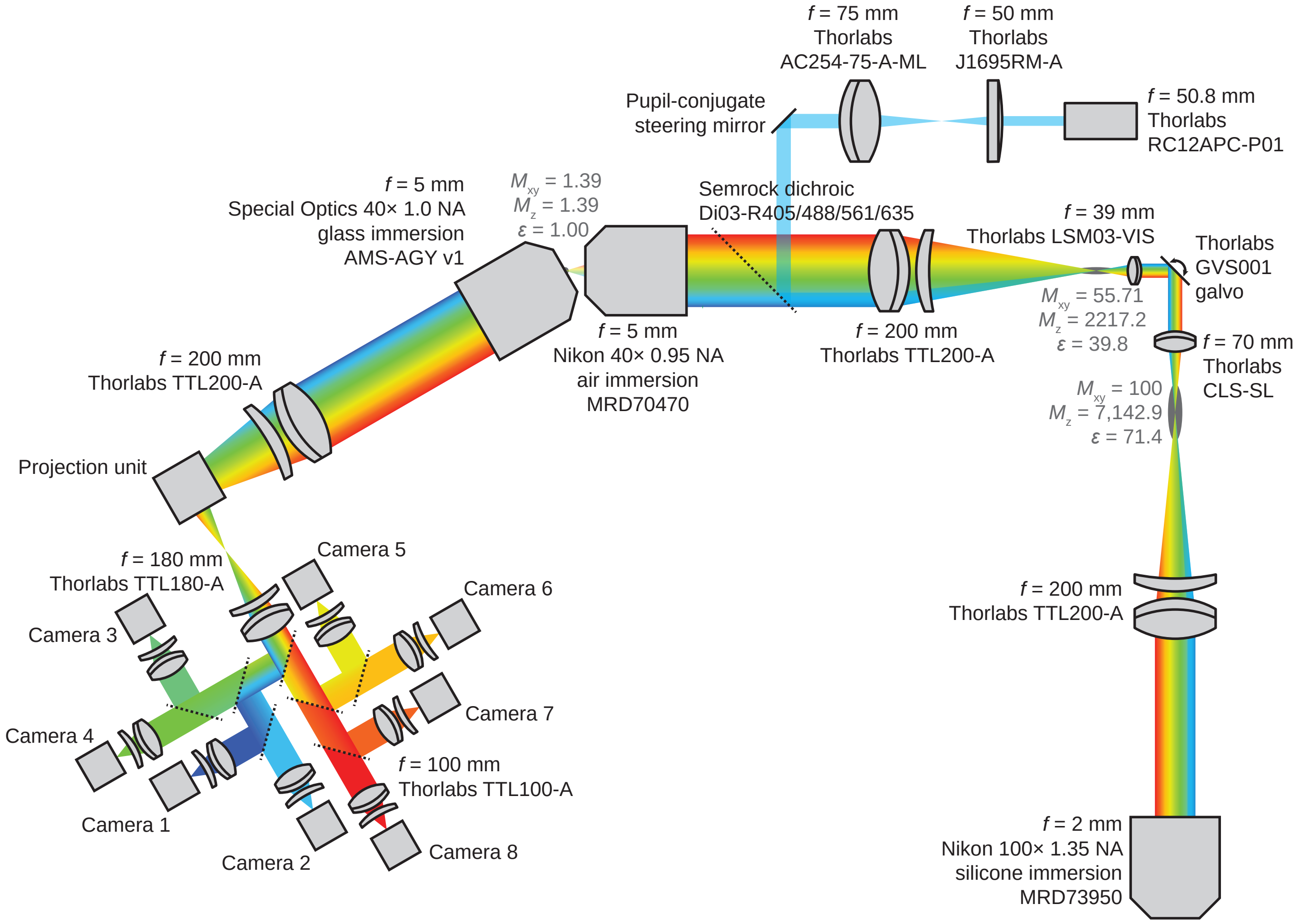

Supplementary Figure 29: Optical diagram of the multispectral OPM.
Fluorescence enters the 100× 1.35 NA objective (bottom right) and is directed through a tube lens and scan lens onto a galvanometric mirror, which scans the image plane (and light sheet) through the sample. This light then passes through another scan lens, tube lens and 40× 0.95 NA objective lens into the remote refocussing volume. Here, both the lateral (M_{xy}) and axial magnifications (M_z) are set to be equal ($\epsilon = 1.00$), and approximately equal to the refractive index of the sample, 1.39. This ensures that an aberration-free 3D volume is formed, a tilted plane of which is then imaged by the solid-immersion 40× 1.0 NA lens and tube lens onto the input plane of the multispectral camera system. A projection unit, consisting of another galvanometric mirror synchronised to the one moving the light sheet and image plane, is inserted just before the input image plane of the multispectral camera system to facilitate projection imaging. For illumination, laser light is directed from a reflective collimator through a cylindrical lens and achromatic doublet onto a dichroic mirror which couples the light into the main optical path, propagating in the opposite direction to the fluorescence light. A pupil-conjugate steering mirror facilitates alignment of the sheet of illumination to the tilted focal plane of the instrument.

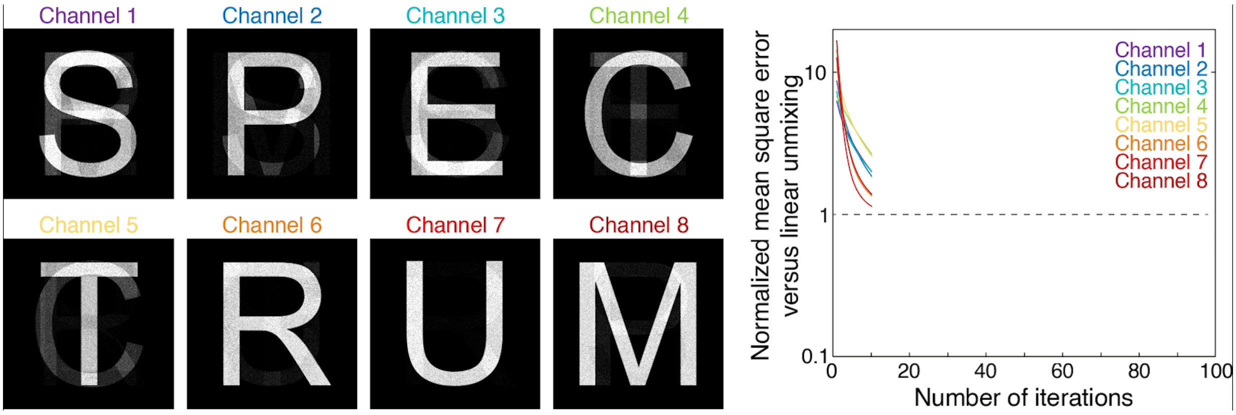

Supplementary Video 1: RLSU outperforms linear unmixing with simulated multispectral datasets.
Relates to Supplementary Figure 10. Eight simulated ground truth objects were spectrally mixed and Poisson shot noise was incorporated to simulate eight-channel multispectral fluorescence microscopy data. These images were unmixed using RLSU. Left panel shows how the data progressively gets unmixed as the number of iteration increases. Right panel shows the normalised mean squared error of unmixing for RLSU iterations versus linear unmixing for the data presented on the left panel Unmixing mean squared error shown for all 8 channels in RLSU in lines colour-coded by channel, whilst the linear unmixing result is indicated by dashed horizontal black line.

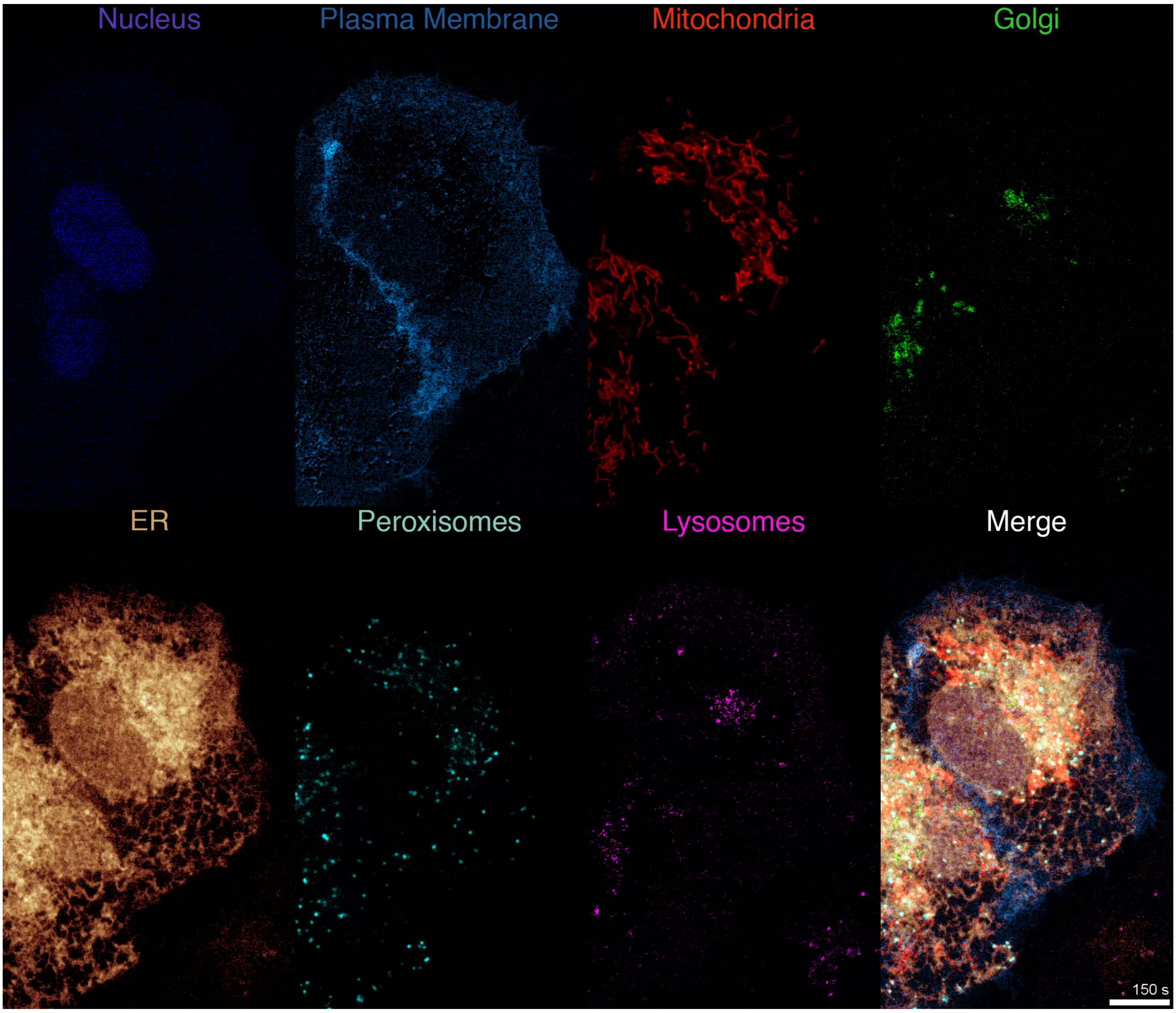

Supplementary Video 2: Multispectral spinning disk confocal imaging of organelle trafficking.
Relates to Figure 3d. U2OS cells transfected with the ColorfulCell plasmid were incubated with LysoTracker Yellow before imaging on a confocal spinning disk instrument equipped with the multispectral camera unit followed by RLSU unmixing. Scale bar = 10 µm.

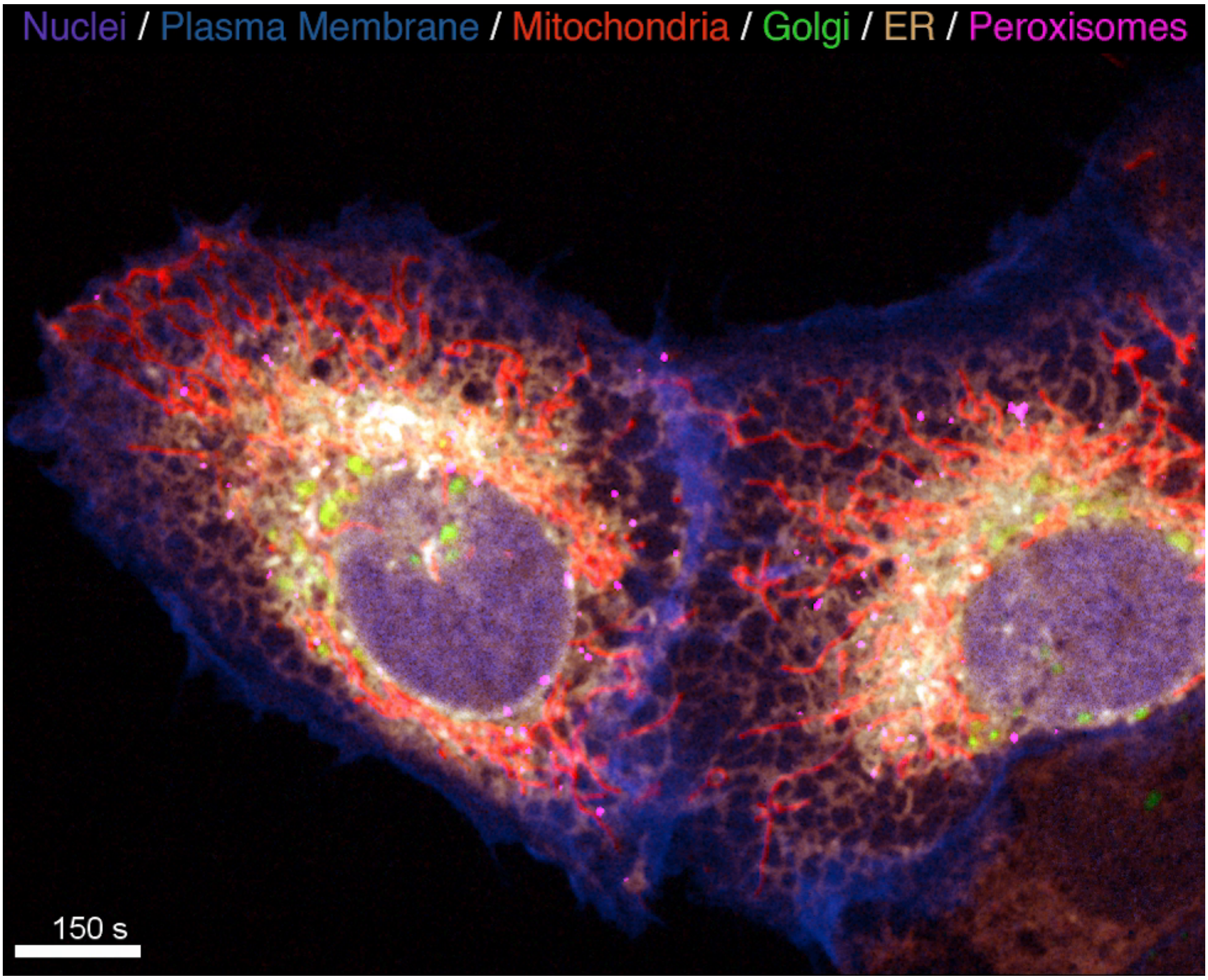

Supplementary Video 3: Multispectral spinning disk confocal imaging.
Relates to Figure 3e. U2OS cells transfected with the ColorfulCell plasmid were imaged on a confocal spinning disk instrument equipped with the multispectral camera unit followed by RLSU unmixing. Scale bar = 10 µm.

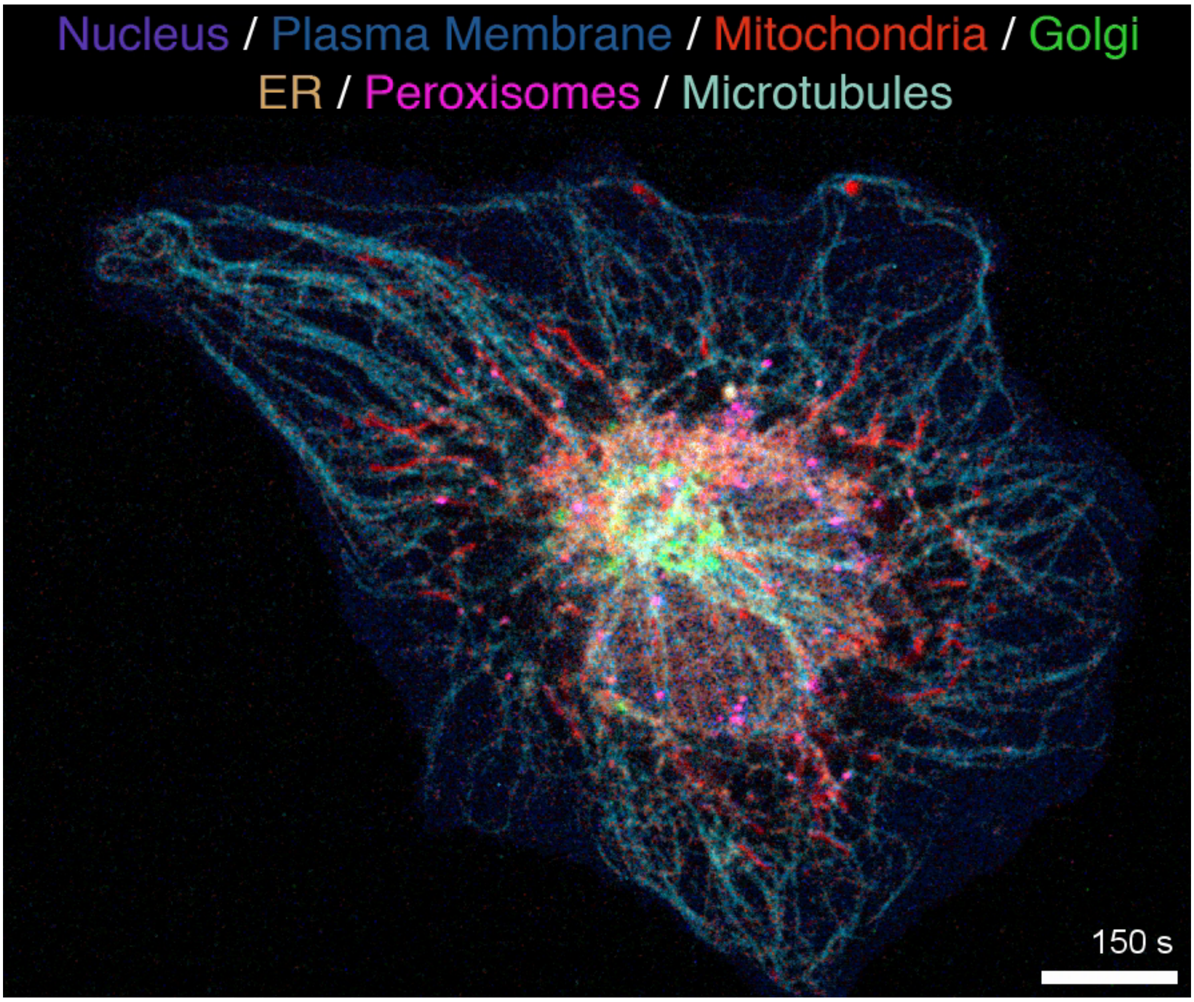

Supplementary Video 4: Multispectral spinning disk confocal imaging of microtubules and various organelles.
Relates to Figure 3f. U2OS cells transfected with the ColorfulCell plasmid and incubated with SPY555-tubulin were imaged on a confocal spinning disk instrument equipped with the multispectral camera unit followed by RLSU unmixing. Scale bar = 10 µm.

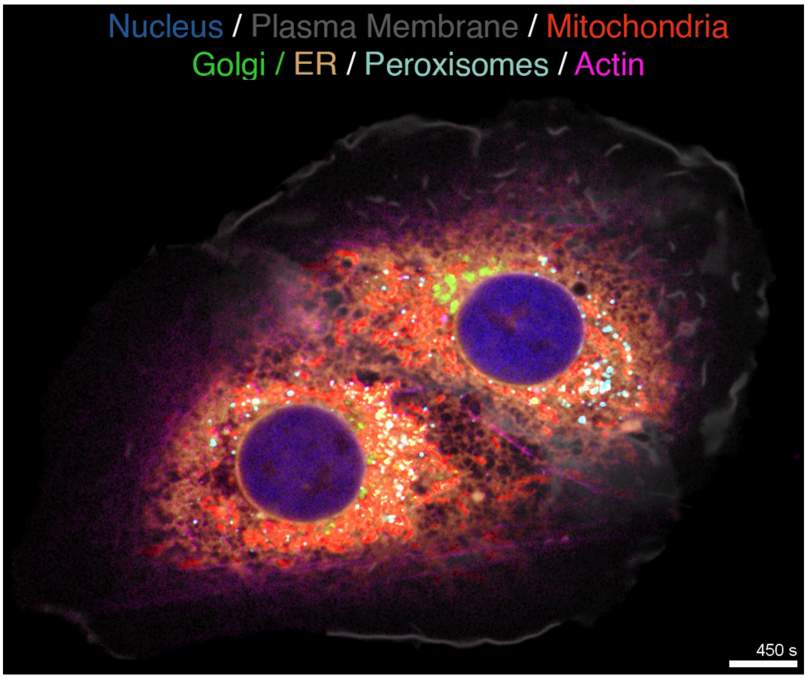

Supplementary Video 5: Multispectral spinning disk confocal imaging of actin and various organelles.
Relates to Figure 3g. U2OS cells transfected with the ColorfulCell plasmid and incubated with SPY555-actin were imaged on a confocal spinning disk instrument equipped with the multispectral camera unit followed by RLSU unmixing. Scale bar = 10 µm.

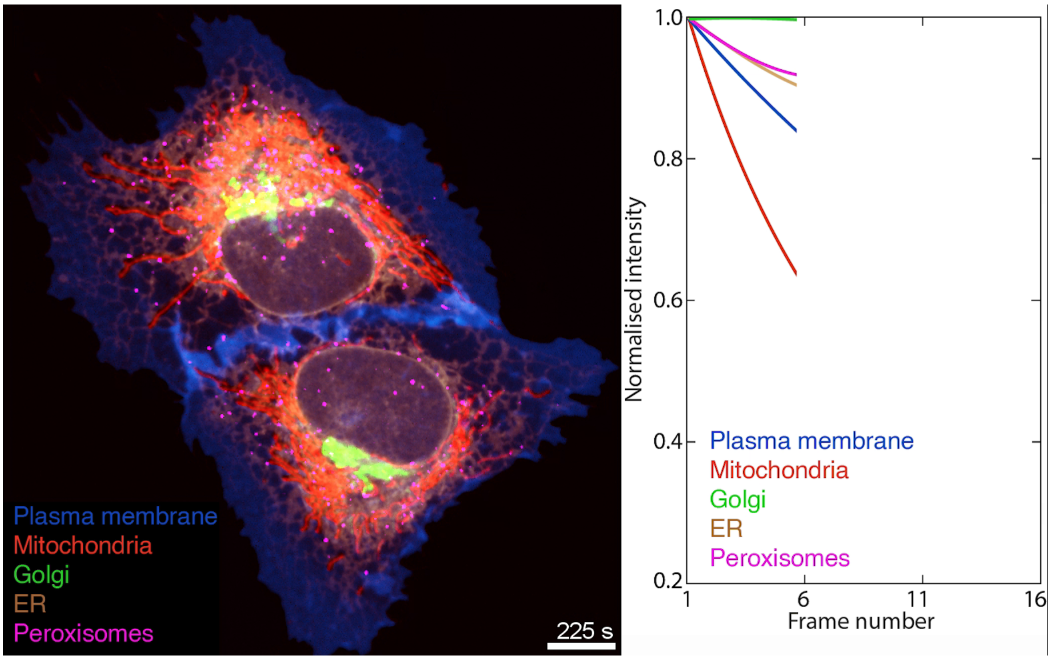

Supplementary Video 6: Volumetric multispectral spinning disk confocal microscopy exhibits significant photobleaching.
Relates to Supplementary Figure 23. Left panel: U2OS cells transfected with the ColorfulCell plasmid were imaged on a confocal spinning disk instrument equipped with the multispectral camera unit followed by RLSU unmixing. Images represent orthogonal maximum intensity projections of confocal volumes (51 planes ; 200 nm increment). Right panel: normalised intensity over time of left panel, showing significant photobleaching over time. Scale bar = 10 µm.

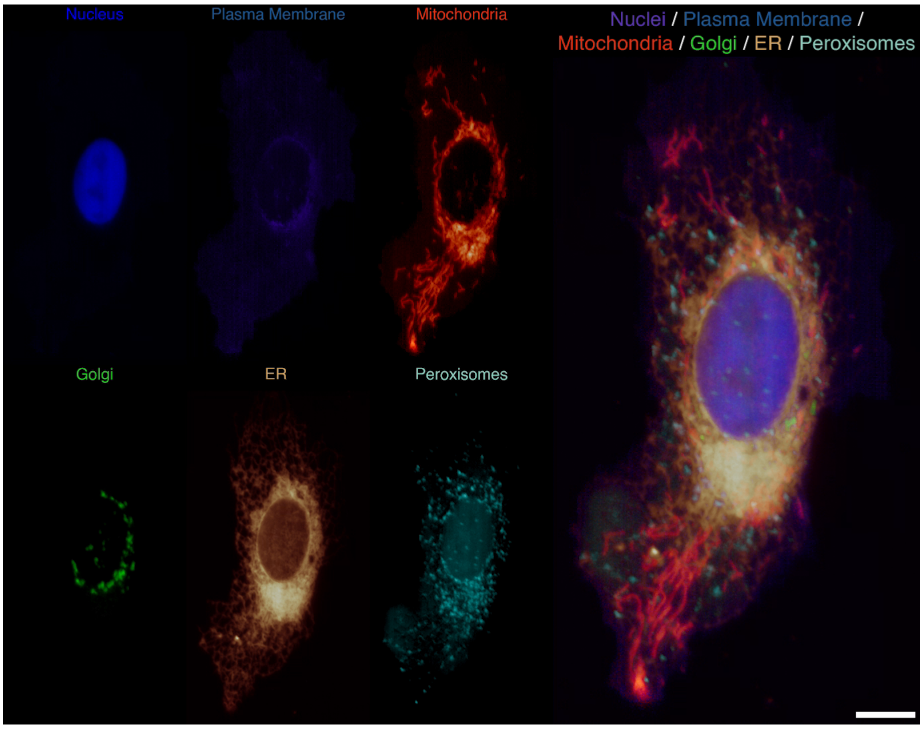

Supplementary Video 7: Multispectral volumetric light sheet imaging.
Relates to Figure 4. U2OS cells transfected with the ColorfulCell plasmid were imaged on an oblique plane light sheet instrument equipped with the multispectral camera unit followed by RLSU unmixing. movie show full cell volume (201 planes). Scale bar = 10 µm.

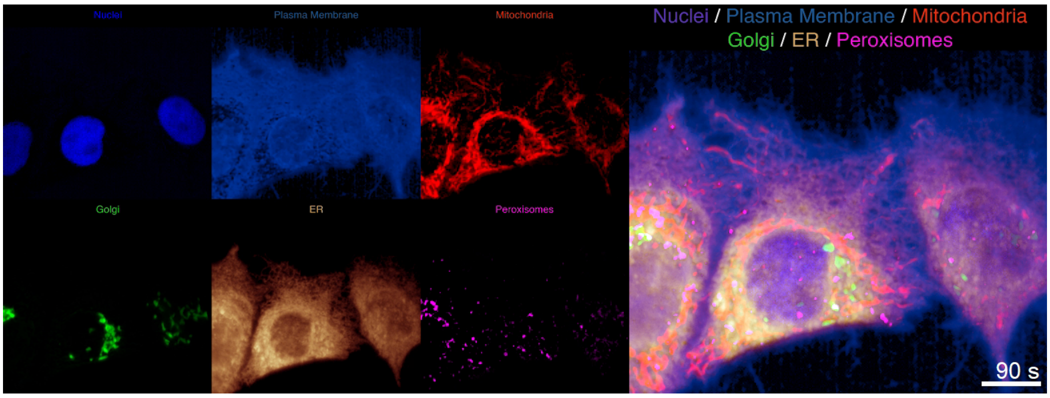

Supplementary Video 8: Multispectral volumetric light sheet imaging timelapse.
Relates to Figure 4. HeLa cells transfected with the ColorfulCell plasmid were imaged on an oblique plane light sheet instrument equipped with the multispectral camera unit followed by RLSU unmixing. Movie shows maximum intensity projections of full cell volumes (81 planes) over time. Scale bar = 10 µm.

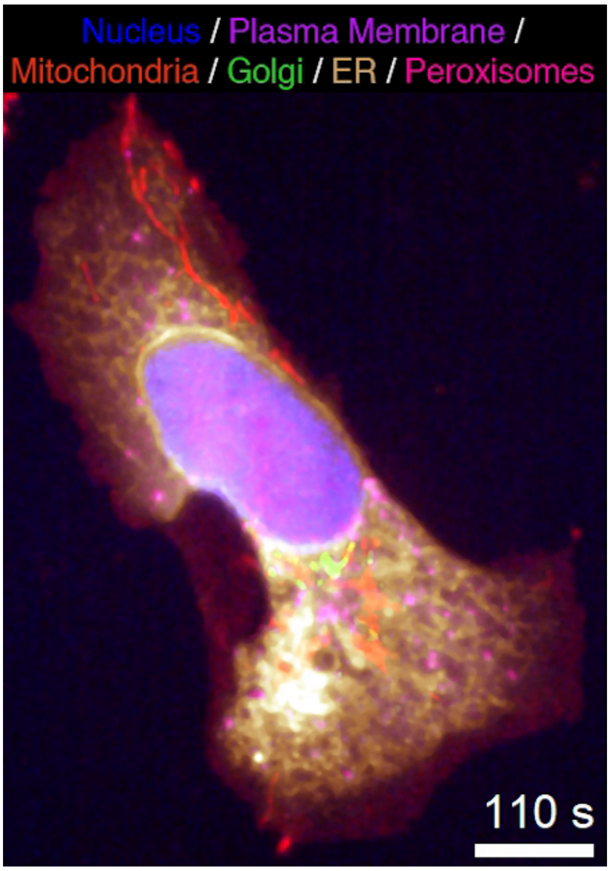

Supplementary Video 9: Long term, volumetric imaging by multispectral oblique plane light sheet microscopy.
Relates to Figure 4. U2OS cells transfected with the ColorfulCell plasmid were imaged on a oblique plane light sheet instrument equipped with the multispectral camera unit followed by RLSU unmixing. Movie shows maximum intensity projections of full cell volumes (101 planes) over time. Note the negligible photobleaching observed over the 200 timepoint timelapse (1 volume every 10 s) due to the gentle excitation strategy of the oblique plane light sheet microscope. Scale bar = 10 µm.

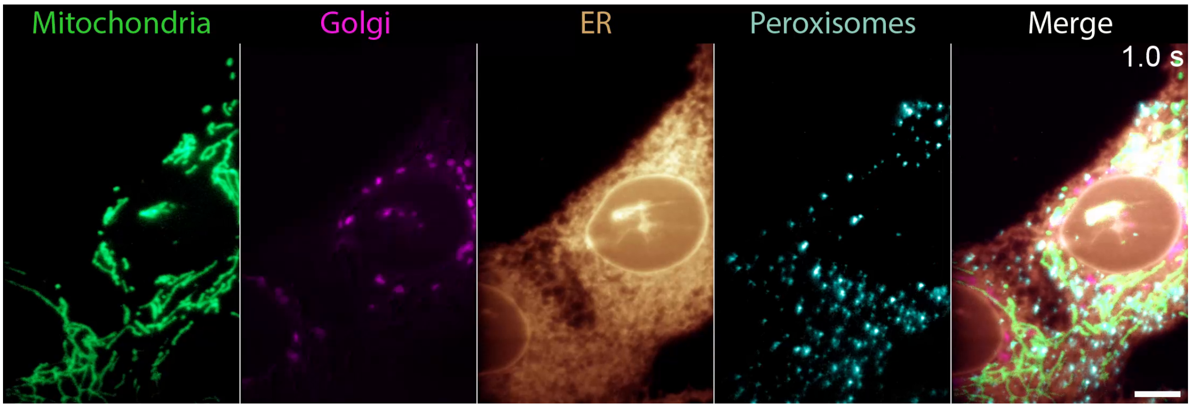

Supplementary Video 10: Fast multispectral live-cell imaging by projection light sheet microscopy.
Relates to Figure 5. U2OS cells transfected with the ColorfulCell plasmid were imaged by projection imaging on an oblique plane light sheet instrument equipped with the multispectral camera unit followed by RLSU unmixing. Only four markers were imaged (mAzamiGreen-mitochondria, Citrine-Golgi, mCherry-endoplasmic reticulum and iRFP670-peroxisomes) as 405 nm laser was not turned on in this specific experiment. Note how the fast framerate reached by the instrument (10 projections of the entire cells per second) allows one to simultaneously observe fast dyanmics (ER tubules) and slow dynamics (peroxisome Brownian motion). Scale bar = 10 µm.

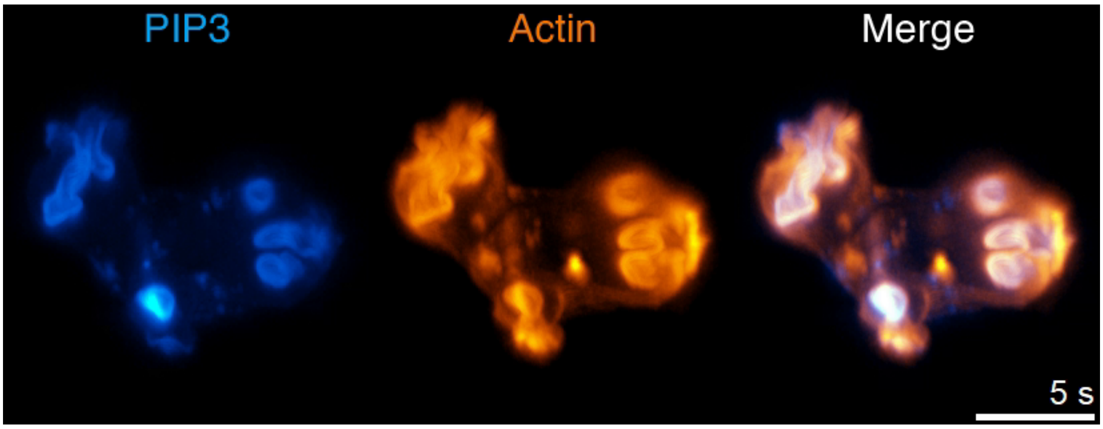

Supplementary Video 11: Fast volumetric imaging of live *Dictyostelium discoideum* cells.
Relates to Supplementary Figure 25. Live *Dictyostelium discoideum* cell expressing EGFP-fused to a PIP3 reporter and LifeAct-mCherry were imaged using the multispectral OPM at 2 volumes per second. Images correspond to maximum intensity projections. Note that this volumetric aquisition speed is enough to resolve macropinocytic cup closure. Scale bar = 10 µm.

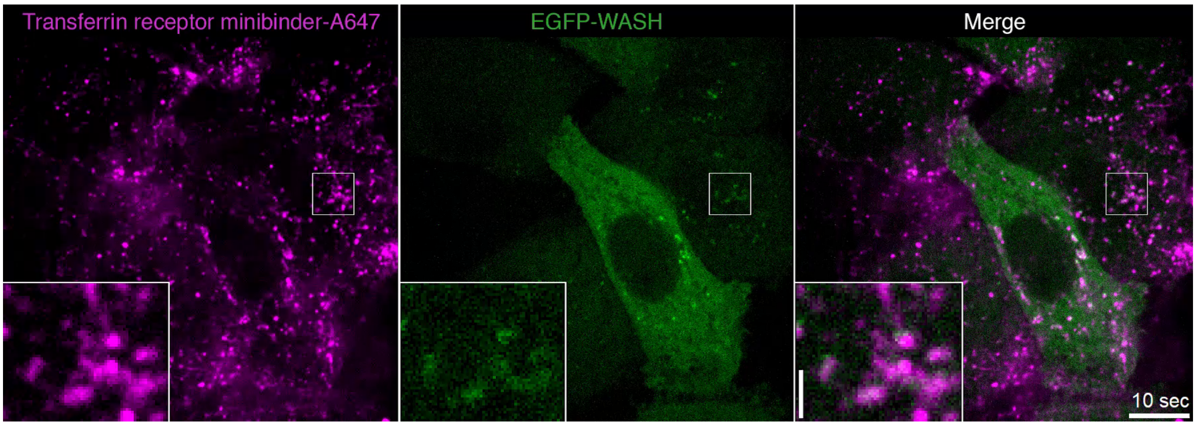

Supplementary Video 12: Validation that de novo designed binders reach early-sorting endosomes in live cells.
Relates to Supplementary Figure 27. NIH 3T3 cells stably expressing EGFP-WASH and incubated with Alexa Fluor 647-labelled TfR minibinder were imaged live by spinning disk confocal microscopy. Images correspond to single confocal planes. Scale bar = 10 µm.

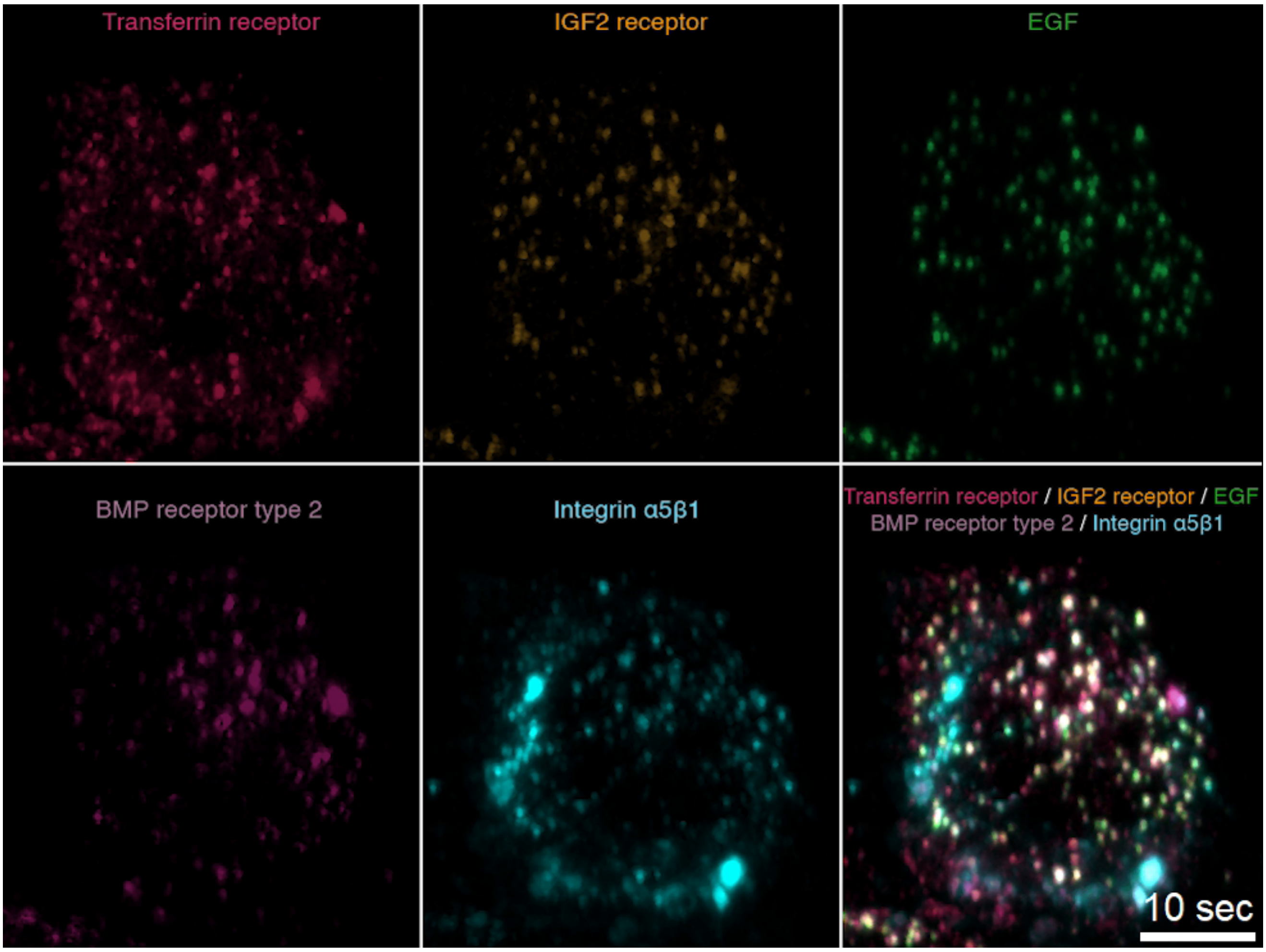

Supplementary Video 13: Multispectral live-cell light sheet imaging of intracellular trafficking using de-novo designed receptor minibinders.
Relates to Figure 6a. TagBFP-NLS-expressing HeLa Kyoto cell loaded with four fluorescently-labelled, computationally designed minibinders against cell surface receptors (TfR, IGF2R, BMPR2 & I$\alpha 5\beta 1$) and one fluorescently-labelled receptor ligand (EGF) were imaged live by multispectral oblique plane light sheet microscopy. Images correspond to maximum intensity projections of the entire cell volume (67 planes), and volumetric imaging is performed at 1 Hz. For convenience, the signal of TagBFP-NLS is not shown. Note that the ratio of the different minibinders and EGF is not the same in all endosomes, suggesting the different receptors are being sorted away from one another. Scale bar = 10 µm.

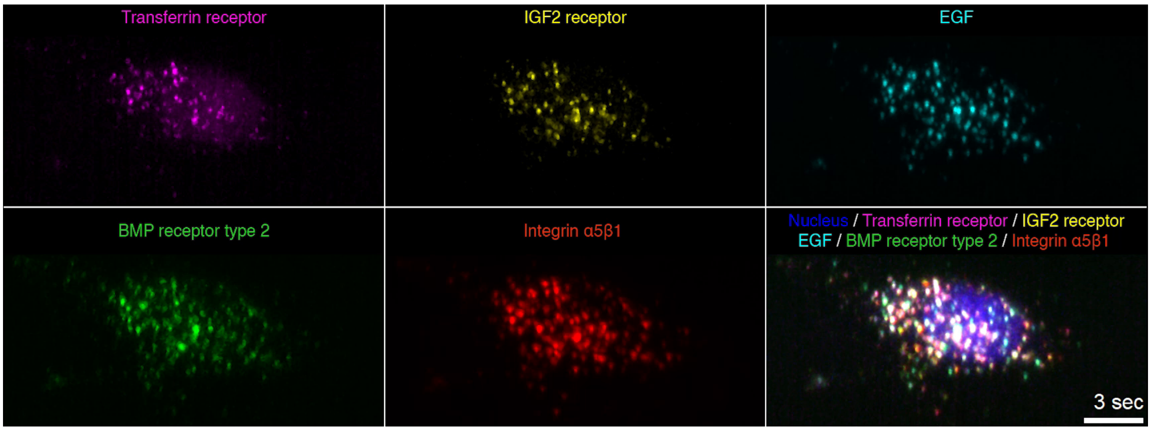

Supplementary Video 14: Fast multispectral live-cell light sheet imaging of intracellular trafficking using de-novo designed receptor minibinders.

Relates to Figure 6. TagBFP-NLS-expressing HeLa Kyoto cell loaded with four fluorescently-labelled, computationally designed minibinders against cell surface receptors (TfR, IGF2R, BMPR2 & Iα5β1) and one fluorescently-labelled receptor ligand (EGF) were imaged live by multispectral oblique plane light sheet microscopy. Images correspond to maximum intensity projections of the entire cell volume (47 planes), and volumetric imaging is performed at 3 Hz. Note that the fast aquisition speed allows tracking of all endosomes in the cell in 3D without motion blur even for fast-moving organelles. Also note the absence of colour misregistration caused by delays in acquiring colour channels in typical sequential aquisition schemes. Scale bar = 10 µm.
